# Molecular portrait of cisplatin induced response in human testis cancer cell lines based on gene expression profiles

**DOI:** 10.1186/1476-4598-6-53

**Published:** 2007-08-21

**Authors:** Nur Duale, Birgitte Lindeman, Mitsuko Komada, Ann-Karin Olsen, Ashild Andreassen, Erik J Soderlund, Gunnar Brunborg

**Affiliations:** 1 Department of Chemical Toxicology, Division of Environmental Medicine, Norwegian Institute of Public Health, Oslo, Norway

## Abstract

**Background:**

Testicular germ cell tumors (TGCTs) respond well to cisplatin-based chemotherapy and show a low incidence of acquired resistance compared to most somatic tumors. The reasons for these specific characteristics are not known in detail but seem to be multifactorial. We have studied gene expression profiles of testicular and colon cancer derived cell lines treated with cisplatin. The main goal of this study was to identify novel gene expression profiles with their functional categories and the biochemical pathways that are associated with TGCT cells' response to cisplatin.

**Results:**

Genes that were differentially expressed between the TGCT cell lines vs the (somatic) HCT116 cell line, after cisplatin treatment, were identified using the significance analysis of microarrays (SAM) method. The response of TGCT cells was strikingly different from that of HCT116, and we identified 1794 genes that were differentially expressed. Functional classification of these genes showed that they participate in a variety of different and widely distributed functional categories and biochemical pathways. Database mining showed significant association of genes (n = 41) induced by cisplatin in our study, and genes previously reported to by expressed in differentiated TGCT cells. We identified 37 p53-responsive genes that were altered after cisplatin exposure. We also identified 40 target genes for two microRNAs, hsa-mir-372 and 373 that may interfere with p53 signaling in TGCTs. The tumor suppressor genes *NEO1 *and *LATS2*, and the estrogen receptor gene *ESR1*, all have binding sites for p53 and hsa-mir-372/373. *NEO1 *and *LATS2 *were down-regulated in TGCT cells following cisplatin exposure, while *ESR1 *was up-regulated in TGCT cells. Cisplatin-induced genes associated with terminal growth arrest through senescence were identified, indicating associations which were not previously described for TGCT cells.

**Conclusion:**

By linking our gene expression data to publicly available databases and literature, we provide a global pattern of cisplatin induced cellular response that is specific for testicular cancer cell lines. We have identified cisplatin-responsive functional classes and pathways, such as the angiogenesis, Wnt, integrin, and cadherin signaling pathways. The identification of differentially expressed genes in this study may contribute to a better understanding of the unusual sensitivity of TGCT to some DNA-damaging agents.

## Background

Testicular germ cell tumors (TGCTs) are the most common tumors among young men. Fortunately, they respond well to cisplatin (Cis-Diamminedichloroplatinum (II) or CDDP)-based chemotherapy and there is a low incidence of acquired resistance for TGCT compared to most somatic tumors. More than 80% of all TGCTs with metastatic disease are curable using cisplatin-based chemotherapy [1]. TGCT are histologically classified as seminomas or non-seminomas, both originating from a common precursor known as carcinoma *in situ *(also known as intratubular germ cell neoplasia of the unclassified type) [2-4]. TGCT derived cell lines have often been used as model for studying cisplatin response [5,6]. The cause of their extreme sensitivity to chemotherapy seems to be multifactorial. TGCTs are "prone to apoptosis" and some studies have reported high levels of the pro-apoptotic Bax protein and low levels of the anti-apoptotic Bcl-2 protein (high Bax:Bcl-2 ratio), and elevated wild-type p53 function [7-10]. There are, however, conflicting reports on the role of the p53 status. The sensitivity toward cisplatin may also be an inherent property of primordial germ cells (PGCs) or gonocytes which are likely precursor cells for TGCTs [11]. Besides cisplatin, TGCTs are highly sensitive also to other chemotherapeutic drugs such as etoposide, ifosfamide, bleomycin, and vinblastine [12]. Similar to these agents, cisplatin is a DNA-damaging drug; cisplatin binds to form both intra- and inter-strand cross-links, and is thought to exert its cytotoxic effects through irreversible binding with DNA. One would hence expect variation in DNA repair capacities to be of importance for cell-type specific cisplatin sensitivity. Some studies have described a reduced ability of TGCT cells to repair cisplatin-induced DNA lesions, which is associated with a reduced expression level of several nucleotide excision repair (NER) proteins [13,14]. Cisplatin-adducts are removed from DNA mainly by NER [15]. In addition, It has been suggested that testis specific high-mobility group domain proteins such as SRY (testis-determining factor gene) may shield the cisplatin-induced DNA lesions from DNA repair proteins [16,17].

We have previously reported on DNA repair capacities in normal male germ cells. NER seems to be low in normal male germ cells compared to somatic cells [18], whereas base excision repair (BER) of oxidative lesions appears to be inefficient in testicular cells from humans but not in rodents [19]. These properties may have implications for the sensitivity to chemotherapeutics such as cisplatin, but are also of interest for the identification of environmental agent(s). In both cases, the global analysis of the response of genes after a toxic insult (toxicogenomics) provide an opportunity to study complex interactions. It is expected that a given toxicant will induce a distinct pattern of gene expression within its target cell or tissue, which can be used to identify and understand (cell type specific) toxic effects [20-22].

In this study we have analyzed cisplatin-induced gene expression in two well-characterized human testicular germ cell tumor (TGCT) derived cell lines (833K and GCT27) which both are sensitive to cisplatin, and a human colon carcinoma cell line (HCT116). We further evaluated the testicular germ cell tumor cells' specificity of response, by mining available public databases and literature. The statistical technique SAM was used to identify signature genes whose mRNA levels were significantly and differentially expressed between TGCT and HCT116 cells upon cisplatin treatment. We have identified discriminatory gene expression profiles that distinguish TGCT cells from the somatic HCT116 cell line. Genes identified to be significantly expressed were mapped by means of the Gene Ontology (GO) [23] and the Panther biochemical pathway [24] to obtain biological interpretations of the microarray data. We report here the identification of pathways and functional categories associated with cellular response to cisplatin. Besides previously identified pathways, we have identified new pathways that are likely to be relevant for the cisplatin mode of action. We have identified cisplatin induced p53-responsive, apoptosis-related, and senescence-like or terminal differentiation associated genes in TGCT cells. The knowledge extracted from the gene expression regulation and biological pathways in this way can be applied to elucidate the unique biology underlying the specific response of testicular tumors to cisplatin-based chemotherapy. Furthermore, this information may facilitate the identification of toxic compounds interfering with the immature male reproductive system.

## Results

### Cell cycle response of cisplatin-exposed cells

Unlike most somatic tumors, even metastatic TGCTs are usually cured by cisplatin-based chemotherapy. Cisplatin is a well characterized DNA-reactive agent, and it does not require metabolic activation. We used TGCT-derived cell lines in the search for gene expression signatures that characterize their response to lower doses of cisplatin. The doses used is in a concentration range reported to be clinically achievable for cisplatin [25]. Two treatment periods were 24 or 48 h, whereafter cells were immediately harvested for RNA isolation. At least two concentration levels were tested for each cell type; a low concentration that induced limited cell death and a higher concentration that induced profound cell cycle arrest in TGCTs (flow cytometric analysis). For 833K and GCT27 these were 0.3 μM (0.1 μg/ml) and 1.3 μM (0.4 μg/ml), and for HCT116 1.3 μM and 6.7 μM (2.0 μg/ml). The higher doses gave a delay in S-phase and a clear G2/M-arrest (~70 -80%) in all three cell lines after 48 h. No G1-arrest was observed in 833K and GCT27 cells (data not shown).

### Functional analysis and identification of discriminatory gene expression profiles after cisplatin exposure

We have used two-class, unpaired SAM analysis to select genes whose mean expression level is consistently significantly different between TGCT and HCT116 cell lines after cisplatin treatment. The number of genes that were consistently differentially expressed (FDR < 1% with delta = 3.8) was 1794 genes. 1180 of these genes were over-expressed in TGCT cells and under-expressed in HCT116 cells, whereas 614 genes were under-expressed in TGCT cells and over-expressed in HCT116 cells (Additional file 1). When the two TGCT cell lines were compared with each other, using the same SAM parameters (FDR < 1%, delta = 3.8), only one gene was found to be significantly differentially expressed. This also shows that the response of the two TGCT cells lines is similar and different from that of HCT116 cells.

**Figure 1:**
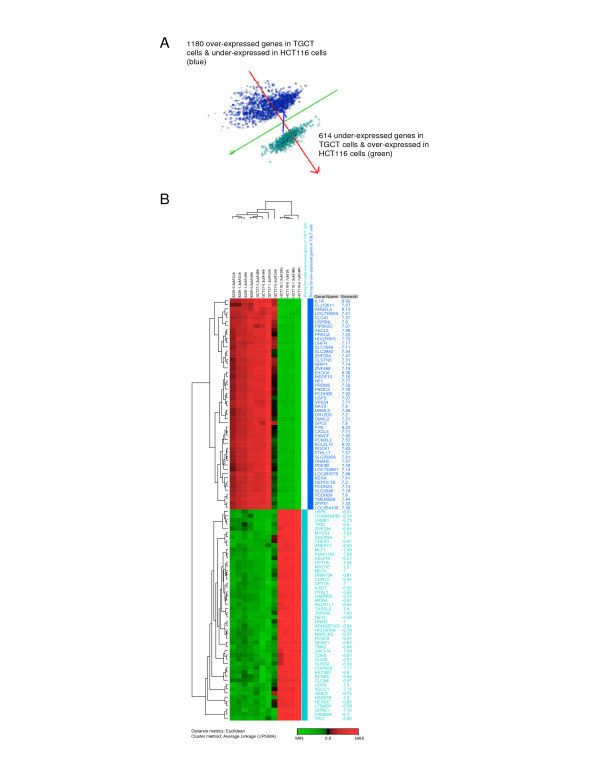
**Principle component analysis (PCA) and hierarchical clustering analysis of SAM-identified genes**. A) PCA of SAM identified genes that discriminate TGCT cells from HCT116 cells (n = 1794); 1180 up-regulated in TGCT cells and 614 genes up-regulated in HCT116 cells. Blue dots represent the 1180 up-regulated genes in TGCT cells and the green dots represent the 614 up-regulated in HCT116 cells, and vice versa. The complete list from SAM analysis is submitted as Additional file 1. B) Hierarchical clustering analysis of the top 50 over- and under-expressed genes from the SAM identified gene list. Genes are color coded based on the group they belong to.

Principal Component Analysis (PCA) on average log2-ratio of the 1794 genes shows a clear difference between TGCT and HCT116 cells (Figure 1A). The hierarchical clustering analysis of the top 50 over- and under-expressed genes from the SAM-identified genes (n = 1794) is represented in Figure 1B. The clustering dendrogram indicates that the TGCT cell lines are clustered close to each other. Examples of some consistently over-expressed genes in cisplatin exposed TGCT cells are *IL1A, EXOC6, FYN, ANGEL2, BCL2L10, ASCL3, PCDHB5, LOC283075, ROCK1*, and *USP6NL;*, the under-expressed genes include *LRP6, CDKN2B, MYST4, ZNF174, MLF1, CPT1B, TARSL2, UCP2, KIAA1160*, and *MYO1E*. The gene expression profiles constituted by these altered genes can be used as a basis for identification of candidate genes that may be involved in the biology of the specific high sensitivity of TGCT cells toward cisplatin exposure.

**Figure 2:**
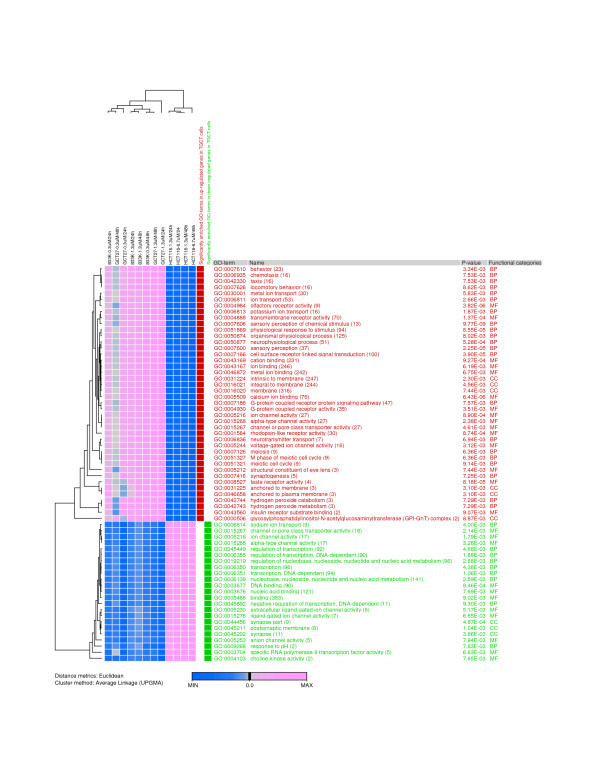
**Functional categories affected by cisplatin exposure of TGCT cells**. Hierarchical clustering analysis of significantly enriched (p < 0.01) GO-terms. SAM-identified differentially expressed genes (n = 1794) were grouped into gene sets based on common biological processes, molecular functions, or cellular components as assessed by the GO database. The average gene expression ratios of the gene sets in each GO term were used to construct the heat-map. Color coding: Over-represented GO-terms in the over-expressed gene sets (red), and over-represented terms in the under-expressed gene sets (green).

To determine the biological relevance of the SAM-identified genes, we have investigated their cellular functions using eGOn v2.0 [26,27] and Panther [24,28]. The eGOn uses Fisher's exact test and allows determination of significantly enriched GO terms within a gene set, compared with the frequency among genes on the array. We correlated SAM-identified genes with the following functional categories: *biological process*, *molecular function, and cellular component*. This analysis aimed at an understanding of the effect of the modulation of gene expression on a particular cellular function. eGOn results represent a global picture of biological processes, molecular functions and cellular co-localizations that are significantly enriched following cisplatin treatment. Sixty-five GO categories were identified as significantly enriched (p < 0.01) among the genes identified by SAM as differentially expressed. Figure 2 shows the average gene expression ratios of these gene sets in each GO term; they were used to construct the heat-map. The GO terms assigned to the significantly altered genes are listed in Additional file 2.

The most significantly enriched (p < 0.01) *biological process *gene categories include genes related to sensory perception, cell surface receptor linked signal transduction, physiological response to stimulus, neurophysiological process, and potassium ion transport ; genes in these categories were over-expressed in TGCT cells following cisplatin exposure. Significantly enriched (p < 0.01) *biological processes *among the under-expressed genes included those involved in transcription; nucleobase, nucleoside, nucleotide and nucleic acid metabolism; sodium ion transport; and response to pH (Figure 2).

Significantly enriched (p < 0.01) *molecular function *gene categories include genes related to olfactory receptor activity, calcium ion binding, taste receptor activity, and transmembrane receptor activity. The gene sets in these GO categories were up-regulated in TGCT cells following cisplatin exposure. Significantly enriched *molecular functions *among the down-regulated genes include those involved in DNA binding, channel or pore class transporter activity, extracellular ligand-gated ion channel activity, specific RNA polymerase II transcription factor activity, and ligand-gated ion channel activity (Figure 2). Taken together, cisplatin seems to significantly interfere with processes associated with transcription and DNA metabolism.

**Figure 3:**
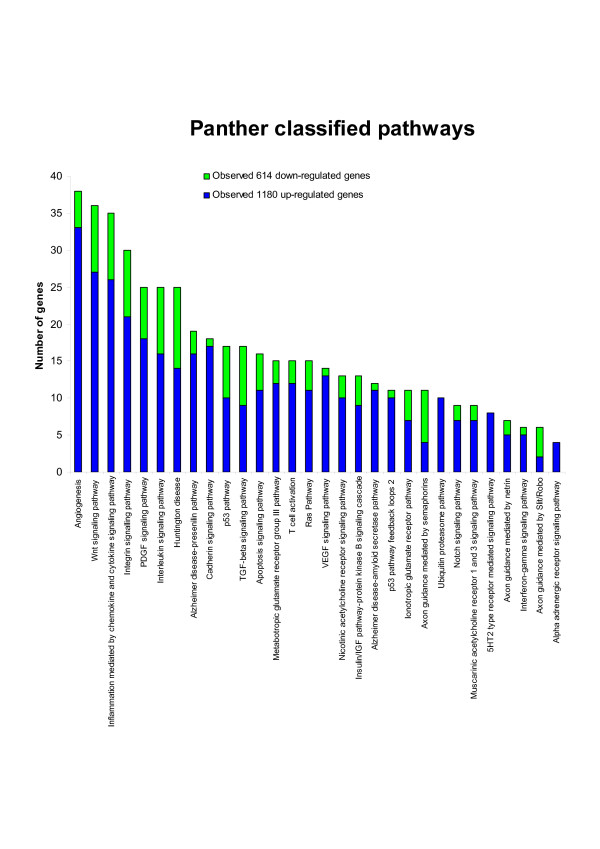
**Panther pathways affected by cisplatin exposure of TGCT cells**. Significantly enriched (p < 0.05) Panther pathways in over- and under-expressed genes following cisplatin exposure. Blue bars represent the number of over-expressed genes in the pathway, green bars the number of under-expressed genes.

To identify biochemical pathways that had been affected by cisplatin gene expression modulation, we analyzed SAM-significant genes by Panther [24,28]. Panther uses the binomial statistics tool to determine over- or under- representation of Panther classification categories. Panther determines functional clusters by representation of individual genes in specific categories relative to all genes in the same category on the array [24]. The most significantly over-represented pathways (p < 0.05) were angiogenesis, Wnt signaling pathway, inflammation mediated by chemokine and cytokine signaling pathway, integrin signalling pathway, PDGF signaling pathway, interleukin signaling pathway, Huntington disease, Alzheimer disease-presenilin pathway, cadherin signaling pathway, p53 pathway, TGF-beta signaling pathway, and apoptosis signaling pathway; these are shown in Figure 3. The identified pathways are over-represented mainly with gene sets that are up-regulated in TGCT cells following cisplatin exposure. Panther pathways significantly over-represented in down-regulated genes in TGCT cells are involved in TGF-beta signaling pathway, and axon guidance mediated by semaphorins and by Slit/Robo (Figure 3). Interleukin signaling pathway and Huntington disease pathway were over-represented in both up- and down-regulated genes. From this analysis, we have identified new functional categories and biochemical pathways which have not previously been shown to be associated with cisplatin-induced cellular response in TGCT cells.

### *In silico *analysis of significantly altered genes

**Figure 4:**
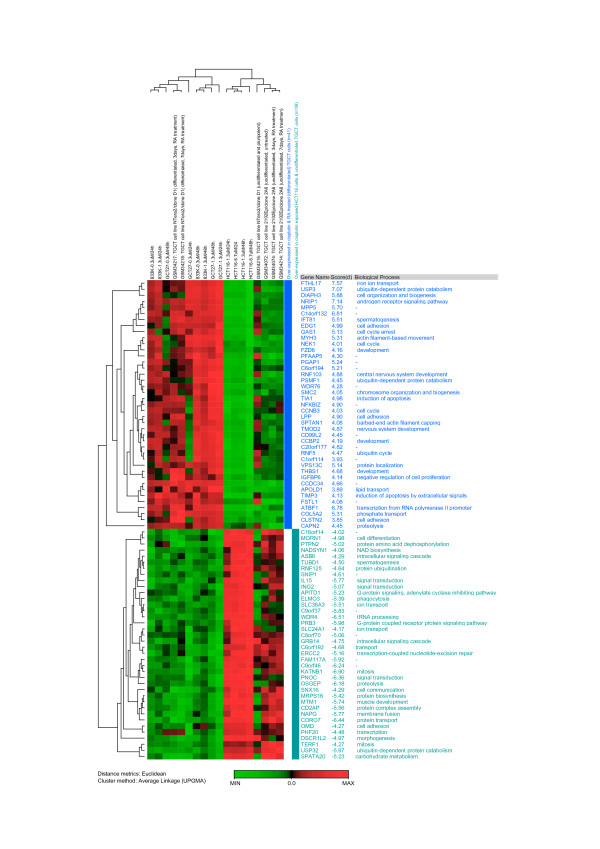
**Hierarchical clustering analysis of 79 genes**. These genes have been identified as being commonly expressed in microarray data from GSE1818, and in our SAM identified genes. The dendrogram shows that cisplatin exposed TGCT cells and RA-treated differentiated TGCT cells are closely clustered. Genes are colored coded according to their expression patterns.

A search was performed in the publicly available databases in order to compare the gene expression of our SAM-identified gene list to those reported for other systems. We used the microarray data from Skotheim et al. (2005) (GEO accession number GSE1818), which is a study of germ cell gene expression measured in normal and tumor human testis biopsies and TGCT cells [29]. They studied retinoic acid (RA)-induced *in vitro *differentiation of two other TGCT cell lines, a pluri-potent NTERA2 cell line, and a nulli-potent 2102Ep cell line (NTERA2 cells differentia upon RA treatment whereas 2102Ep does not). We conducted similarity searches between our SAM-identified gene expression data and microarray data from GSE1818 (only the microarray data from RA-treated and untreated TGCT cells (NTREA2 and 2102Ep) were used). We selected 79 genes that showed similar expression patterns in our cisplatin treated as in the RA-treated the TGCT cell line NTREA2. Of those genes, 41 were up-regulated and 38 were down-regulated genes in TGCT cells following cisplatin exposure. Figure 4 shows hierarchical clustering analysis of these 79 genes. By visual inspection of the heat-map (Figure 4), we observed that cisplatin treated TGCT cells and RA-induced differentiated TGCT cells were clustered together, and they showed similar expression pattern.

From the database mining comparison, we conclude that cisplatin exposed TGCT cells and RA-induced differentiated TGCT cells appear to share distinct gene expression patterns. A significant number of the genes altered in response to cisplatin were among those highly expressed also in the RA-induced differentiated TGCT cells; this is unlike the pattern for the somatic HCT116 cell line and the undifferentiated TGCT cells [29].

### *In silico *prediction of p53

The tumor suppressor gene p53 is an important transcription factor activated by DNA damage. Although several reports underline the importance of p53-siganling pathway in cisplatin response in TGCT cells, other studies have reported a partially non-functional p53-signaling pathway [30-37]. With the hypothesis that p53-associated target genes are heavily involved in cisplatin response, we analyzed the SAM-identified gene list with respect to genes possessing a predicted or known p53 binding site in the promoter region. For this purpose we searched the literature, the Transcriptional regulatory element database (TRED) [38], and Transcription factor binding profile database (JASPAR) [39,40]. Table 1 shows that 37 p53 target genes were found in our gene list, of which 26 genes were up-regulated and 11 genes were down-regulated in TGCT cells upon cisplatin exposure. All of these target genes have consensus p53-responsive elements upstream of their predicted transcriptional start site, according to the JASPAR-database.

**Table 1:** List of p53 responsive genes (n = 37). 26 genes were up-regulated and 11 genes were down-regulated in the TGCT cells following cisplatin exposure

**UniGene ID**	**Gene Name**	**Description**	**SAM Score(d)**	**Referance (a)**	**p53 binding motifs (b)**
**A) 26 p53 target genes that are up-regulated in TGCT cells and down-regulated in HCT116 cells**					
Hs.306322	**NAV3**	Neuron navigator 3	7.60	*2*	+
Hs.553498	**PIK3CA**	Phosphoinositide-3-kinase, catalytic, alpha polypeptide	6.63	*1*	+
Hs.196054	**HDAC9**	Histone deacetylase 9	6.44	*2*	+
Hs.395482	**PTK2**	PTK2 protein tyrosine kinase 2	6.30	*2*	+
Hs.501778	**TRIM22**	Tripartite motif-containing 22	6.11	*2,3&4*	+
Hs.160871	**PTPRO**	Protein tyrosine phosphatase, receptor type, O	5.96	*2*	+
Hs.495473	**NOTCH1**	Notch homolog 1, translocation-associated (Drosophila)	5.73	*2*	+
Hs.591292	**GPR87**	G protein-coupled receptor 87	5.59	*3*	+
Hs.13291	**CCNG2**	Cyclin G2	5.27	*2*	+
Hs.65029	**GAS1**	Growth arrest-specific 1	5.13	*1*	+
Hs.592020	**IGF1R**	Insulin-like growth factor 1 receptor	5.09	*1*	+
Hs.591630	**CASP8**	Caspase 8, apoptosis-related cysteine peptidase	5.04	*1*	+
Hs.138211	**MAPK8**	Mitogen-activated protein kinase 8	5.03	*1*	+
Hs.89679	**IL2**	Interleukin 2	5.02	*1*	+
Hs.93842	**STARD4**	START domain containing 4, sterol regulated	4.95	*2*	+
Hs.326035	**EGR1**	Early growth response 1	4.90	*1*	+
Hs.40582	**CDC14B**	CDC14 cell division cycle 14 homolog B (S. cerevisiae)	4.52	*1*	+
Hs.634224	**PIAS2**	Protein inhibitor of activated STAT, 2	4.43	*2*	+
Hs.648190	**ASTN2**	Astrotactin 2 V-erb-a erythroblastic leukemia viral oncogene homolog 4	4.34	*2*	+
Hs.390729	**ERBB4**	(avian)	4.28	*2*	+
Hs.476018	**CTNNB1**	Catenin (cadherin-associated protein), beta 1, 88 kDa	4.22	*1*	+
Hs.591179	**DGKE**	Diacylglycerol kinase, epsilon 64 kDa	4.21	*1*	+
Hs.35947	**MBD4**	Methyl-CpG binding domain protein 4 SWI/SNF related, matrix associated, actin dependent	4.05	*1*	+
Hs.534350	**SMARCB1**	regulator of chromatin, subfamily b, member 1	4.03	*2*	+
Hs.545196	**GML**	GPI anchored molecule like protein	3.41	*1&2*	+
Hs.208124	ESR1*	Estrogen receptor 1	3.04	*1*	+
**B) 11 p53 target genes that are down-regulated in TGCT cells and up-regulated in HCT116 cells**					
Hs.388613	NEO1*	Neogenin homolog 1 (chicken)	-7.00	*2*	+
Hs.124922	**LRMP**	Lymphoid-restricted membrane protein	-5.99	*4*	+
Hs.168132	**IL15**	Interleukin 15	-5.77	*1*	+
Hs.256126	**BIRC7**	Baculoviral IAP repeat-containing 7 (livin)	-5.59	*1*	+
Hs.76171	**CEBPA**	CCAAT/enhancer binding protein (C/EBP), alpha	-5.45	*1*	+
Hs.78960	LATS2*	LATS, large tumor suppressor, homolog 2 (Drosophila)	-4.54	*2*	+
Hs.271955	**TNFAIP8**	Tumor necrosis factor, alpha-induced protein 8	-4.46	*2*	+
Hs.397465	**HIPK2**	Homeodomain interacting protein kinase 2	-4.37	*1*	+
Hs.291363	**CHEK2**	CHK2 checkpoint homolog (S. pombe)	-4.26	*1*	+
Hs.508423	**ABCC4**	ATP-binding cassette, sub-family C (CFTR/MRP), member 4 Solute carrier family 7 (cationic amino acid transporter, y+	-4.06	*1*	+
Hs.334848	**SLC7A6**	system), member 6	-3.95	*4*	+

a) *Predicted and known p53 target genes based on following references (1) Zhao et al. (2005) *[38]*, (2) Wei et al. (2006) *[80]*, (3) Kerley-Hamilton et al. (2005) *[35]*and (4) Barenco et al. (2006) *[81]*). b) These genes have a consensus p53-responsive element (MA0106) upstream of their predicted transcriptional start site according to the *JASPAR database [39,40].* NEO1, LATS2 and ESR1 are the only target genes common for both p53 and hsa- mir-372 and 373.

Recently, Voorhoeve and co-workers identified two microRNAs (miRNA), hsa-mir-372 and hsa-mir-373 that are highly expressed in TGCTs and TGCT derived cell lines, and it was reported that these miRNAs suppressed elements of the p53 pathway in TGCT cells. We used the target prediction programs, PicTar and TargetScan 3.1 [41,42], to identify possible targets of hsa-mir-372 and 373. We found that 40 genes (24 up-regulated and 16 down-regulated in TGCT cells), among our SAM-identified gene list were predicted target genes for both hsa-mir-372 and 373 (Table 2).

**Table 2:** List of SAM-identified genes (n = 40) predicted as target for hsa-mir-372 and 373

**UniGene ID**	**Gene Name**	**Description**	**SAM score(d)**
**A) 24 miR-372&373 target genes that are up-regulated in TGCT cells and down-regulated in HCT116 cells**			
**UniGene ID**	**Gene_symbol**	**Gene_description**	**Score(d)**
Hs.280604	**PPP3R1**	Protein phosphatase 3 (formerly 2B), regulatory subunit B, 19 kDa, alpha isoform	6.48
Hs.201034	**NTN4**	Netrin 4	6.35
Hs.126497	**AEBP2**	AE binding protein 2	6.18
Hs.373857	**KLF12**	Kruppel-like factor 12	6.06
Hs.514242	**PLEKHM1**	Pleckstrin homology domain containing, family M (with RUN domain) member 1	5.93
Hs.499489	**MARCH8**	Membrane-associated ring finger (C3HC4) 8	5.63
Hs.349150	**PURB**	Purine-rich element binding protein B	5.55
Hs.25960	**MYCN**	V-myc myelocytomatosis viral related oncogene, neuroblastoma derived (avian)	5.25
Hs.474536	**MTMR3**	Myotubularin related protein 3	5.24
Hs.291623	**TAOK2**	TAO kinase 2	5.24
Hs.271341	**RABGAP1**	RAB GTPase activating protein 1	5.13
Hs.134221	**MBNL2**	Muscleblind-like 2 (Drosophila)	4.92
Hs.520187	**PYCR2**	Pyrroline-5-carboxylate reductase family, member 2	4.78
Hs.490347	**MKRN1**	Makorin, ring finger protein, 1	4.75
Hs.191179	**RAB11FIP1**	RAB11 family interacting protein 1 (class I)	4.67
Hs.567754	**MIER3**	Mesoderm induction early response 1, family member 3	4.48
Hs.4779	**GATAD2B**	GATA zinc finger domain containing 2B	4.34
Hs.159430	**FNDC3B**	Fibronectin type III domain containing 3B	4.20
Hs.632239	**MNT**	MAX binding protein	4.16
Hs.591552	**RAB6A**	RAB6A, member RAS oncogene family	4.15
Hs.434961	**ATXN1**	Ataxin 1	4.06
Hs.149261	**RUNX1**	Runt-related transcription factor 1 (acute myeloid leukemia 1; aml1 oncogene)	3.69
Hs.497148	**RGL1**	Ral guanine nucleotide dissociation stimulator-like 1	3.19
Hs.208124	ESR1*	Estrogen receptor 1	3.04
**B) 16 miR-372&373 target genes that are down-regulated in TGCT cells and up-regulated in HCT116 cells**			
Hs.293798	**ZNF436**	Zinc finger protein 436	-7.05
Hs.388613	NEO1*	Neogenin homolog 1 (chicken)	-7.00
Hs.374097	**IRF2**	Interferon regulatory factor 2	-6.13
Hs.122927	**RSNL2**	Restin-like 2	-6.09
Hs.514308	**FAM117A**	Family with sequence similarity 117, member A	-5.92
Hs.444213	**TLE4**	Transducin-like enhancer of split 4 (E(sp1) homolog, Drosophila)	-5.92
Hs.428027	**PBX3**	Pre-B-cell leukemia transcription factor 3	-5.78
Hs.511316	**GABPB2**	GA binding protein transcription factor, beta subunit 2	-5.52
Hs.529044	**RAB22A**	RAB22A, member RAS oncogene family	-5.45
Hs.434993	**RBJ**	Ras-associated protein Rap1	-5.28
Hs.496138	**IQSEC2**	IQ motif and Sec7 domain 2	-4.70
Hs.78960	LATS2*	LATS, large tumor suppressor, homolog 2 (Drosophila)	-4.54
Hs.269592	**CC2D1A**	Coiled-coil and C2 domain containing 1A	-4.21
Hs.521083	**HRBL**	HIV-1 Rev binding protein-like	-4.19
Hs.469658	**BCL2L11**	BCL2-like 11 (apoptosis facilitator)	-4.08
Hs.299315	**DPYSL5**	Dihydropyrimidinase-like 5	-3.93

NEO1, LATS2 and ESR1 are the only target genes in common for both p53 and hsa-mir-372 and -373.

From the SAM-identified gene list we found that Neogenin homolog 1 (*NEO1*), large tumor suppressor homolog 2 (*LATS2*) and Estrogen receptor 1 (*ESR1*) were the only genes that have the predicted binding sites for both p53 and hsa-mir-372 and 373. *NEO1 *and *LATS2 *were down-regulated in the TGCT cells cisplatin exposure, while *ESR1 *was up-regulated.

### Apoptosis-related genes altered following cisplatin exposure

Among the SAM-identified genes, we found 24 apoptosis-related genes (Table 3). In the TGCT cells, the cisplatin exposure led to increased gene expression of *IL1A, BCL2L10, BCL2L13, MPO, CCL2, CASP8, IL2, CARD6, P53AIP1, BAG5, DOCK1, CAPN1, CAPN2, STK17B *and *GML*. However, the gene expression levels of 9 apoptosis-related genes, *BAG4, BIRC7, MXD4, APITD1, ING2, PAWR, PDCD6, BCL2L11 and TNFAIP8*, were significantly decreased in the cisplatin exposed TGCT cells.

**Table 3:** List of apoptosis-related genes (n = 24). 15 up-regulated and 9 down-regulated genes in TGCT cells following cisplatin exposure

**UniGene ID**	**Gene Name**	**Description**	**SAM Score(d)**	**Function**
**A) 15 apoptosis-related genes that are up-regulated in TGCT cells and down-regulated in HCT116 cells**				
Hs.1722	**IL1A**	Interleukin 1, alpha	8.62	anti-apoptosis
Hs.283672	**BCL2L10**	BCL2-like 10 (apoptosis facilitator)	8.03	anti-apoptosis
Hs.631672	**BCL2L13**	BCL2-like 13 (apoptosis facilitator)	6.51	Regulation of apoptosis
Hs.458272	**MPO**	Myeloperoxidase	5.96	anti-apoptosis
Hs.303649	**CCL2**	Chemokine (C-C motif) ligand 2	5.78	anti-apoptosis
Hs.591630	**CASP8**	Caspase 8, apoptosis-related cysteine peptidase	5.04	apoptotic program
Hs.89679	**IL2**	Interleukin 2	5.02	anti-apoptosis
Hs.200242	**CARD6**	Caspase recruitment domain family, member 6	4.83	regulation of apoptosis
Hs.160953	**P53AIP1**	P53-regulated apoptosis-inducing protein 1	4.65	apoptosis
Hs.5443	**BAG5**	BCL2-associated athanogene 5	4.57	anti-apoptosis
Hs.350899	**CAPN2**	Calpain 2, (m/II) large subunit	4.45	proteolysis and peptidolysis
Hs.159195	**DOCK1**	Dedicator of cytokinesis 1	4.33	Apoptosis
Hs.521800	**CAPN1**	Calpain 1, (mu/I) large subunit	3.97	proteolysis and peptidolysis
Hs.88297	**STK17B**	Serine/threonine kinase 17b (apoptosis-inducing)	3.62	apoptosis
Hs.545196	**GML**	GPI anchored molecule like protein	3.41	apoptosis
**B) 9 apoptosis-related genes that are down-regulated in TGCT cells and up-regulated in HCT116 cells**				
Hs.194726	**BAG4**	BCL2-associated athanogene 4	-6.33	anti-apoptosis
Hs.256126	**BIRC7**	Baculoviral IAP repeat-containing 7 (livin)	-5.59	anti-apoptosis
Hs.102402	**MXD4**	MAX dimerization protein 4	-5.59	negative regulation of cell proliferation
Hs.412311	**APITD1**	Apoptosis-inducing, TAF9-like domain 1	-5.23	arole in a cell death pathway
Hs.107153	**ING2**	Inhibitor of growth family, member 2	-5.07	P53-dependent apoptotic pathways
Hs.643130	**PAWR**	PRKC, apoptosis, WT1, regulator	-4.99	apoptosis
Hs.379186	**PDCD6**	Programmed cell death 6	-4.79	Induction of apoptosis
Hs.271955	**TNFAIP8**	Tumor necrosis factor, alpha-induced protein 8	-4.46	anti-apoptosis
Hs.469658	**BCL2L11**	BCL2-like 11 (apoptosis facilitator)	-4.08	apoptosis

### Cellular senescence-related genes altered following cisplatin exposure

Terminal differentiation or senescence is an important response to low doses of chemotherapy agents. The over-expression of several male germ cell specific (testis specific) genes in TGCT cells may indicate differentiation in response to cisplatin. We hence performed a literature mining in order to identify reported senescence-related genes in other systems. Among the SAM-identified genes, 5 genes proved to be in common with those reported by Schwarze and co-workers who studied gene expression in terminally differentiated human prostate epithelial cells (HPECs) compared to proliferating cells [43]. By comparing with the Chang et al. (2001) study, in which gene expression in senescent relative to proliferating cells treated with doxorubicin [44] were studied, we identified 3 genes in common in our SAM-identified gene list. We have observed up-regulation of some testis specific genes such as *SPAM1, SPATA22, TCAM1, SPAG17, SOX17 *and *SOX7 *in TGCT cells. Table 4 shows 29 senescence or differentiation related genes induced in response to cisplatin in this study.

**Table 4:** Terminal differentiation or senescence related genes (n = 29) induced in TGCT cells following cisplatin exposure

**UniGene ID**	**Gene Name**	**Description**	**SAM Score(d)**
**A) 5 genes from Schwarze et al (2002)**			
Hs.1722	**IL1A**	Interleukin 1, alpha	8.62
Hs.138211	**MAPK8**	Mitogen-activated protein kinase 8	5.03
Hs.484551	**CPM**	Carboxypeptidase M	4.88
Hs.504877	**ARHGDIB**	Rho GDP dissociation inhibitor (GDI) beta	4.57
Hs.89649	**EPHX1**	Epoxide hydrolase 1, microsomal (xenobiotic)	4.52
**B) 3 genes from Chang et al (2001)**			
Hs.135646	**ELF1**	E74-like factor 1 (ets domain transcription factor)	5.47
Hs.134221	**MBNL2**	Muscleblind-like 2 (*Drosophila*)	4.92
Hs.224012	**JAG1**	Jagged 1 (Alagille syndrome)	4.00
**C) 6 Testis-specific genes**			
Hs.98367	**SOX17**	SRY (sex determining region Y)-box 17	6.36
Hs.351068	**SPATA22**	Spermatogenesis associated 22	6.23
Hs.528821	**SPAG17**	Sperm associated antigen 17	5.29
Hs.121494	**SPAM1**	Sperm adhesion molecule 1 (PH-20 hyaluronidase, zona pellucida binding)	4.94
Hs.213194	**SOX7**	SRY (sex determining region Y)-box 7	4.84
Hs.629177	**TCAM1**	Testicular cell adhesion molecule 1 homolog (mouse)	4.67
**D) 2 Germ cell development related genes**			
Hs.632137	**CUGBP1**	CUG triplet repeat, RNA binding protein 1	4.78
Hs.436445	**OSGIN2**	Oxidative stress induced growth inhibitor family member 2	4.59
**E) Other differentiation or senescence related genes (n = 13)**			
Hs.119693	**PCDHB5**	Protocadherin beta 5	7.92
Hs.489142	**COL1A2**	Collagen, type I, alpha 2	6.48
Hs.372946	**LOC440895**	Similar to LIM and senescent cell antigen-like domains 3	5.98
Hs.55999	**NKX3-1**	NK3 transcription factor related, locus 1 (*Drosophila*)	5.52
Hs.54473	**NKX2-5**	NK2 transcription factor related, locus 5 (*Drosophila*)	5.08
Hs.154210	**EDG1**	Endothelial differentiation, sphingolipid G-protein-coupled receptor, 1	4.99
Hs.645216	**IGFBP7**	Insulin-like growth factor binding protein 7	4.99
Hs.522805	**CD99L2**	CD99 molecule-like 2	4.45
Hs.234763	**NKX2-8**	NK2 transcription factor related, locus 8 (*Drosophila*)	4.45
Hs.274313	**IGFBP6**	Insulin-like growth factor binding protein 6	4.14
Hs.528847	**MYADML**	Myeloid-associated differentiation marker-like	3.99
Hs.555902	**DDEF2**	Development and differentiation enhancing factor 2	3.88
Hs.106015	**DDEF1**	Development and differentiation enhancing factor 1	3.87

### Validation of microarray data by real-time PCR

**Figure 5:**
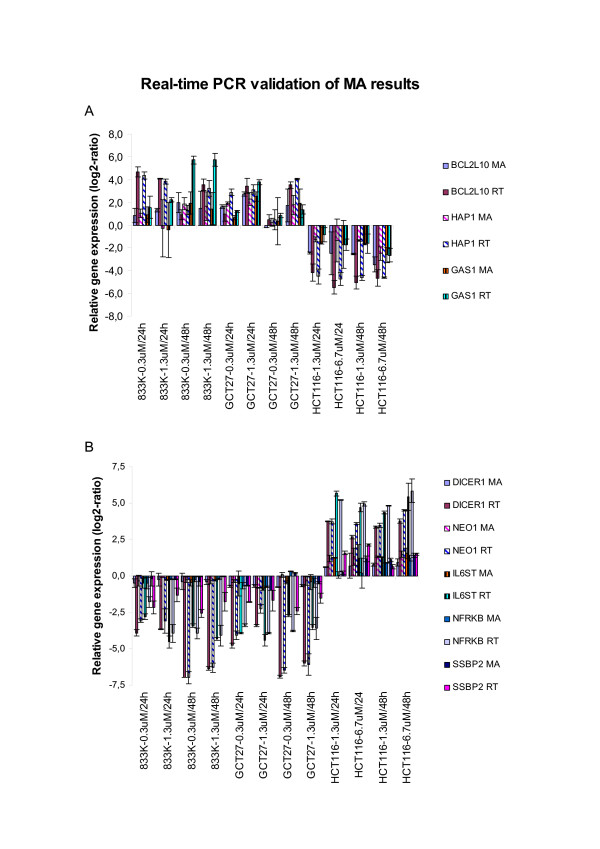
**Quantitative real-time PCR validation of microarray results. **Validation of selected 8 SAM-identified genes by quantitative real-time PCR. *BCL2L10, HAP1 *and *GAS1 *were up-regulated in TGCT cells and down-regulated in HCT116 cells following cisplatin exposure. *DICER1, NEO1, IL6ST, NFRKB and SSBP2 *were up-regulated in HCT116 cells and down-regulated in TGCT cells. The bars indicate the mean +/- S.E.M.

The results obtained from the microarray data analysis revealed an altered transcription of many genes following cisplatin treatment. To verify their expression, quantitative real-time RT-PCR was performed using the same RNA as that used in the microarray analysis. Eight selected genes from our SAM-identified genes were analyzed. Of those, *GAS1, HAP1*, and *BCL2L-10 *were up-regulated in TGCT cells and down-regulated in HCT116 cells, while *NEO1, DICER1, IL6ST, SSBP2 *and *NFRKB *were down-regulated in TGCT cells and up-regulated in HCT116 cells. The quantitative real-time PCR results for these genes were in good correlation (same direction) with the microarray results (Figure 5).

## Discussion

Microarray technology may facilitate the analysis of the mechanisms of action of drugs. The mRNA expression profile of drug-treated cells can readily be compared with untreated control cells to reveal those genes that have undergone a change in response to drug treatment. The main goals of this study were to identify novel gene expression profiles with their functional categories and the biochemical pathways that are associated with the TGCT cells' response to cisplatin-induced DNA-damage. The strategy was to identify statistically significantly altered genes that differentiate two testicular germ cell tumor cell lines from a somatic colon tumor cell line (HCT116) by using the SAM method. SAM is a robust statistical method that has been specifically developed for microarray data analysis [45]. By using two-class, unpaired SAM analysis, we have identified many genes that discriminate TGCT cells from HCT116 cells. The analysis of the SAM-identified (n = 1794) genes by unsupervised hierarchical clustering [46], showed that we could readily group TGCT cells based solely on the patterns of the their expression. Moreover, the Principal Component Analysis also demonstrated that there were clear gene expression pattern differences between TGCT and HCT116 cells used in the study. However, the task of organizing the clusters of significantly expressed genes for further biological interpretation and determining what kind of genes or pathways that are involved in mediating the effects of cisplatin remain as main challenges. Platinum based compounds are routinely used in the treatment of TGCTs in combination with bleomycin and etoposide. It is possible that these and other chemoterapeutics may interfere with the same pathways as we have identified using cisplatin; however, to our knowledge no relevant systematic study of expression patterns *in vitro *have been presented.

We have addressed the biological relevance of the differentially expressed genes through analysis of the GO terms and biochemical pathways utilizing eGOn [26,27] and Panther [24,28]. The result of these analyses are summarized in Figure 2 and 3. Interestingly, the most over-represented biological processes comprising the up-regulated genes were sensory perception, cell surface receptor linked signal transduction, physiological response to stimulus, neurophysiological process, and potassium ion transport. Among the down-regulated genes, there were genes related to transcription; nucleobase, nucleoside, nucleotide and nucleic acid metabolism; sodium ion transport; and response to pH. Predominating biochemical pathways found to be significantly over-represented in TGCT cells were angiogenesis, Wnt signaling pathway, inflammation mediated by chemokine and cytokine signaling pathway, integrin signalling pathway, PDGF signaling pathway, interleukin signaling pathway, Huntington disease, Alzheimer disease-presenilin pathway, cadherin signaling pathway, p53 pathway, TGF-beta signaling pathway, and apoptosis signaling pathway. Taken together, we have identified new functional classes and pathways that were not previously known to be associated with cisplatin treatment.

We compared our SAM-identified genes with publicly available micoarray data from Skotheim et al. (2005). These authors reported on germ cell gene expression in normal and tumor human testis biopsies; they also studied TGCT cells after RA treatment [29]. Among our cisplatin responsive genes, a subpopulation of 41 genes was identified that represents genes being over-expressed both in cisplatin exposed TGCT cells, and also in the RA-induced differentiated TGCT cell line [29]. The clustering dendrogram (Figure 4) shows that cisplatin responsive genes in TGCT cells and RA-induced differentiated TGCT cells were categorized into close branches and they had a similar expression pattern. Genes involved in GO categories such as cell adhesion, development, and spermatogenesis were abundant among the 41 highly expressed genes. RA-induced growth arrest of tumor cells is assumed to result from induction of differentiation and this has been corroborated by the appearance of differentiation specific markers in RA-treated cells [47]. Among the 41 over-expressed genes in differentiated and cisplatin exposed TGCT cells (Figure 4), there are genes that may be used as TGCT terminal differentiation markers.

The biochemical mechanisms underlying the extreme sensitivity of TGCTs for cisplatin are still partly unknown. Analysis of potential parameters in cisplatin sensitivity, including cellular detoxification mechanisms such as the glutathione and the metallothionein systems, platinum accumulation, DNA platination and repair, p53 status, and expression of Bcl-2 family proteins, have not been able to fully explain the inherent sensitivity of testicular tumors [11]. The p53 transcription factor is the key gatekeeper in the cellular response to stress signals. Different types of stress result in different post-translational modifications of p53 that lead to one of three different types of cellular response: cell cycle arrest, cellular senescence, or apoptosis [48,49]. There are conflicting reports on the role of p53 in cisplatin sensitivity of TGCTs. Several studies suggest that p53 is important [30-32], whereas other studies have shown that p53 status did not relate directly to the sensitivity of TGCT cells to cisplatin [33,34]. From literature and database mining, we found 37 p53 responsive genes in our SAM-identified gene list (Table 1). Functional classification of these 37 genes revealed that they were widely distributed among several functional categories; this is in agreement with the global gene regulation effects of p53. Kerley-Hamilton and co-workers have shown that TGCT cells have a specific p53-dependent transcriptional response to cisplatin treatment. They concluded that p53 is an important target of cisplatin-mediated gene expression and cytotoxicity in TGCT cells [35]. In our system, cisplatin exposure resulted in very high p53 phosphorylation, in the TGCT cells compared to the somatic cells such as HCT116 and MCF-7 (data not shown). This suggests that the signaling pathways upstream of p53 are highly responsive to cisplatin. It appears that the p53 signaling pathway and the feedback mechanisms controlling this pathway play an important role in the TGCT response toward cisplatin treatment.

Although testicular germ cell tumors exhibit wild-type p53, some studies indicate that p53 is non-functional or only partly functional. The newly discovered microRNAs; hsa-mir-372 and 373, have been reported to be over-expressed in TGCT cells but not in normal testis tissues. These miRNAs do not function by simply down-regulating p53 itself, but they block p53-mediated *CDK2 *(cyclin-dependent kinase 2) inhibition possibly through direct inhibition of the tumor suppressor gene *LATS2 *(large tumor suppressor homolog 2) [36]. Microarray analysis of hsa-mir-372/373-expressing cells has shown that *LATS2*, a known inhibitor of *CDK2*, was down-regulated, and the 3' UTR of LATS2 was shown to harbor predicted hsa-mir-372/373 binding sites. Inhibition of *LATS2 *expression by hsa-mir-372/373 relieves *CDK2 *from repression allowing continued cellular proliferation in the presence of activated Ras [36,50]. In this study, we observed down-regulation of *LATS2 *expression in TGCT cells following cisplatin exposure (Table 1 and 2).

It has been reported that specific miRNAs contribute to the regulation of cellular differentiation, proliferation and apoptosis. The expression of miRNAs is highly specific for tissues and developmental stages. Little is known about the regulation of miRNA [41,51,52]. The target prediction programs, PicTar [41] and TargetScan 3.1 [42], were used to identify possible targets of hsa-mir-372 and 373. We have identified 29 genes that were predicted target genes for both hsa-mir-372 and 373. As shown in Table 1 and 2, *NEO1, LATS2 *and *ESR1 *are the only predicted target gene for both hsa-mir-372 and 373 and p53; the 3' UTR of *NEO1*, *LATS2 *and *ESR1 *have potential hsa-mir-372 and 373 binding sites. *NEO1 *is functionally categorized as a tumor-suppressor gene and may be involved as a regulatory protein in the transition of undifferentiated proliferating cells to their differentiated state. *ESR1 *belongs to the nuclear hormone receptor family and is involved in the regulation of eukaryotic gene expression, affecting cellular proliferation and differentiation in target tissues. In our study, we observed that *NEO1 *and *LATS2 *were down-regulated in the TGCT cells, while ESR1 was up-regulated. Since *NEO1 *and *LATS2 *are tumor-suppressor genes and at the same time target genes for p53 and hsa-mir-372 and 373, both hsa-mir-372 and 373 may suppress the p53 pathway by destabilizing *NEO1*s and *LATS2*s transcript and/or by inhibiting their translation.

In our study, *DICER1 *was down-regulated in TGCT cells after cisplatin exposure (Figure 5). This may explain the observed up-regulation of some of the hsa-mir-372/373 predicted target genes in TGCT cells (Table 2). Several reports have suggested that loss of Dicer1 causes embryonic lethality and loss of stem cell population [53]. Other reports suggest that Dicer1 participates in multiple biological processes, ranging from stem cell differentiation to maintenance of centromeric heterochromatin structure [54]. Cisplatin-induced down-regulation of *DICER1 *may repress the oncogenic properties of hsa-mir-372/373. It has been reported that these miRNAs regulate pathways controlled by classic tumor suppressors and oncogenes including p53, MYC and RAS [36,55].

Many p53 target genes were up-regulated in TGCT cells following cisplatin exposure in this study. However, the functionality of specific parts of the p53-dependent pathway may be suppressed through miRNA repression of some of the p53 target genes, such as *NEO1 *and *LATS2*.

Induction of apoptosis after exposure to cisplatin suggests that TGCT cells may already be primed to undergo programmed cell death [56]. Many genes in the apoptosis signaling pathway that take part in both the mitochondrial (intrinsic) pathway and the death receptor (extrinsic) pathway were engaged. Several apoptosis-related genes were affected in TGCT cells following cisplatin exposure (Table 3). The sensitivity of normal testicular cells to apoptotic stimuli [57] suggests that the unique responsiveness of TGCTs to cisplatin-based chemotherapy may represent inherent biological characteristics of normal germ cells and TGCT cells.

The non-caspase proteases, Calpain 1 (*CAPN1*) and Calpain 2 (*CAPN2*) take part in caspase-independent cell death pathway; they were up-regulated in TGCT cells after cisplatin exposure. Molecules involved in alternative forms of cell death have not yet been exactly defined, and non-caspase proteases such as calpains have also been taken into consideration as important for the response to cytotoxic therapy [58]. There might be a interaction between the mitochondrial, the death receptor and the caspase-independent cell death pathways. A cross-talk between these pathways may result in rapid and efficient induction of apoptosis in TGCT cells. Del Bello and co-workers recently reported that there is a cross-talk between calpain and caspase-3/-7 in cisplatin-induced apoptosis and that calpain activation is an early event in apoptosis [59].

Chemotherapy can induce a variety of anti-proliferative responses, including terminal growth arrest through senescence and cell death by mitotic catastrophe. Several cell lines treated with DNA-damaging agents have shown features of senescence, including the expression of senescence marker SA-β-gal (senescence-associated β-galactosidase). Other studies have identified a number of genes whose expression was increased or decreased in tumor cells during development of a senescent phenotype after treatment with DNA-damaging agents [43,60,61]. Among different classes of anticancer agents, the senescent phenotype is induced most strongly by DNA-damaging agents such as doxorubicin, aphidicolin, cisplatin, etoposide and ionizing radiation [62,63].

TGCT cells treated with low doses of cisplatin may undergo growth arrest, showing features of differentiation. The cisplatin doses used in our study may induce senescence-like growth arrest in TGCT cells. We observed significant enrichment of genes belonging to functional categories and biochemical pathways associated with differentiation, such as motility, Alzheimer disease pathway, and integrin signalling pathway. Chang and co-workers reported that genes induced in senescent cells were involved in cell adhesion, cell-cell contact, integrin signaling pathway, and Alzheimer disease pathway [44]. Senescent cells show permanent growth arrest and express markers associated with terminal differentiation. Consistent with this phenotype, we observed cisplatin induction of 6 testis-specific genes, *SPAM1, SPATA22, TCAM1, SPAG17, SOX17 *and *SOX7 *(Table 4). Other senescence associated genes such as *EDG1, LOC440895, MYADML, DDEF1 *and *DDEF2 *were also up-regulated in TGCT cells. The growth arrest that characterizes cellular senescence-like phenotype may be induced by lower doses of drugs or differentiating agents, and induction of cell death is generally associated with higher drug doses. Tumor cell lines were reported to express senescence-specific genes and to undergo terminal growth arrest after treatment with anticancer agents that affect DNA structure [62-65]. Several reports have suggested the cyclin-dependent kinase inhibitor (p21) and genes downstream from p53 to mediate the growth arrest observed at senescence in non-transformed cells. However, the level of p21 in cisplatin exposed TGCT cells is very low [66]. Other genes, including the Cdk inhibitor p16INK4a, are involved in the maintenance of senescence [43,67-69]. In addition to senescence and apoptosis, another major anti-proliferative effect of DNA-damaging agents is cell death through mitotic catastrophe [70]. The mutual effects of apoptosis, senescence-like phenotype, and mitotic catastrophe in tumors in response to treatment are illustrated by studies in which inhibition of apoptosis was shown to increase mitotic catastrophe, senescence, or both [71-73]. Studies also suggest that members of the insulin-like growth factor, IGF2 and its IGF-binding proteins (e.g., IGFBP5), constitute another senescence pathway [43,69]. In our study, we observed an induction of *IGFBP6 *and *IGFBP7 *in TGCT cells after cisplatin exposure (Table 4). From literature mining, we found 29 senescence-related genes highly expressed in TGCT cells following cisplatin exposure (Table 4). This comparison of our SAM-identified genes with senescence associated genes in the literature suggests the presence of senescence-specific genes which are conserved among cells derived form different tissues. Therefore, senescence-specific genes appear to be useful in the identification of cells displaying the terminal arrest and morphology associated with senescence. Knowledge about patterns of gene expression in senescent cells may lead to more effective treatment regimes for cancer and the development of new anticancer drugs.

The results of real-time PCR confirmed in general the gene expression results obtained by microarray analysis for the 8 selected genes (Figure 5). There were slight variations with respect to the magnitude of the relative expression, but the relative expression of the two methods was consistently in the same direction. The independent quantitative real-time PCR analysis further verified the validity of microarray results. Moreover, the real-time PCR measurements provide further support to the claim that the relative signal intensity from a microarray is a good quantitative measure of gene expression.

## Conclusion

This study has shown alterations of gene expression in TGCT cells treated with cisplatin. The systematic approach used proved to be valuable for identifying hundreds of alterations in gene expression, allowing identification of new functional classes and pathways which have not previously been shown to be associated with cisplatin-induced cellular response in TGCT cells. By linking our gene expression data to other publicly available microarray data, we have further identified gene expression patterns for cisplatin-exposed TGCT cells that are similar to the patterns reported for RA-induced differentiated TGCT cells. We have identified genes that take part in apoptosis signaling and terminal growth arrest. Cisplatin-induced genes associated with terminal growth arrest or senescent-like arrest were identified, indicating associations which have not previously been proposed for TGCT cells. We have also identified several p53 responsive genes and microRNA target genes that may play important functional roles. The differentially expressed genes are expected to contribute to a better understanding of the unusual sensitivity of testicular germ cell tumor cells to some DNA-damaging agents.

## Methods

### Cell culture and treatment

The human testis cancer cell lines 833K and GCT27 were generous gifts from Professor John R. W. Masters (University College London, UK). The 833K and GCT27 cells are derived from human metastatic and primary embryonal carcinoma, respectively [5,6] and a human colon cancer cell line, HCT116 (ATCC-CCL-247, Manassas, VA).

The 833K and GCT27 cells were grown in cell culture flasks (Corning Inc., USA) in RPMI 1640 medium with L-glutamine (Cambrex, Belgium) supplemented with 10% fetal bovine serum and 1% penicillin -streptomycin. The HCT116 cells were maintained in McCoy's 5A medium with L-glutamine (Cambrex, Belgium) supplemented with 10% fetal calf serum and 1% penicillin-streptomycin. All three cell lines were grown at 37°C in a humidified atmosphere of 5% of CO_2 _in air.

Cells were seeded in 100 mm cell culture dishes (Corning Inc, USA) and allowed to attach 24 h prior to cisplatin (Cis-Diamminedichloroplatinum(II) or CDDP, Sigma) treatment. Cells in exponential growth phase were exposed to cisplatin and harvested after 24 h and 48 h of exposure and prepared for further analysis.

### Cytotoxicity assay

Cells were exposed to cisplatin and their viability was determined 24 h and 48 h after treatment with different concentrations of cisplatin, by trypan blue exclusion and flow cytometry (Argus 100, Skatron, Norway) at the time RNA was harvested.

### Gene expression measurements

#### RNA preparation

Total RNA was isolated from cisplatin treated and control cell lines by GenElute kit (Sigma). RNA isolated from three individual treatments was mixed. The quantity and quality of isolated RNA was determined using a NanoDrop Spectrophotometer. RNA integrity was determined by an Agilent 2100 Bioanalyzer (Agilent Technologies, Palo Alto, CA). RNA from at least three independent experiments was pooled before labeling and hybridization to reduce possible bias of a single treatment.

### Microarrays

21K human oligonucleotide microarrays were printed at the Norwegian Microarray Consortium using OPERON hum oligo v2.0. The microarrays consist of 70 mer, oligos representing 21,329 genes from UniGene Database [74].

### cDNA synthesis and labeling

Labeled cDNAs were synthesized according to the manufacturer's protocol, FairPlay cDNA labeling kit (Stratagene, La Jolla, CA). Briefly, 20 μg of total RNA (from cisplatin treated or untreated control cell lines) in 13 μl of nuclease-free water was combined with 1 μl of 500 ng/μl oligo-d(T)_12–18_. The mixture was incubated at 70°C for 10 min and cooled on ice. For each reaction, 2 μl of 10× StrataScript reaction buffer, 1 μl of unlabeled 20× dNTP mix containing amino-allyl dUTP, 1.5 μl of 0.1 M dithiothreitol and 0.5 μl of RNase Block (40 U/μl) were prepared and mixed with the RNA sample and 1 μl of 50 U/μl StrataScript RT. After incubation at 48°C for 30 min an additional 1 μl of StrataScript RT was added and incubation was continued for 30 additional minutes. RNA was degraded by adding 10 μl of 1 M NaOH, followed by a 10-min incubation at 70°C, and the mixture neutralized with 10 μl of 1 M HCl. Unincorporated nucleotides were removed by precipitation of the cDNA with 4 μl of 3 M sodium acetate, 1 μl of 20 mg/ml glycogen and 100 μl of 95% ethanol at -20°C for 1 h. After centrifugation and washing the pellet with 70% ethanol, it was resuspended in 5 μl of 2 × coupling buffer provided in the kit. Cy3 (untreated sample) or Cy5 (cisplatin treated sample) dye (Amersham), resuspended in 45 μl of DMSO, was added and the reaction incubated for 2 h at room temperature in the dark. Dye-coupled cDNA was purified with a DNA-binding spin cup, combined, and concentrated by vacuum drying.

### Microarray hybridization and signal detection

Microarray slides were prehybridized in buffer containing 5× SSC, 1% Bovine Serum Albumin and 0.1% SDS at 42°C for 1 h to block nonspecific binding, and washed by dipping several times in double distilled water. The slides were then dipped in isopropanol and dried by centrifugation in 50 ml un-capped centrifuge tubes at 480 × g for 5 min.

Labeled cDNAs (combined Cy3 and Cy5 labeled cDNA) were added to hybridization buffer (50% formamide, 5 × SSC, 0.1% SDS, 4 μg/μl yeast tRNA, and 0.6 μg/μl human Cot-1 DNA), denatured for 5 min at 95°C, and applied to the arrays underneath a cover slip (LifterSlip, Erie Scientific). Arrays were placed in a hybridization chamber and incubated overnight in a water bath at 42°C.

After hybridization, washing was performed as follows: 5 min washing with 2 × SSC, 0.1% SDS at 42°C, 10 min washing with 0.1 × SSC, 0.1% SDS and 4 × 1 min washing with 0.1 × SSC at room temperature. Slides were dried by centrifugation at 480 × g for 5 min and scanned with an Agilent microarray laser Scanner (Agilent, Palo Alto, CA).

### Data analysis

In this study we have used time-matched pooled RNA from untreated samples as a reference RNA. RNA from cisplatin treated samples was always co-hybridized with the reference RNA from the same cell line. Gene expression levels are hence always relative to the untreated sample. The microarray images were analyzed using the GenePix 4.1 image analysis software (Axon Instruments). This included defining the spots, intensity calculation, flagging spots with inadequate measurements, and subtracting local background. Flagged spots were filtered out and normalized using LOWESS normalization. All data filtering and normalization was performed in J-Express Pro v 2.7 (MolMine, Bergen, Norway) [75]. Only those features that passed quality assurance criteria were included in the down-stream analysis. Missing values were estimated by the k-nearest neighbor imputation (k = 15). The ratios of the processed intensities of all genes and samples were divided by the median of the ratios of three untreated samples before log2 transformation. This was done to facilitate the interpretation of the expression values. Replicate data were averaged and used in all clustering. The data generated from the arrays were deposited in NCBI Gene Expression Omnibus (GEO), with following GEO Series accession number: GSE7563 [76], according to the Minimum Information About a Microarray Experiment (MIAME) recommendations [77].

### Identification of genes of interest

Genes with statistically significant changes in expression before and after cisplatin treatment were identified using SAM (Significance Analysis of Microarrays). SAM selects significant genes based on differential expression between sets of samples [45]. SAM gives estimates of the False Discovery Rate (FDR), which is the proportion of genes likely to have been wrongly identified by chance as being significant. The effect of multiple comparisons is controlled through the FDR. It allows dynamically changing thresholds for significance, through adjusting a threshold delta-value. Different stringencies can be obtained by adjusting the threshold delta, resulting in different numbers of genes whose expression is classified as significantly changed. We have used two-class, unpaired SAM analysis to select genes whose mean expression level is significantly differentially expressed between TGCT and HCT116 cell lines.

### Annotation and clustering

The genes (oligo probes) that were identified as statistically significant with SAM were further annotated using the Norwegian Microarray Consortium Datawarehouse (Unigene build # 197, Dec 01 2006) [26,27] and analyzed for functional gene clusters by eGOn v2.0 [26,27] and Panther [24,28]. The eGOn v2.0 uses Fisher's exact test to determine whether, in any of the GO categories, the genes of interest are over- or underrepresented compared to the genes represented on the microarray [27]. Panther uses the binomial statistics tool to determine over- or under- representation of Panther classification categories. Panther determines functional clusters by representation of individual genes in specific categories relative to all genes in the same category on the array [24,28]. All hierarchical clustering analysis and Principal Component Analysis (PCA) were done by J-Express Pro v 2.7 [75]. PCA reduces the dimensionality and classifies variables [78]. Hierarchical clustering analysis (average-linkage and Euclidean distance similarity measurement) was used to cluster variables into groups based on their similarity, and the results were visualized in a dendrogram.

### Real time polymerase chain reaction (RT-PCR)

Validation of the expression of candidate genes identified by microarray analysis was carried out by quantitative real-time PCR using SYBR green chemistry. cDNA synthesis was conducted using ABI High Capacity Archive Kit (Applied Biosystems, Foster City, CA) from the same RNA samples as that used in the microarray experiments. Real-time PCR was performed in 96 well PCR plates using the Applied Biosystems Power SYBR^® ^GREEN PCR master mix and the 7500 Fast Real-Time PCR System (Applied Biosystems, Foster City, CA, USA) according to the manufacturer's protocol; three independent measurements were made for each gene of interest for each cDNA. The primer sequences used to validate the microarray data are listed at PrimerBank [79]. Thermo-cycling parameters were as follows: 95°C for 15 min; then 40 cycles of 95°C for 15 sec, 60°C for 60 sec, and 72°C for 35 sec to measure the fluorescence signal; followed by the dissociation stage. Melting curves and quantitative analysis of the data, and the average cycle threshold (Ct) measurement for the three determinations, were used in calculations of relative expression using 18S rRNA as the endogenous control in the ΔΔCt method (7500 System SDS software v1.3; Applied Biosystems). The obtained fold change results were log2-transformed. All reagents used in the real-time PCR were purchased from Applied Biosystems.

## Competing interests

The author(s) declare that they have no competing interests.

## Authors' contributions

ND carried out the microarray experimental work, participated in the study design, performed the statistical analyses, interpreted the results, performed RT-PCR experimental validation, drafted the manuscript and prepared the finale version of the manuscript. BL carried out cell culture treatments, cell viability assessments, participated in the study design and participated in scientific discussions and manuscript preparation. MK participated in the microarray experimental work. ÅA participated in cell culture and in scientific discussions. AO and ES participated in scientific discussions and manuscript preparation. GB participated in the study design, in scientific discussions and manuscript preparation and supervised the work. All authors have read and approved the final version of the manuscript.

## Supplementary Material

Additional file 1 Complete list of SAM-identified genes with gene name and SAM score. SAM analysis comparing TGCT versus HCT116 cell lines following cisplatin exposure. The positive significant genes (n = 1180, red) are over-expressed in TGCT cells and under-expressed in HCT116 cells. The negative significant genes (n = 614, green) are under-expressed in TGCT cells and over-expressed in HCT116 cells. The complete list of SAM-identified genes is available as supporting information. Click here for file

Additional file 2 Functional classification of SAM identified genes. Complete list of functional categories of the 1180 up-regulated and 614 down-regulated genes in TGCT cell lines following cisplatin exposure. Click here for file
