# Supplementary material for: Molecular portrait of cisplatin induced response in human testis cancer cell lines based on gene expression profiles
Source: Mol Cancer. 2007 Aug 21;6:53. doi: 10.1186/1476-4598-6-53 (PMC1988831; doi:10.1186/1476-4598-6-53)

Additional file 1: Significance Analysis of Microarrays (SAM) comparing TGCT cell lines versus HCT116 cell lines following cisplatin exposure. The positive significant genes (n=1180, red) are most highly expressed in TGCT cells, whereas the negative significant genes (n=614, green) are most highly expressed in HCT116 cells.

Current settings

Input parameters

**Data type?**	Two class unpaired
**Arrays centered?**	SANN
**Delta**	3,833
**Minimum fold change**	2
**Test statistic**	standard
**Are data are log scale?**	SANN
**Number of permutations**	300
**Input percentile for exchangeability factor s0**	Automatic choice
**Number of neighbors for KNN**	10
**Seed for Random number generator**	1234567

Computed values

**Estimate of pi0 (proportion of null genes)**	0,217329187
**Exchangibility factor s0**	0,090835634
**s0 percentile**	5,000420203
**False Discovery Rate (%)**	0

List of Significant Genes for Delta = 3,833

Positive genes (1180)

Row	Gene ID	Gene Name	Score(d)	Numerator(r)	Denominator(s+s0)	Fold Change	q-value(%)
393	H200000451	IL1A	8,62139647	2,98623989	0,34637543	7,65955067	0
10320	H200016886	EXOC6	8,35666073	4,06656637	0,48662576	19,04075871	0
9563	H200015045	FYN	8,22642512	4,46694508	0,54299954	24,36684315	0
5433	H200007237	ANGEL2	8,12650564	1,81566189	0,22342468	3,51381061	0
10679	H200017770	BCL2L10	8,02743954	4,33229868	0,53968624	21,88783387	0
10712	H200017833	ASCL3	7,98473220	3,08388191	0,38622233	8,81467983	0
8232	H200012226	PCDHB5	7,91814462	3,77319780	0,47652550	12,80515004	0
6945	H200010040	LOC283075	7,85855289	5,22327716	0,66466145	33,81514721	0
10866	H200018251	ROCK1	7,83219174	4,51109817	0,57596881	22,14185962	0
10868	H200018257	USP6NL	7,80222454	2,62475216	0,33641074	5,99329996	0
11200	H200019213	GPC5	7,79792648	3,96120400	0,50798171	17,06471773	0
10924	H200018507	NF1	7,76612689	4,05201245	0,52175460	16,80132190	0
10911	H200018441	PIK3C3	7,76392043	4,05633717	0,52245991	17,73388510	0
8431	H200012718	HDGFRP3	7,71835198	2,86996055	0,37183592	7,32654443	0
10915	H200018453	VPS54	7,70773862	4,39217989	0,56984027	21,57955167	0
7139	H200010383	CXCL5	7,70547588	3,69052190	0,47894795	12,42466407	0
11284	H200019433	FANCF	7,64798451	4,24088489	0,55451013	18,82838098	0
9635	H200015192	SLC25A36	7,60620096	1,94589462	0,25583003	3,80433817	0
8589	H200013085	LOC730245	7,60562374	4,54066348	0,59701395	21,56735421	0
10869	H200018262	NAV3	7,60357987	3,93242668	0,51718095	14,93081504	0
9740	H200015431	PCDHB9	7,59725985	3,66289543	0,48213376	12,61150220	0
10833	H200018186	PRDM5	7,59306860	3,85580601	0,50780603	14,46559691	0
10559	H200017495	PCNXL2	7,57446409	4,40699444	0,58182261	20,42558634	0
8425	H200012700	DNAH5	7,57093380	4,28320055	0,56574270	20,75923446	0
11248	H200019349	FTHL17	7,56644343	4,36069802	0,57632071	19,99203391	0
10884	H200018324	SLC39A3	7,54054726	3,13887289	0,41626593	8,84109165	0
10990	H200018768	ZNF33A	7,46737802	3,24214968	0,43417511	9,63485952	0
1125	H200001343	TMEM55B	7,44373914	3,95900683	0,53185728	16,24584612	0
8376	H200012576	KERA	7,40548783	5,04824581	0,68168984	25,32584878	0
9370	H200014639	CLCA1	7,37410182	2,00584456	0,27201205	3,96736479	0
8845	H200013701	ZFP37	7,33307229	4,04206958	0,55121093	15,57528282	0
7807	H200011536	CLSTN3	7,31392260	3,01271485	0,41191506	7,68508615	0
10640	H200017622	FLJ13611	7,26907573	3,27616968	0,45069962	11,32961233	0
11826	H200021171	LOC254100	7,26081174	3,71954604	0,51227689	14,17677361	0
11287	H200019440	MAML2	7,25838631	3,81307262	0,52533338	13,99887533	0
10571	H200017515	PRKCA	7,22974942	3,15716813	0,43669122	9,29189965	0
10306	H200016786	DMXL2	7,21023568	3,96222959	0,54952844	14,59730406	0
10809	H200018136	DEPDC1B	7,19600699	3,68775098	0,51247185	12,76104316	0
10392	H200017105	OR12D3	7,19567219	4,62216375	0,64235330	22,12907991	0
2946	H200003793	ZNF488	7,19238673	3,17361264	0,44124611	8,71168703	0
10129	H200016423	SLC30A6	7,18468897	4,91813398	0,68452984	24,69298915	0
2605	H200003326	CHFR	7,17393312	2,83428679	0,39508130	6,38636913	0
6473	H200008941	MEGF10	7,16479623	3,76077210	0,52489589	13,77831372	0
8578	H200013051	PCDH20	7,14399206	4,63414870	0,64867775	20,82750540	0
10795	H200018097	NRIP1	7,14023776	4,42379568	0,61955860	25,40208636	0
10785	H200018031	LOC130951	7,13719863	2,99421676	0,41952269	7,57039940	0
11106	H200019053	SLC30A6	7,11128320	2,44676407	0,34406787	5,46588925	0
11428	H200019841	PDE3B	7,09185045	4,18881555	0,59065199	19,10275328	0

8744	H200013452	PIP5K2C	7,07144778	2,39514141	0,33870595	5,05054362	0
10742	H200017925	USP3	7,06738961	3,54130548	0,50107687	11,85164833	0
6666	H200009436	POM121	7,05830185	2,31148041	0,32748393	4,63241589	0
11189	H200019178	AKT2	7,03466729	3,10356331	0,44118125	8,82652181	0
11322	H200019516	PMS2L1	7,03293686	3,37909432	0,48046703	11,51304634	0
3101	H200004001	SENP7	7,02577838	3,74436166	0,53294617	13,58327847	0
9411	H200014717	C14orf118	7,02458407	2,54429458	0,36219861	5,67360027	0
7797	H200011524	DDIT4L	7,00946798	3,68090465	0,52513324	12,18030618	0
2555	H200003246	VGLL3	6,99883326	3,49545728	0,49943428	11,26757818	0
11183	H200019166	LOC92482	6,99541982	3,69661036	0,52843295	14,90055328	0
9221	H200014303	PCMTD2	6,99364500	2,55279927	0,36501699	5,81474069	0
484	H200000566	CSN2	6,98686784	3,22394282	0,46142891	9,97621782	0
8821	H200013651	ATPBD4	6,98517657	3,42963244	0,49098722	10,26498810	0
7765	H200011475	RP11-35N6.1	6,98131821	4,82880949	0,69167589	24,42534830	0
8545	H200012984	RPL24	6,96875598	3,04668736	0,43719243	7,79873682	0
3111	H200004015	ZNF451	6,95062908	3,36909544	0,48471806	10,06139593	0
8032	H200011892	SH3MD4	6,93403757	3,65207098	0,52668751	13,47731844	0
7228	H200010523	WNT10B	6,92923526	1,89480069	0,27345019	3,72544351	0
10885	H200018326	IBTK	6,91423499	4,76531419	0,68920339	22,07313134	0
11233	H200019326	BTBD10	6,88657542	2,45353518	0,35627798	5,20640502	0
11719	H200020780	C17orf77	6,87215102	4,43228239	0,64496289	19,71142175	0
10982	H200018722	KRTAP4-14	6,87079327	4,01150558	0,58384897	15,95092023	0
11320	H200019513	PSG11	6,86778773	3,59414718	0,52333405	12,59786024	0
10902	H200018381	DKFZp667M2	6,86350091	4,27899148	0,62344153	17,60153116	0
8428	H200012710	DLL3	6,86278695	2,10247219	0,30635837	4,21249812	0
10737	H200017913	FAM91A1	6,84624209	3,21330536	0,46935316	9,56182685	0
11318	H200019509	BEX1	6,84254234	3,32068824	0,48530036	9,69962381	0
8745	H200013453	LOC727846	6,82598572	3,07345785	0,45025846	8,44216603	0
11067	H200018986	JMJD1A	6,81965651	2,62153174	0,38440818	6,15307610	0
11806	H200021089	TMEM25	6,81820119	3,13197268	0,45935469	9,51217202	0
1034	H200001225	C14orf132	6,80588498	2,89567344	0,42546611	7,44386716	0
11570	H200020254	SYNC1	6,80186892	3,44942803	0,50712945	11,24464886	0
10714	H200017855	ATBF1	6,78036933	2,99910704	0,44232208	8,28476517	0
9311	H200014481	SNX12	6,77272609	3,38514980	0,49982086	11,08084099	0
10429	H200017217	KCNMB3	6,76160521	2,72214528	0,40258862	6,29941349	0
10873	H200018274	UBE2D3	6,75485018	3,16236601	0,46816227	8,73885213	0
11771	H200020995	SKP1A	6,72633045	2,54862496	0,37890273	6,52028388	0
11739	H200020866	TFE3	6,72115666	4,11580497	0,61236558	16,17294292	0
9105	H200014096	FOXF1	6,72011862	4,15387550	0,61812532	16,16939084	0
10980	H200018719	KRTAP4-14	6,70636654	4,09208487	0,61017913	15,90353685	0
10336	H200016939	SYNE1	6,70454534	2,43149485	0,36266364	5,39768644	0
10175	H200016522	PLGLB2	6,69291189	3,60082993	0,53800647	13,18347687	0
923	H200001085	PSMD9	6,68699734	1,87408744	0,28025844	3,49131472	0
6832	H200009778	DEPDC6	6,67993687	3,70793318	0,55508506	13,44319911	0
11052	H200018945	ZF	6,67600951	3,16423099	0,47397041	9,34646796	0
7586	H200011161	STX16	6,67227779	2,38456187	0,35738348	5,31199660	0
10976	H200018687	DCK	6,65468998	2,51137393	0,37738406	5,90114119	0
8027	H200011876	TFEC	6,63658592	4,26731310	0,64299825	16,61291434	0
8249	H200012269	PIK3CA	6,62976818	3,44644371	0,51984377	10,95133068	0
8410	H200012669	AGMAT	6,62920557	4,07947882	0,61537974	16,89689486	0
9284	H200014424	LOC440896	6,60326258	3,48109489	0,52717802	10,91718600	0
7465	H200010966	LOC284019	6,60322341	1,57051837	0,23784117	2,93484440	0
10900	H200018379	DCAMKL2	6,59592206	3,08190578	0,46724412	8,36705029	0
10402	H200017141	LOC651959	6,58242268	2,78428280	0,42298754	7,12013270	0
9533	H200014966	TEAD3	6,58178595	1,97795086	0,30051887	4,16071034	0
10455	H200017260	CRKRS	6,57549370	3,49822255	0,53200911	11,15679716	0
7159	H200010412	NCALD	6,56188878	3,36249222	0,51242749	10,49356513	0
9556	H200015019	SC65	6,55882133	3,31946709	0,50610726	9,79475446	0
11325	H200019523	GHRL	6,55226030	3,00853004	0,45915912	8,41177476	0
8388	H200012599	GSTCD	6,53789655	4,04367947	0,61849854	13,84278063	0
2927	H200003768	ZNF529	6,53296483	3,90850681	0,59827458	13,77488306	0
8825	H200013662	KIAA1217	6,52792603	2,24441687	0,34381775	4,54687043	0
3745	H200004941	LPXN	6,52574522	2,60946020	0,39987160	6,05804786	0
1551	H200001895	BCL2L13	6,51259680	1,50890252	0,23168984	2,86176674	0
9301	H200014461	PABPC5	6,50463699	4,43415608	0,68169155	43,58035143	0
11500	H200020047	FBXO10	6,50295673	3,24431510	0,49889846	9,40093076	0
5875	H200008009	RREB1	6,49250433	2,50897177	0,38644129	5,86519771	0
6122	H200008357	COL1A2	6,48119675	2,28118175	0,35196922	4,77648005	0
10475	H200017301	PPP3R1	6,47961289	1,88933547	0,29158153	3,84434161	0
11408	H200019779	PCNXL2	6,47440432	2,31736103	0,35792652	4,64413261	0
11327	H200019526	USP48	6,46159037	2,13412941	0,33027928	4,39216565	0
1066	H200001262	AXUD1	6,45803458	1,38456159	0,21439365	2,61782318	0
5612	H200007598	FCRLB	6,45549667	3,45983562	0,53595189	11,04597778	0
6697	H200009493	CYB5R1	6,45111419	2,80569075	0,43491568	6,81814648	0
11444	H200019893	TM6SF1	6,43966504	2,22251417	0,34512885	4,76195984	0
7330	H200010677	C1QC	6,43785230	3,73375230	0,57996862	11,41485219	0
8099	H200012021	HDAC9	6,43772586	2,88856406	0,44869324	7,39148158	0
5661	H200007684	LCORL	6,42353645	3,20493715	0,49893656	8,69190899	0
8392	H200012617	TTLL2	6,39967848	2,01055650	0,31416524	3,95834927	0
10333	H200016919	PKD2L2	6,39407283	2,92464067	0,45739871	8,20560509	0
11278	H200019415	SLC6A16	6,37983748	2,86412638	0,44893407	7,12294479	0
10987	H200018738	PHGDHL1	6,37731099	3,03837347	0,47643489	8,43394594	0
4170	H200005576	SFXN5	6,36518264	2,75645833	0,43305251	6,23375103	0
6937	H200010024	TMTC2	6,36055382	4,74026692	0,74526009	21,25279650	0
7408	H200010846	SOX17	6,35518478	2,87316841	0,45209833	7,60169275	0
9781	H200015528	LOC440248	6,35421702	2,89514561	0,45562586	7,66235042	0
10962	H200018604	NTN4	6,35209205	2,47733171	0,39000249	5,38731871	0
11802	H200021081	TPM1	6,34781590	2,91639628	0,45943303	7,72392731	0
7301	H200010632	EYA1	6,33922197	2,19216349	0,34580955	4,62044481	0
10648	H200017633	ADAM30	6,33811555	2,99829976	0,47305855	8,55787384	0
7597	H200011180	FNTA	6,33354242	3,08921629	0,48775489	7,90673515	0

8533	H200012964	TNKS	6,31892530	2,25449039	0,35678383	4,68710130	0
10927	H200018522	THADA	6,31237376	3,47034244	0,54976821	11,02372405	0
80	H200000090	IL10RA	6,30788096	3,23971362	0,51359777	10,59073829	0
10936	H200018553	PTK2	6,29889289	3,38271871	0,53703385	10,46260039	0
10228	H200016607	B3GALNT1	6,26383268	2,76283467	0,44107734	6,99478970	0
9939	H200015829	EML4	6,26221802	2,65162661	0,42343250	6,54664354	0
6978	H200010107	APTX	6,25835033	3,59669843	0,57470391	11,94075353	0
10914	H200018451	GRIK2	6,25455622	3,60711670	0,57671825	12,52222117	0
4704	H200006327	HLA-DRA	6,24241237	3,02674899	0,48486848	8,17659619	0
151	H200000176	DCK	6,24132636	2,91505853	0,46705754	7,56937066	0
3663	H200004822	P15RS	6,23303091	1,96658393	0,31551006	3,73315590	0
7696	H200011360	SPATA22	6,23039125	2,74748726	0,44098150	6,24857556	0
4589	H200006192	RB1	6,21716263	2,52006629	0,40534026	5,98746047	0
10878	H200018311	RANBP10	6,21496552	3,09473392	0,49794869	8,59672348	0
6012	H200008177	IL1RAP	6,21106892	2,69661083	0,43416212	5,99745134	0
10847	H200018209	LENG8	6,19656840	2,76249541	0,44581053	6,83250596	0
4227	H200005670	RBBP9	6,19252457	2,01025600	0,32462625	3,93856801	0
6426	H200008852	AEBP2	6,18133558	2,61486870	0,42302649	7,26745891	0
4394	H200005939	ITGA7	6,17730646	4,12269070	0,66739294	19,21392617	0
10950	H200018580	SLC24A3	6,17475003	2,82556332	0,45759963	6,72863032	0
1101	H200001310	NETO2	6,17338925	2,86509720	0,46410441	6,86135072	0
11705	H200020719	GUP1	6,17249806	3,41063875	0,55255404	10,65659376	0
10688	H200017782	ALS2CR13	6,16900591	3,09802877	0,50219254	8,71452613	0
10864	H200018246	PSPC1	6,16725963	3,57192010	0,57917460	11,19388050	0
1089	H200001293	C15orf38	6,16672722	3,99278373	0,64747209	11,53531589	0
8044	H200011916	MGC16385	6,16627208	1,81740197	0,29473269	3,45277321	0
10908	H200018431	RUFY3	6,15494748	3,51677409	0,57137353	13,32154046	0
2303	H200002911	SAP30L	6,14992636	3,76133867	0,61160711	11,05019877	0
7373	H200010748	ADAMTSL4	6,12730293	3,10694292	0,50706534	7,71796108	0
11870	H200021279	NFXL1	6,12647885	1,96026736	0,31996640	4,09339117	0
10942	H200018563	MKL1	6,11599665	2,47073566	0,40397924	5,49454879	0
11037	H200018907	TRIM22	6,11217822	2,75892789	0,45138211	6,96497555	0
10968	H200018632	KCNJ15	6,10526489	2,97080211	0,48659676	8,06352562	0
11773	H200021004	FLJ36166	6,10464391	2,96993238	0,48650379	8,17534696	0
901	H200001060	EXDL2	6,09685708	1,81344167	0,29743877	3,51080575	0
24	H200000023	ABHD14B	6,09098726	2,72950927	0,44812264	6,88903926	0
11285	H200019434	OPA1	6,08973909	4,06928851	0,66822050	14,27278000	0
9014	H200013962	EDEM1	6,08165526	2,03662566	0,33488016	3,96328547	0
10895	H200018360	ZNF559	6,08046915	4,02344161	0,66169921	14,91903463	0
3309	H200004309	HERC3	6,07812183	2,10219385	0,34586241	4,12378036	0
4090	H200005466	PCDH17	6,07660329	1,92276378	0,31642082	3,82719710	0
2156	H200002714	EDIL3	6,07643167	2,92799340	0,48186066	7,34205245	0
2877	H200003697	EVI5L	6,07591891	2,57499347	0,42380313	6,10558795	0
8372	H200012570	C1orf58	6,06191850	2,98364411	0,49219469	7,83282823	0
10294	H200016758	KLF12	6,05604349	3,29854539	0,54467003	9,58235522	0
9651	H200015236	L2HGDH	6,05139124	2,81525719	0,46522478	7,18390511	0
10910	H200018434	SMYD2	6,04679673	2,62474681	0,43407227	6,56421069	0
8104	H200012033	ZNF264	6,03581912	2,33580977	0,38699135	5,11194509	0
8583	H200013067	ZNF643	6,03550695	3,23814547	0,53651590	9,47731340	0
11205	H200019228	C1QTNF5	6,03510094	3,04051565	0,50380527	8,76727437	0
11077	H200019004	C1orf61	6,02124691	3,03241508	0,50361912	8,17032275	0
6037	H200008209	NAV3	6,01579618	1,56671828	0,26043407	2,89303012	0
11269	H200019399	C8orf72	6,00568844	2,43306679	0,40512704	5,10546915	0
5479	H200007308	ZNF230	6,00335118	2,80687571	0,46755148	7,81608283	0
11818	H200021128	FLJ21127	6,00261888	1,92894194	0,32135006	3,89563524	0
10970	H200018650	TCF7L2	6,00117582	2,92099958	0,48673788	7,57093140	0
6021	H200008189	EIF3S1	5,98978488	2,30803626	0,38532874	4,74565600	0
10416	H200017191	SLCO1B3	5,98628381	3,02802302	0,50582684	8,65298723	0
1126	H200001344	ERC2	5,98379104	3,10686032	0,51921270	9,07017912	0
10137	H200016442	MAPK14	5,97538707	3,85178768	0,64460890	11,91396995	0
7282	H200010598	LOC440895	5,97521373	2,93336298	0,49092185	7,92670958	0
7126	H200010366	PTPRG	5,97513464	2,67074377	0,44697633	5,87559389	0
8689	H200013335	LOC284561	5,97167153	1,92616876	0,32255102	3,92156884	0
8463	H200012802	LOC728190	5,97039228	2,33312596	0,39078269	5,06983228	0
10684	H200017778	FHL5	5,96603933	2,77943781	0,46587655	7,34232979	0
407	H200000469	MPO	5,96478454	2,20487350	0,36964847	4,91292399	0
10149	H200016468	PTPRO	5,96042809	2,48971252	0,41770700	5,66323527	0
8415	H200012675	ALG11	5,96004665	2,53588221	0,42548026	5,80887902	0
4472	H200006031	PKIA	5,95952242	3,20147277	0,53720291	8,74229779	0
10314	H200016838	OR12D3	5,95546622	2,55698259	0,42935053	6,33197074	0
8505	H200012902	KIAA1542	5,95414113	3,16646873	0,53180949	9,11303368	0
8625	H200013163	CCDC71	5,95332344	2,55903525	0,42984986	5,93118322	0
10786	H200018033	PLCH1	5,94681634	2,92346345	0,49160144	7,82281592	0
3264	H200004238	OGFOD2	5,94324443	2,22376585	0,37416698	4,49289283	0
10017	H200016169	CD1E	5,93997235	3,07864090	0,51829213	8,12179748	0
4285	H200005771	HSPA4L	5,93514333	2,53436597	0,42701007	6,52644740	0
6613	H200009348	TPCN2	5,93013682	2,02044186	0,34070746	4,19763367	0
9804	H200015564	DENND1C	5,92963072	2,69709469	0,45485036	6,22768402	0
11042	H200018917	PLEKHM1	5,92832819	3,08139280	0,51977433	8,31021703	0
1774	H200002195	TMEM128	5,91596431	2,58331099	0,43666778	5,56956740	0
10887	H200018337	LOC647135	5,91249258	2,99714931	0,50691807	8,47188669	0
4316	H200005827	GNRHR	5,90258444	2,99239201	0,50696302	7,82858791	0
358	H200000414	GSTT2	5,90234147	2,07212187	0,35106777	4,26860008	0
8337	H200012502	C6orf199	5,90094616	3,89208333	0,65956937	25,03855012	0
8948	H200013865	KIAA0701	5,89882579	3,32813340	0,56420269	9,48619331	0
10535	H200017455	TMEM38B	5,89346977	2,13596226	0,36242864	4,65117395	0
10814	H200018147	PKP4	5,89202290	2,95994697	0,50236515	7,95491313	0
8841	H200013689	PCBD2	5,88554005	2,90026370	0,49277784	7,91664837	0
11781	H200021026	DIAPH3	5,88187499	3,55341236	0,60412919	11,40106433	0
8614	H200013134	ABCC9	5,87452897	2,83623970	0,48280291	7,13502150	0
7124	H200010364	NRL	5,86722296	2,98769360	0,50921767	8,26571152	0

7397	H200010817	MTFR1	5,86699700	2,95733273	0,50406242	7,68108705	0
2822	H200003624	CCRK	5,86418118	3,08094362	0,52538343	8,43336938	0
11130	H200019089	APBA2BP	5,84643761	2,36875927	0,40516284	6,04790922	0
11704	H200020718	TPST2	5,84081845	3,38380284	0,57933710	12,02543489	0
9746	H200015443	PHC3	5,83785012	2,79719244	0,47914770	7,20695458	0
6464	H200008920	HRH4	5,83470382	3,28811830	0,56354502	9,80443925	0
1080	H200001282	SLC7A11	5,83251714	2,65688447	0,45552965	7,65720695	0
228	H200000271	REG1A	5,83123352	2,87401406	0,49286554	8,09758452	0
5709	H200007762	TAS2R1	5,82629004	3,21011325	0,55097038	9,02393634	0
5036	H200006723	STS	5,81496075	2,52534968	0,43428490	6,02025858	0
11730	H200020839	CAPN1	5,81342213	3,69119708	0,63494393	14,97774219	0
2500	H200003177	SCN1A	5,81295814	3,07166478	0,52841681	8,22146034	0
2538	H200003224	UGCGL2	5,81183423	2,30288509	0,39624067	5,19185732	0
9510	H200014904	ENTPD4	5,80797330	1,85725770	0,31977725	3,60126833	0
8553	H200012997	XPNPEP3	5,80450909	3,17049739	0,54621284	8,45295085	0
3870	H200005125	SCGB3A2	5,80353653	2,26155573	0,38968579	4,30813126	0
3093	H200003991	SLC19A2	5,80323921	2,75293379	0,47437882	6,52176140	0
7275	H200010590	ARHGAP24	5,79976300	3,06253499	0,52804485	8,57947773	0
1079	H200001281	HECA	5,79526770	1,74861495	0,30173152	3,63850594	0
9015	H200013963	VPS13A	5,78698043	3,13064763	0,54098120	9,30822641	0
10830	H200018176	CCL2	5,77507512	2,79236459	0,48352005	6,70322752	0
10758	H200017959	RFTN1	5,76944417	2,69690201	0,46744572	7,03645443	0
1241	H200001489	C16orf5	5,76776769	1,55907067	0,27030747	2,92246740	0
1162	H200001390	RBAK	5,76431770	1,58877531	0,27562244	3,02947288	0
11732	H200020846	C10orf112	5,76231426	1,86014532	0,32281220	3,61497874	0
5687	H200007733	STAT5A	5,76110220	2,69870200	0,46843502	5,92193490	0
10164	H200016499	MULK	5,75936781	1,96639950	0,34142628	3,86035156	0
11194	H200019193	C16orf70	5,75695163	2,00294208	0,34791713	4,19394972	0
8613	H200013133	C1GALT1C1	5,75671713	2,48193467	0,43113716	5,78944788	0
3024	H200003900	HPS5	5,75513741	2,06109240	0,35813087	4,09700432	0
11207	H200019238	FKSG83	5,75040528	2,66764453	0,46390548	6,10590951	0
8122	H200012072	COL17A1	5,74616645	2,00577120	0,34906249	4,00512205	0
10896	H200018365	IL1RAPL1	5,74494530	2,91671940	0,50770186	7,59371244	0
10630	H200017600	NUDT16	5,73830604	2,60203655	0,45345029	6,54115534	0
7683	H200011334	TMEM45A	5,73710347	2,01014129	0,35037564	3,96926266	0
5034	H200006721	KIAA1333	5,73652780	2,93523705	0,51167486	7,25746374	0
9222	H200014304	NOTCH1	5,73367761	1,66075211	0,28964867	3,11516371	0
2535	H200003221	KIAA1576	5,73340468	2,72262723	0,47487093	6,38091511	0
7544	H200011080	GOSR2	5,73300098	1,65631246	0,28890846	3,07856816	0
10983	H200018723	KRTAP4-8	5,73271658	4,15066714	0,72403146	31,19454854	0
11799	H200021070	RGS6	5,73006415	2,62285229	0,45773524	6,49032380	0
11281	H200019422	KIAA0408	5,73006333	2,74267498	0,47864654	7,17963816	0
8283	H200012363	C7orf31	5,72958415	2,01243245	0,35123534	4,14099131	0
561	H200000661	GLRA2	5,72776319	1,64924368	0,28793852	2,92732710	0
8411	H200012670	UHMK1	5,72053303	2,38528710	0,41696938	5,38670741	0
6086	H200008308	MFAP3L	5,72051329	3,23542639	0,56558323	8,51190430	0
8217	H200012206	HEXA	5,71412651	1,35608391	0,23732130	2,54997853	0
1100	H200001309	C10orf9	5,71089083	2,89976844	0,50776114	8,10843246	0
10917	H200018464	SBNO1	5,71030854	2,77934050	0,48672335	7,07226755	0
10860	H200018231	MPP5	5,69785000	2,66441345	0,46761734	6,37670300	0
11755	H200020955	SYMPK	5,68261260	4,18521761	0,73649532	69,83094760	0
10409	H200017154	RAB28	5,67318992	2,95273112	0,52047105	7,08465504	0
10711	H200017831	OR2S2	5,66733107	2,84018372	0,50115013	7,64878444	0
9662	H200015257	TAF7L	5,66595905	2,79791723	0,49381176	7,20533192	0
753	H200000882	FLJ40432	5,66334887	2,08243965	0,36770464	4,51324202	0
464	H200000540	EPS8	5,66110601	2,28239793	0,40317173	4,93059361	0
11364	H200019657	JAM3	5,65396321	1,76668205	0,31246791	3,38616440	0
11010	H200018845	ZNF3	5,64621422	2,67424871	0,47363572	6,69066925	0
670	H200000789	TMEM98	5,64552056	1,79619589	0,31816302	3,30224935	0
5660	H200007683	C1orf136	5,63914546	2,56447157	0,45476244	6,06686778	0
9274	H200014398	RPS6KA3	5,63467119	2,26050543	0,40117788	4,92027390	0
10916	H200018454	MARCH8	5,63081482	1,41658381	0,25157705	2,67923596	0
11289	H200019446	MYO1E	5,62667846	2,76172602	0,49082706	7,01796379	0
5802	H200007901	TTC33	5,62444650	3,18283564	0,56589313	8,96656248	0
11707	H200020727	REPS2	5,61859751	2,41353855	0,42956246	5,30596225	0
7577	H200011142	USP53	5,61678680	2,64999964	0,47179993	5,44939419	0
569	H200000672	HOXB2	5,61507496	3,23604443	0,57631366	9,67884678	0
2677	H200003425	PPCDC	5,60219781	2,84503497	0,50784265	7,16914098	0
7381	H200010771	LOC220930	5,60189345	2,25649593	0,40280951	4,44773000	0
5068	H200006762	F13A1	5,60127663	1,90155746	0,33948644	3,79084513	0
7341	H200010697	MYO7A	5,60102698	2,12549360	0,37948283	4,50688046	0
9213	H200014292	STK38L	5,59780113	2,04363377	0,36507795	3,88182358	0
4008	H200005329	GPR87	5,59402858	2,75661921	0,49277889	7,88877707	0
11761	H200020975	ZNF396	5,58714921	3,00963650	0,53867122	9,01114019	0
9494	H200014871	NUDT11	5,58652953	3,21151480	0,57486760	9,62655378	0
11674	H200020517	RFTN2	5,58450356	3,00710024	0,53847226	8,28929007	0
1828	H200002267	COG3	5,57740710	1,96252154	0,35186987	4,06525564	0
10051	H200016244	CACNB3	5,57138557	1,99407067	0,35791288	3,90361082	0
10373	H200017003	C7orf28B	5,56522955	2,41798976	0,43448159	5,68269818	0
5684	H200007729	GRIN2A	5,56325487	2,50400341	0,45009684	6,09613361	0
10619	H200017579	TMEM68	5,55746102	1,72169587	0,30979900	3,24945958	0
8061	H200011946	MSH4	5,55718993	2,49002590	0,44807284	6,01657294	0
9743	H200015439	PURB	5,55018566	3,20184352	0,57688944	13,06909540	0
7242	H200010537	PITX2	5,53922432	1,73622540	0,31344197	3,16781552	0
8885	H200013768	ST18	5,53829617	2,69649253	0,48688124	7,67819344	0
6899	H200009942	CBLB	5,53082903	2,82605180	0,51096351	6,97687341	0
3928	H200005201	NKX3-1	5,52056953	2,19925318	0,39837433	4,30541382	0
9924	H200015785	CRNN	5,51987805	2,77031518	0,50187978	7,24492918	0
1084	H200001287	TRAK1	5,51279788	2,22667557	0,40391025	4,68765996	0
4476	H200006035	TYRP1	5,51091886	2,32721143	0,42229100	5,76851547	0
11247	H200019348	IFT81	5,50770078	2,15668868	0,39157695	4,57260001	0

3487	H200004565	ZNF85	5,50760761	2,65550007	0,48215128	6,00462370	0
6986	H200010131	EDEM3	5,49612008	3,18279736	0,57909895	8,76586034	0
8900	H200013787	PRIC285	5,49109758	2,49975800	0,45523831	5,24774662	0
5788	H200007881	HS2ST1	5,48809393	2,34070581	0,42650615	4,81925571	0
7541	H200011073	ATP8B1	5,48358265	1,95734872	0,35694706	3,82694645	0
3773	H200004980	TBX19	5,48154064	2,69281005	0,49125059	6,74148324	0
10635	H200017608	FABP2	5,48113529	1,51129629	0,27572687	2,87284054	0
1387	H200001686	TMEM47	5,47993784	2,04783975	0,37369762	4,01593830	0
7021	H200010190	FAM38A	5,47740917	2,81178093	0,51334141	7,04843517	0
6711	H200009520	ERCC5	5,47287196	2,29556441	0,41944420	4,59179761	0
6831	H200009777	FLJ34870	5,46918857	2,79777480	0,51155208	7,15760226	0
9017	H200013965	ELF1	5,46698226	1,64954907	0,30172936	3,06933895	0
8239	H200012244	NUFIP1	5,46514960	2,42136221	0,44305507	5,11542485	0
7823	H200011558	ZNF398	5,46210841	2,26559606	0,41478416	5,27740133	0
9233	H200014318	CAMSAP1	5,45567014	1,46018312	0,26764505	2,81461243	0
8150	H200012108	PBX4	5,45502461	1,95613799	0,35859380	3,52739520	0
913	H200001073	FBXL5	5,45228018	2,17730783	0,39933895	4,64638433	0
8906	H200013797	LARP2	5,45166856	2,45459519	0,45024659	5,46111797	0
10949	H200018578	EEF1G	5,44400940	1,86920396	0,34335061	3,43172735	0
2408	H200003053	C9orf5	5,44248366	2,72875534	0,50138053	5,70953883	0
5445	H200007252	ZNF461	5,44183969	3,22747922	0,59308605	12,38235755	0
782	H200000920	IQSEC1	5,44090340	2,34663596	0,43129528	5,24370960	0
6806	H200009689	PNMA3	5,43756619	3,09908717	0,56994013	10,20325583	0
11532	H200020124	RBPSUH	5,43453656	2,99628901	0,55134214	8,00216060	0
8650	H200013232	PAX8	5,43249854	2,58892678	0,47656281	6,60611949	0
8692	H200013338	PPP1R3F	5,43002110	2,74207106	0,50498350	6,82249984	0
818	H200000962	LDB2	5,42551668	2,91269488	0,53685115	8,36512955	0
3447	H200004506	GUF1	5,42118287	1,64089128	0,30268141	3,06252314	0
10453	H200017258	C2orf37	5,41980716	2,50916377	0,46296182	6,08209760	0
10298	H200016771	EN1	5,41711284	2,73887426	0,50559668	7,10655119	0
10327	H200016900	TAS2R9	5,41544016	2,68799391	0,49635742	6,50628494	0
9609	H200015140	KIAA1446	5,41411205	2,51878557	0,46522598	6,08103071	0
6591	H200009295	CEP152	5,41116273	3,15861197	0,58372149	10,18363821	0
11867	H200021274	OR5P2	5,41076356	2,18758249	0,40430199	5,05521081	0
6499	H200009034	GRSF1	5,41050901	2,07832933	0,38412825	4,21337752	0
10263	H200016680	UGT2B7	5,40544483	2,28165036	0,42210224	4,65831341	0
8559	H200013005	LOC115110	5,40388137	1,74285172	0,32251850	3,48641214	0
8291	H200012380	METTL4	5,40144778	1,31828327	0,24406110	2,49159938	0
404	H200000466	HLA-DOB	5,40062262	3,04899065	0,56456280	11,51546816	0
11014	H200018860	PRDM15	5,39967534	2,40273653	0,44497796	5,22520491	0
5417	H200007199	SMPX	5,39892366	3,05826124	0,56645758	9,32000479	0
11086	H200019020	ZNF710	5,39875002	2,43924563	0,45181674	5,60742360	0
8141	H200012094	LOC144486	5,39500879	2,80828221	0,52053339	7,38724276	0
9953	H200015856	ARTS-1	5,39495171	3,09177938	0,57308750	7,67816566	0
9373	H200014642	DNA2L	5,39311239	2,63360937	0,48832829	5,72164967	0
8763	H200013502	WDR35	5,39133351	2,94701636	0,54662105	6,93505343	0
9561	H200015038	GAL3ST3	5,38653715	2,34521704	0,43538492	4,99526234	0
11282	H200019428	KAL1	5,38588315	3,17909057	0,59026356	6,95621481	0
9808	H200015569	CLEC4E	5,38496783	2,31903352	0,43064947	5,34623218	0
1133	H200001351	C2orf33	5,38055605	2,13978741	0,39768890	4,57951219	0
4037	H200005372	GSPT2	5,37750582	2,38529362	0,44356877	4,87298403	0
11545	H200020151	CNTROB	5,37644858	2,33260444	0,43385599	4,83269474	0
10790	H200018048	PHTF1	5,37325786	2,30318291	0,42863808	4,97938487	0
7134	H200010377	AKAP6	5,35880386	2,55685477	0,47713162	6,30918578	0
11329	H200019530	HMCN1	5,35821833	2,77356171	0,51762760	6,60262985	0
9648	H200015224	ZNF696	5,35603822	2,23328344	0,41696555	4,64357255	0
10304	H200016781	MARK3	5,35403215	2,74465486	0,51263324	6,70200360	0
1009	H200001196	VARSL	5,34787252	1,54144048	0,28823433	3,01777817	0
11856	H200021241	ATG10	5,34590069	3,06957043	0,57419144	9,65023606	0
10643	H200017625	C10orf88	5,34564594	1,25012915	0,23385933	2,40828279	0
9598	H200015125	POU4F1	5,34507347	2,70906248	0,50683354	6,74519313	0
4994	H200006670	ZNF592	5,34396383	2,18067250	0,40806274	4,48800069	0
5484	H200007318	HAP1	5,34317394	3,09834235	0,57986927	8,93282919	0
10357	H200016973	C1orf63	5,33417221	2,31617024	0,43421362	5,38373300	0
4499	H200006062	CRH	5,33306320	2,56113692	0,48023750	6,26527010	0
8126	H200012076	SLC2A13	5,32989599	2,60647062	0,48902842	6,46555965	0
8705	H200013364	TRIM27	5,32596625	1,33102438	0,24991228	2,57357824	0
3426	H200004481	CCDC76	5,32479666	2,43207643	0,45674541	4,99211848	0
5616	H200007606	CCDC54	5,32267865	3,29072877	0,61824675	9,31875615	0
10289	H200016747	FLJ16542	5,32116566	1,64639205	0,30940440	3,10105504	0
2480	H200003149	KIAA1737	5,31972729	1,57033768	0,29519139	3,00945596	0
9566	H200015053	PRRG3	5,31951998	2,52702032	0,47504668	5,49021930	0
11774	H200021008	MGC72104	5,31751185	2,46023502	0,46266658	5,30219352	0
444	H200000515	TCN1	5,31668830	2,51758608	0,47352524	7,23637249	0
10886	H200018333	SLC41A2	5,31572210	2,32935017	0,43820014	5,55674387	0
5486	H200007322	CHST3	5,31442458	2,42639528	0,45656783	5,48474642	0
210	H200000249	MYH3	5,30995652	2,36193698	0,44481287	5,38119870	0
5256	H200006995	COL5A2	5,30863534	2,18973445	0,41248538	4,76161531	0
4494	H200006056	RMND5A	5,30625883	2,15456375	0,40604196	4,39925839	0
3944	H200005222	HEXIM2	5,30588737	2,21952390	0,41831342	4,60008808	0
8843	H200013697	ZMYM4	5,30317294	2,35232711	0,44356975	5,23517434	0
9082	H200014065	CYorf15B	5,29462247	1,44397533	0,27272489	2,69665391	0
7216	H200010499	CDSN	5,29443589	1,70311475	0,32168012	3,31268640	0
8768	H200013514	ZNF684	5,28867841	2,19346780	0,41474781	4,60090570	0
7056	H200010251	SPAG17	5,28709756	2,69918369	0,51052277	6,57219257	0
10791	H200018054	BTN2A3	5,28519129	2,22449074	0,42089125	4,89373686	0
3357	H200004383	CRYBB1	5,28023783	2,94292258	0,55734659	7,74300209	0
10981	H200018721	KRTAP4-10	5,27968582	4,17740733	0,79122271	34,00214336	0
7526	H200011047	GRIA3	5,27318443	1,79948607	0,34125225	3,68130898	0
9310	H200014479	GOPC	5,26793593	2,37163291	0,45020155	5,48502307	0
4948	H200006617	CCNG2	5,26674325	1,81216989	0,34407789	3,29736762	0

7780	H200011496	C1orf25	5,26403201	3,44382309	0,65421773	14,91554388	0
7338	H200010691	TCEAL1	5,26034917	2,63620958	0,50114726	6,82104149	0
2794	H200003581	MYCN	5,25455021	1,94375697	0,36991881	3,76969695	0
8721	H200013396	PRKD3	5,25409612	1,69229734	0,32209105	3,19815702	0
10872	H200018268	ZFAND6	5,25033746	2,35663093	0,44885323	5,21423691	0
7262	H200010569	NF1	5,24913333	2,32595211	0,44311165	4,54491888	0
7907	H200011678	IL17B	5,24823602	2,57179529	0,49003042	5,73092657	0
4187	H200005611	ITSN1	5,24714183	2,56966723	0,48972704	6,05875569	0
4230	H200005674	C1orf27	5,24712042	1,43856135	0,27416206	2,69374630	0
2181	H200002744	SLC36A1	5,24543421	1,53847941	0,29329877	2,90043768	0
6911	H200009965	LMBRD1	5,24450743	2,65827867	0,50686908	6,57620038	0
9902	H200015728	KCNH4	5,24385011	2,44096475	0,46549095	5,63893164	0
8036	H200011902	LRRC31	5,24262356	2,34315578	0,44694336	5,40765572	0
4121	H200005516	MTMR3	5,23947566	1,92950670	0,36826332	3,62685575	0
9208	H200014285	TMEM69	5,23631845	2,16027328	0,41255575	4,42583413	0
2721	H200003487	PGAP1	5,23599842	2,25688593	0,43103258	4,88186801	0
7240	H200010535	HMG2L1	5,23587625	2,44211599	0,46641973	4,94996373	0
4180	H200005599	TAOK2	5,23563681	2,45353788	0,46862263	5,27411390	0
7862	H200011610	SORBS1	5,23530727	2,39918095	0,45826937	5,22277875	0
11262	H200019386	AVIL	5,23436862	2,26984980	0,43364347	4,89142939	0
9737	H200015425	CEACAM21	5,23385453	1,83953498	0,35146850	3,90766093	0
11874	H200021293	TST	5,23379389	1,63757493	0,31288487	2,92805482	0
9715	H200015393	XPR1	5,23363681	1,97661820	0,37767584	4,13095277	0
7584	H200011154	KBTBD3	5,23233610	2,49208641	0,47628561	5,93436122	0
2967	H200003822	PPP2R3A	5,23031596	2,47525594	0,47325170	5,57576133	0
10794	H200018091	CLTC	5,22679954	1,72225528	0,32950475	3,31455109	0
8075	H200011973	REXO1	5,22265375	2,46684158	0,47233489	5,28225138	0
10163	H200016497	PACS1	5,21890619	2,15691911	0,41328950	4,39931547	0
10136	H200016438	KIAA1797	5,21781834	2,66512581	0,51077397	6,30422344	0
8676	H200013297	FAM55C	5,21711713	2,31522499	0,44377478	5,07066945	0
7174	H200010438	SPTLC1	5,21671959	1,58290701	0,30342957	2,99543820	0
3843	H200005082	SIX1	5,21555134	2,62540896	0,50338091	6,31103867	0
11717	H200020773	CNTNAP5	5,21451883	2,34488208	0,44968331	5,38469247	0
7833	H200011570	TMEM126A	5,21435048	1,67614664	0,32144879	3,10145937	0
9555	H200015017	SSH2	5,21034796	2,79998060	0,53738841	7,22090175	0
9859	H200015648	C6orf194	5,20720692	2,62003730	0,50315598	6,50535103	0
677	H200000796	FUSIP1	5,20295970	1,90717864	0,36655649	3,74205421	0
8262	H200012290	HNF4G	5,20144775	2,37043899	0,45572677	5,49521110	0
6956	H200010056	GPR161	5,20129297	2,02892400	0,39008070	4,28102420	0
7434	H200010906	ADPRHL1	5,19826811	2,06836954	0,39789589	4,32250206	0
8056	H200011937	GRK4	5,19246537	3,31782115	0,63896837	10,38224543	0
1971	H200002467	ANKRD13A	5,19037978	1,23996794	0,23889734	2,29363483	0
10370	H200016992	PCYT1A	5,18996777	2,14058275	0,41244625	4,08561095	0
10031	H200016210	ZNF445	5,18814937	2,37111024	0,45702428	5,15784319	0
1008	H200001193	Gcom1	5,18596031	2,11660333	0,40814106	4,18000365	0
1135	H200001354	FBXO25	5,18261396	2,29777702	0,44336257	5,25279609	0
1005	H200001187	C1orf58	5,18211597	1,90491590	0,36759422	4,20323893	0
3199	H200004126	SNX1	5,18098105	2,33817086	0,45129886	5,06785651	0
8306	H200012423	ENTPD2	5,17843004	2,43136793	0,46951835	5,16997869	0
9	H200000008	MS4A2	5,17777860	2,60046217	0,50223510	7,59521974	0
9576	H200015081	NUDT12	5,17738754	2,36956088	0,45767501	5,23234891	0
6651	H200009410	ZNF300	5,17519203	1,70422524	0,32930667	3,22878733	0
7693	H200011353	SGOL1	5,17498422	3,00119804	0,57994342	7,40488310	0
4908	H200006576	RNF144	5,17287551	1,84719759	0,35709299	3,67452508	0
6980	H200010115	NSUN3	5,16696804	2,42393977	0,46912227	5,62307779	0
2833	H200003639	DPAGT1	5,16535265	2,51081677	0,48608816	5,31700607	0
5275	H200007016	ANKIB1	5,16437273	2,00693426	0,38861143	4,10138923	0
7980	H200011780	SLC30A3	5,16144654	2,55992126	0,49596973	5,52423124	0
7439	H200010927	LMAN1L	5,15387125	3,26011781	0,63255709	15,90486668	0
7445	H200010943	DDX3Y	5,15059916	1,93790506	0,37624847	3,63021045	0
3668	H200004827	LOC145783	5,14720838	2,87939012	0,55940811	6,88120588	0
5610	H200007596	ZNF446	5,14550048	2,17610836	0,42291481	4,50028103	0
8414	H200012673	RC3H1	5,14416868	2,43147701	0,47266666	5,67927103	0
5584	H200007549	LIM2	5,14416746	2,28384492	0,44396784	4,37133505	0
10975	H200018683	KIAA0232	5,13862054	2,17707721	0,42366958	4,55377596	0
6723	H200009543	VPS13C	5,13776561	2,30740574	0,44910685	4,78181499	0
10993	H200018797	ARSD	5,13192815	2,18176689	0,42513590	4,68273587	0
4156	H200005561	GAS1	5,13005082	3,21230202	0,62617353	10,79497691	0
3894	H200005154	RABGAP1	5,12942202	1,22779727	0,23936367	2,30892238	0
6042	H200008223	TMEM41B	5,12880477	1,64682672	0,32109366	3,09833743	0
7934	H200011714	SOCS2	5,12688301	2,58512458	0,50422929	5,51259429	0
11844	H200021203	ANKFY1	5,12527754	1,65886726	0,32366389	3,17096772	0
4979	H200006655	KIAA0232	5,12066793	1,94547454	0,37992593	3,72164065	0
9204	H200014279	SNAP23	5,11788953	1,78897555	0,34955337	3,48359940	0
8765	H200013508	HLA-A	5,11761971	2,74001133	0,53540737	6,24337360	0
11334	H200019564	KRTAP4-2	5,11661283	2,40380116	0,46980322	5,65889020	0
6133	H200008372	COL10A1	5,11646913	2,75214256	0,53789879	7,02909279	0
10780	H200018014	NEU4	5,11596946	1,71020670	0,33428790	3,42016578	0
10515	H200017394	PCDHB1	5,11549709	1,50923621	0,29503217	2,86849445	0
11762	H200020977	VEGFA	5,11113237	1,21544482	0,23780343	2,29039346	0
1137	H200001357	INSIG2	5,10874864	2,27276817	0,44487767	5,31042318	0
7551	H200011097	ZNF354A	5,10595180	2,34714991	0,45968901	5,30941058	0
10859	H200018230	SLC28A3	5,10506931	2,30537637	0,45158571	5,06195204	0
8355	H200012538	NRG3	5,10355227	1,65855369	0,32498025	3,12047596	0
8327	H200012478	C20orf141	5,10333903	2,89433849	0,56714604	10,64734186	0
7753	H200011458	KIAA1799	5,10122481	1,82626093	0,35800440	3,35517402	0
9287	H200014427	LONRF1	5,09963548	2,21327769	0,43400704	4,75782271	0
7215	H200010498	TM9SF1	5,09956344	2,49062443	0,48839954	4,79632114	0
949	H200001117	SACM1L	5,09834628	1,87380513	0,36753195	3,45172147	0
6611	H200009343	IBRDC1	5,09709058	2,88355767	0,56572620	7,96886424	0
9858	H200015647	IGF1R	5,09378932	1,32995539	0,26109352	2,50394485	0

11265	H200019392	FLJ25371	5,09332886	1,38977213	0,27286126	2,57941118	0
8086	H200011989	KNS2	5,09209762	2,48703381	0,48841047	5,79734243	0
4097	H200005475	PIGK	5,09051933	2,52345957	0,49571751	5,24927878	0
5134	H200006845	PF4	5,08918415	2,78272259	0,54679149	6,47486817	0
10637	H200017612	DOCK5	5,08909547	1,88491462	0,37038303	3,83372011	0
7288	H200010605	USP49	5,08465241	1,87963144	0,36966764	3,27591144	0
7382	H200010772	GPR160	5,08362267	2,84903148	0,56043331	5,78182206	0
4013	H200005337	ROCK2	5,07986687	1,27705557	0,25139548	2,44837499	0
3261	H200004233	MCTP2	5,07914734	2,34764256	0,46221194	5,30370164	0
11703	H200020712	ZNF85	5,07625109	2,70660419	0,53318958	7,81137140	0
3852	H200005097	NKX2-5	5,07601057	1,30165170	0,25643203	2,41591378	0
8792	H200013576	SLC36A1	5,07092058	2,29498894	0,45257836	5,18505076	0
11559	H200020222	MAG	5,06585183	2,80049238	0,55281766	7,35369557	0
10754	H200017949	FNBP1	5,06572640	1,35831483	0,26813821	2,62281277	0
11778	H200021022	ATP8B1	5,06100371	2,32007953	0,45842281	5,35030247	0
11592	H200020321	ABCC5	5,06059366	2,29734033	0,45396657	5,28873177	0
5276	H200007017	C7orf36	5,06010078	1,74597570	0,34504761	3,14591712	0
11726	H200020820	OR7E5P	5,05877512	2,22426398	0,43968430	4,87255350	0
8130	H200012082	SPG20	5,05775739	1,30812361	0,25863708	2,46198600	0
11306	H200019485	TM2D1	5,05347414	1,96635830	0,38911019	3,86769550	0
3817	H200005045	INPP5F	5,05293431	1,54795316	0,30634738	2,95035404	0
9136	H200014156	CLEC7A	5,05008317	2,27321395	0,45013396	4,56526241	0
8394	H200012623	HOXB5	5,04987506	1,49667008	0,29637765	2,94863507	0
10192	H200016554	COMMD2	5,04858879	1,68614285	0,33398300	3,10704133	0
5283	H200007025	F8A1	5,04689723	1,71174519	0,33916783	3,28179119	0
9415	H200014725	PLAGL1	5,04673903	2,50013071	0,49539528	5,72262004	0
8135	H200012087	NR1I2	5,04619724	2,52616345	0,50060735	6,15761557	0
3282	H200004263	LOC152485	5,04618732	2,08542160	0,41326678	4,03222546	0
5259	H200006999	PIK3R4	5,04568779	1,31751598	0,26111722	2,57968942	0
4001	H200005317	TMEM112	5,04433688	2,58090386	0,51164383	5,78371217	0
3623	H200004765	BDKRB2	5,04419281	2,43894792	0,48351600	5,47878704	0
3614	H200004750	LDOC1	5,04381524	1,69806644	0,33666309	3,32675390	0
11315	H200019503	CKM	5,04324404	2,07186684	0,41082026	4,60280909	0
11576	H200020265	KIAA1914	5,04099726	1,58245135	0,31391633	2,98052716	0
2265	H200002858	CASP8	5,03789983	2,10939514	0,41870526	4,80520761	0
7805	H200011533	KLF2	5,03357083	2,16050900	0,42921995	4,47522683	0
10220	H200016598	MAPK8	5,02765288	1,15475377	0,22968049	2,21939690	0
4902	H200006569	ITPKB	5,02406465	2,24587339	0,44702319	4,58415776	0
5774	H200007863	COL4A5	5,02382545	2,67867109	0,53319350	7,52962246	0
8714	H200013382	GPA33	5,02377843	2,18763307	0,43545572	4,76348726	0
10725	H200017887	CD4	5,02260812	2,58955985	0,51558071	6,79913685	0
7135	H200010378	IL2	5,02226662	2,46620480	0,49105414	6,18150610	0
7642	H200011253	CMTM7	5,02182855	2,51697344	0,50120657	5,60815425	0
9641	H200015208	KLK4	5,02137974	2,67136611	0,53199842	6,53357372	0
5762	H200007845	GOLGA8G	5,01968802	3,40414847	0,67815937	13,92605191	0
8787	H200013565	MAP7	5,01965963	1,59703956	0,31815694	3,03846897	0
441	H200000512	GSTM3	5,01919038	2,22221771	0,44274426	4,74322474	0
5455	H200007268	DPF3	5,01336759	2,41327970	0,48136899	5,45331514	0
11274	H200019409	TLN2	5,01172190	2,29466277	0,45785916	5,27327970	0
1175	H200001407	GPS2	5,01141638	1,54297926	0,30789285	3,10042371	0
6237	H200008508	ELK1	5,00735575	1,49366204	0,29829357	2,71838761	0
9542	H200014983	PRKD3	5,00584592	2,15234843	0,42996698	4,54771224	0
11032	H200018899	PLDN	5,00566055	2,45518695	0,49048211	5,31613328	0
10775	H200018002	KCNK13	5,00197214	2,07192075	0,41422077	4,74983160	0
8149	H200012107	HLA-E	5,00183764	2,36090421	0,47200737	5,19387921	0
619	H200000727	EGR4	5,00035488	2,39133592	0,47823324	5,50941917	0
4373	H200005914	KLRC1	4,99563560	2,22866480	0,44612237	4,68131090	0
9006	H200013949	EDG1	4,99349456	3,12264660	0,62534295	13,92977932	0
3857	H200005106	PA2G4	4,99294257	2,24123031	0,44887965	4,91909234	0
11040	H200018914	LOC283970	4,99211730	2,55120155	0,51104600	6,03751501	0
10326	H200016899	TAS2R8	4,99043433	2,42720707	0,48637191	5,46147870	0
11295	H200019453	IGFBP7	4,99029364	3,18479131	0,63819718	19,93481130	0
415	H200000478	PGC	4,98888733	2,28233352	0,45748348	4,93855148	0
727	H200000850	FKBP10	4,98673577	1,43146764	0,28705504	2,69947290	0
8118	H200012067	ABCD2	4,98648144	2,98263373	0,59814396	11,15383966	0
9869	H200015662	TIA1	4,98323256	1,52068502	0,30516036	2,88837301	0
8667	H200013277	USP49	4,98166867	2,42934679	0,48765724	5,37151520	0
8336	H200012498	ACTR8	4,98104106	2,00035968	0,40159470	4,38034491	0
10763	H200017973	LONP2	4,97785583	2,18495654	0,43893528	4,39493977	0
3666	H200004825	DGKH	4,97273649	1,90230611	0,38254714	3,71227963	0
2771	H200003551	MBD2	4,97243467	2,38469002	0,47958197	5,15149880	0
10715	H200017858	THSD4	4,97090002	2,09997232	0,42245314	4,39460291	0
432	H200000498	CRYAB	4,96835377	2,12502019	0,42771113	4,38248903	0
6114	H200008343	SLC32A1	4,96747043	1,93833616	0,39020588	3,57338000	0
11736	H200020861	MYL3	4,95970237	2,25917182	0,45550552	4,81220804	0
5390	H200007157	SUV39H2	4,95822449	2,49782194	0,50377347	6,48031905	0
11198	H200019207	GPR174	4,95356427	2,26686858	0,45762373	5,06575903	0
10295	H200016765	WDR51B	4,95096568	2,25636930	0,45574327	5,06021401	0
482	H200000563	CSF3	4,94793955	1,43094045	0,28919926	2,62857469	0
10292	H200016753	POLI	4,94756060	2,21794015	0,44828964	5,18407908	0
5580	H200007539	STARD4	4,94668826	1,78483801	0,36081473	3,45667667	0
1103	H200001312	ACBD3	4,94646891	1,51699496	0,30668240	2,86929779	0
9668	H200015277	C3orf20	4,94561949	2,25384919	0,45572636	4,90211632	0
8266	H200012313	SPAM1	4,94411951	2,20480099	0,44594411	4,92181131	0
7227	H200010521	RAPGEF4	4,94083198	2,34510227	0,47463712	5,11955497	0
7045	H200010233	NKD1	4,93791291	2,37495320	0,48096296	5,42073028	0
8881	H200013764	CILP	4,93756596	2,42893881	0,49193040	5,46142765	0
9545	H200014992	LOC149692	4,93705638	2,46251998	0,49878304	5,71759563	0
9234	H200014319	SSTR2	4,93624994	2,48538685	0,50349696	6,97835346	0
311	H200000363	PSMB2	4,93620148	1,54593314	0,31318275	2,85075369	0
2424	H200003076	PBX1	4,92655032	2,69539901	0,54711691	5,29502467	0

7638	H200011248	SLURP1	4,92404059	2,86186069	0,58120169	6,85879108	0
8547	H200012986	REV1L	4,92262028	2,24499798	0,45605752	4,92702337	0
10881	H200018320	SH2D3C	4,92243032	1,60274073	0,32559948	3,00106436	0
11199	H200019208	MBNL2	4,91739702	2,16149613	0,43956104	4,41479915	0
2550	H200003240	CA1	4,91345412	2,33503333	0,47523255	5,17369650	0
4000	H200005316	PGLYRP4	4,90905456	2,63858265	0,53749304	6,06532025	0
7189	H200010462	RABIF	4,90623203	2,32041567	0,47295270	5,00007757	0
3496	H200004577	TEAD1	4,90469288	1,12969285	0,23032897	2,20158253	0
6916	H200009979	NFKBIZ	4,90344972	2,11194011	0,43070496	4,49035451	0
11507	H200020061	TP53	4,90135838	2,06590187	0,42149578	4,18703860	0
11174	H200019156	EGR1	4,89931254	2,14743676	0,43831389	4,27111794	0
6174	H200008428	LPP	4,89799859	1,88422422	0,38469268	3,57440535	0
10316	H200016853	EPHB1	4,89547967	2,31840190	0,47358013	5,50917535	0
4025	H200005357	SLC6A16	4,89481474	2,54029954	0,51897767	5,87475153	0
10196	H200016560	C14orf104	4,89358833	2,00788052	0,41030842	3,94414761	0
2280	H200002880	06.mar	4,89093496	1,82923815	0,37400582	3,61581031	0
1068	H200001267	DKFZP564O0	4,88300901	2,63543518	0,53971540	7,81095660	0
9120	H200014122	RNF103	4,88174639	1,20199628	0,24622260	2,31229877	0
6828	H200009764	MAX	4,87987207	2,58004735	0,52871209	6,55283658	0
11396	H200019714	CPM	4,87876940	2,14328841	0,43930923	4,55542393	0
1187	H200001421	BTBD6	4,87589393	1,66396182	0,34126292	3,29180620	0
8382	H200012588	TMOD2	4,87451633	1,89952558	0,38968494	3,99195743	0
2330	H200002945	RBMS2	4,87052230	2,19046515	0,44973927	4,35600562	0
8946	H200013862	SH3KBP1	4,86494528	1,06190058	0,21827596	2,10199141	0
3281	H200004262	FYB	4,86356510	1,34149442	0,27582532	2,49983127	0
11070	H200018991	HSPA14	4,85649806	3,16947037	0,65262466	6,29683079	0
2889	H200003717	DISC1	4,85642519	2,36461636	0,48690472	5,02397930	0
9978	H200015949	OR12D3	4,85480388	1,98009448	0,40786292	4,13776288	0
9758	H200015475	ISLR2	4,85435055	2,36001656	0,48616525	5,13272357	0
7601	H200011184	NTN4	4,85363370	1,84191580	0,37949213	3,84705731	0
8959	H200013882	PAK3	4,85152574	2,48834256	0,51289897	5,40052145	0
6829	H200009772	SLC24A3	4,85053019	2,41239579	0,49734683	5,15947931	0
11240	H200019337	S100PBP	4,84949438	2,51581503	0,51877883	5,66617554	0
11371	H200019676	LRP11	4,84792306	1,75472443	0,36195385	3,56793294	0
8024	H200011865	CCR8	4,84774242	2,39594210	0,49423874	5,47091142	0
11142	H200019104	EIF2C2	4,84618598	1,49571769	0,30863811	2,91076621	0
9397	H200014685	SEZ6L	4,84591764	2,87952894	0,59421747	12,80958855	0
6930	H200010010	CCDC40	4,84145685	2,41353213	0,49851361	5,49902947	0
9625	H200015168	SOX7	4,83824144	1,91905734	0,39664357	3,89684485	0
7652	H200011277	CXCL11	4,83588340	2,62425874	0,54266378	6,03254993	0
3555	H200004664	C12orf53	4,83510863	2,02975433	0,41979498	4,32963311	0
8334	H200012494	SCN8A	4,83260450	2,01323621	0,41659445	3,85761094	0
8109	H200012044	PADI4	4,83071382	2,34203571	0,48482187	6,00505304	0
8782	H200013558	ZFP64	4,83055559	2,01331807	0,41678810	4,12355611	0
9496	H200014876	CARD6	4,83027870	1,87169219	0,38749155	3,80735928	0
8652	H200013236	PIGA	4,82678242	2,33150789	0,48303563	5,21374302	0
2125	H200002672	CCDC14	4,82478614	1,31030189	0,27157720	2,41589393	0
6448	H200008881	C20orf177	4,82458960	1,41214109	0,29269662	2,71031643	0
4091	H200005468	KCTD5	4,82441241	1,57887807	0,32726847	2,95381679	0
2404	H200003049	NELL1	4,82356777	1,94155862	0,40251505	4,23661356	0
10777	H200018006	PBOV1	4,82312756	2,15199553	0,44618259	4,54074087	0
9995	H200016077	GRM6	4,82091204	1,64838163	0,34192319	3,27201147	0
4581	H200006183	APOD	4,82019194	2,01998370	0,41906707	4,93600641	0
11447	H200019897	UGT3A1	4,81918420	2,25605375	0,46814018	4,76167054	0
9396	H200014684	LUZP2	4,81525435	2,29874681	0,47738845	4,91206349	0
2929	H200003771	CYB5D1	4,81371381	2,62858279	0,54606129	5,07686100	0
8965	H200013890	C6orf12	4,81303617	2,21687623	0,46059829	4,77882212	0
7641	H200011252	ARL6IP4	4,80975599	2,34587395	0,48773242	4,86912699	0
9650	H200015231	ADAM12	4,80831071	3,00886025	0,62576244	16,01704790	0
9485	H200014846	LRRC8B	4,80760620	2,88413658	0,59991115	11,23226680	0
3656	H200004812	MANEA	4,80702308	1,56484022	0,32553208	2,97367810	0
8905	H200013794	CEP57	4,80564660	1,78932101	0,37233720	3,29243106	0
9687	H200015333	RDH11	4,80393102	2,24901481	0,46816135	4,98403247	0
5140	H200006852	CD160	4,79625207	2,35365487	0,49072793	5,03639676	0
10755	H200017951	STK11	4,79599617	2,13249175	0,44464000	3,74506667	0
4726	H200006356	DNASE1L1	4,79509561	1,45676118	0,30380232	2,58614045	0
10275	H200016708	C9orf39	4,79437718	1,63304247	0,34061619	3,25891341	0
8390	H200012608	CLEC5A	4,78657405	2,03015668	0,42413565	4,42138605	0
5869	H200008002	CHPT1	4,78613756	1,96005278	0,40952705	3,86092819	0
10237	H200016620	MRPS6	4,78583747	2,33121132	0,48710625	4,91365636	0
10972	H200018673	PDLIM5	4,78512680	2,23137577	0,46631487	4,90084582	0
487	H200000570	CXCL10	4,78389680	2,17671389	0,45500854	4,90593253	0
5971	H200008122	B3GNT2	4,78376441	1,24206412	0,25964157	2,36820317	0
6729	H200009557	CUGBP1	4,78327917	2,30752506	0,48241488	5,28948088	0
1779	H200002201	ACTR3B	4,78303703	1,45803431	0,30483442	2,70001612	0
10444	H200017244	PYCR2	4,77707408	2,17053484	0,45436491	4,22992410	0
8952	H200013872	PER2	4,77694527	2,01912742	0,42268171	4,33043727	0
9008	H200013952	TRAK2	4,77282507	1,85830637	0,38935145	3,86837822	0
5983	H200008137	DPYSL2	4,77135444	1,15150399	0,24133692	2,21245923	0
4055	H200005403	HTRA3	4,77119693	2,32912423	0,48816351	5,14251525	0
5211	H200006939	CXCL16	4,77045976	2,01082642	0,42151627	4,15661657	0
3099	H200003999	RPS6KC1	4,76890289	1,56557387	0,32828806	2,95097061	0
3361	H200004390	DMC1	4,76808704	2,46864456	0,51774318	5,62138198	0
11848	H200021211	ARID2	4,76153801	2,19186567	0,46032725	4,99102624	0
9798	H200015557	SMARCC2	4,76059181	1,64257874	0,34503667	3,12012017	0
6921	H200009988	NFIA	4,76024636	2,15334455	0,45235990	4,74710419	0
3342	H200004352	HBB	4,75982348	1,61026111	0,33830269	3,13110498	0
11722	H200020805	CES7	4,75144694	2,02338181	0,42584540	4,15847125	0
9482	H200014843	CCBE1	4,75070388	2,21272784	0,46576842	4,89696929	0
110	H200000128	RAB5C	4,74958965	1,46231831	0,30788309	2,80110179	0
5954	H200008100	ZNF644	4,74736453	2,27748339	0,47973636	4,61131096	0

2740	H200003511	RAB27B	4,74720529	2,19557911	0,46249930	4,82105628	0
1405	H200001707	CCDC49	4,74709483	1,60152457	0,33736941	2,82883327	0
3895	H200005155	TMEM142A	4,74685097	1,72112918	0,36258336	3,09142820	0
11546	H200020153	MKRN1	4,74605011	1,76446664	0,37177581	3,48006291	0
10826	H200018165	OR51E2	4,74552717	2,07373332	0,43698692	4,34791459	0
10604	H200017557	CHML	4,74313939	1,80371102	0,38027789	3,70069097	0
6315	H200008631	KPNA5	4,74213997	1,61998785	0,34161536	3,24704618	0
2526	H200003209	XYLT1	4,73991695	2,51498210	0,53059624	6,64590234	0
8644	H200013218	ALOX12B	4,73188562	2,08029354	0,43963310	4,43808550	0
6480	H200008964	C16orf52	4,73135810	2,20754795	0,46657807	4,54367533	0
10967	H200018630	NLN	4,72899822	1,95716404	0,41386441	3,90919420	0
496	H200000581	RPN1	4,72671451	1,34926126	0,28545436	2,41127258	0
11712	H200020761	C14orf81	4,72629693	2,06857117	0,43767271	4,60684659	0
3037	H200003917	GOLPH3L	4,72420595	1,33792028	0,28320532	2,59610618	0
9928	H200015792	SLC17A6	4,71893355	2,11631324	0,44847278	4,46799485	0
10557	H200017489	ZNF223	4,71826789	1,48335967	0,31438649	2,86172061	0
8808	H200013621	OPA1	4,71641395	1,15545091	0,24498505	2,22143330	0
956	H200001126	GNE	4,71526871	1,50339414	0,31883531	3,04708555	0
6665	H200009432	NUBPL	4,71423309	2,05078419	0,43501968	4,89318467	0
1139	H200001359	C2orf30	4,70990122	1,67285306	0,35517795	3,00806110	0
11584	H200020289	SORCS3	4,70938781	2,47062213	0,52461641	5,83838183	0
10793	H200018087	CDH20	4,70652680	2,11018254	0,44835239	4,67483545	0
10662	H200017720	FLJ25006	4,70319434	2,83689949	0,60318568	10,17290603	0
10319	H200016882	HR	4,70234912	1,92697849	0,40979060	3,90276597	0
10438	H200017236	LIMK2	4,69465111	1,51617970	0,32295897	3,06409410	0
8025	H200011872	NRG2	4,69215420	2,11572221	0,45090637	4,45067230	0
7852	H200011595	PIGN	4,69158200	1,73214019	0,36920173	3,26829670	0
9829	H200015600	KIAA0143	4,69138920	2,36038271	0,50313087	5,15755037	0
8383	H200012589	SYN3	4,68586233	2,91238781	0,62152654	16,49109087	0
7047	H200010238	THBS1	4,68321052	1,93760407	0,41373414	3,82277005	0
1072	H200001271	FLJ32745	4,68212368	1,95128550	0,41675223	4,18029406	0
10458	H200017268	DOPEY1	4,67832471	2,22037792	0,47460962	4,60584039	0
10988	H200018743	TCAM1	4,67421701	2,11798241	0,45312026	4,41918936	0
8748	H200013461	C19orf6	4,67316674	2,16852422	0,46403742	3,88257879	0
11155	H200019121	RAB11FIP1	4,66952134	2,46745963	0,52841811	5,86005572	0
5685	H200007731	SNTG1	4,66915118	2,28326187	0,48901005	4,82863110	0
6137	H200008380	RAB20	4,66787467	2,51257813	0,53827026	4,85007738	0
4897	H200006564	FCGR2B	4,66677236	2,22778664	0,47737204	4,95981602	0
5227	H200006963	SLC35D1	4,66600235	2,10689201	0,45154114	4,20256687	0
3720	H200004908	C9orf52	4,66415382	2,30330693	0,49383168	5,04704745	0
10215	H200016592	FLJ14803	4,66304623	2,42378147	0,51978500	4,80673509	0
9969	H200015879	HIST1H3J	4,66229303	2,20517860	0,47298155	4,63159439	0
2855	H200003664	C20orf100	4,66159017	2,10980136	0,45259263	3,44605243	0
10118	H200016399	CCDC34	4,66089764	2,08114040	0,44651064	4,42138278	0
8580	H200013053	SLC4A7	4,65946067	1,75098792	0,37579197	3,21580929	0
6615	H200009353	TMEM134	4,65804001	1,19517227	0,25658265	2,19811573	0
11850	H200021215	BTBD8	4,65748418	2,49757979	0,53625084	5,64124351	0
3811	H200005033	MAFF	4,65740302	1,43568381	0,30825844	2,79246101	0
9490	H200014857	EPN3	4,65387097	2,09803453	0,45081493	4,36520729	0
2760	H200003539	C16orf30	4,65332378	2,20982230	0,47489115	4,72569786	0
6070	H200008279	ZBTB34	4,65257437	1,42416545	0,30610267	2,47824738	0
5927	H200008069	XPO7	4,65164474	1,25726966	0,27028497	2,39191456	0
7882	H200011646	KCTD12	4,65116547	2,33733883	0,50252756	5,92717421	0
5566	H200007509	P53AIP1	4,64904731	1,93641295	0,41651823	3,86467526	0
5619	H200007614	LOC153682	4,64711573	1,30323424	0,28043938	2,44526185	0
10324	H200016897	SSBP1	4,64422052	2,15074965	0,46310240	4,74660450	0
10947	H200018575	RAVER2	4,64243533	2,53815100	0,54672835	7,27007136	0
9246	H200014344	EBI3	4,64027064	1,52011560	0,32759201	2,69011903	0
3000	H200003867	C10orf46	4,63997073	1,58170648	0,34088717	2,84794531	0
10628	H200017598	MAS1L	4,63821561	1,88936986	0,40734843	4,12271970	0
10854	H200018219	HMGB1	4,63537362	2,44637460	0,52776212	6,42226867	0
3103	H200004003	CCNE2	4,63535436	2,24700467	0,48475359	3,95702626	0
7079	H200010290	MSL3L1	4,63508582	2,43807922	0,52600520	4,85468703	0
10768	H200017985	SERHL2	4,63430602	1,85024321	0,39924925	3,80304732	0
11851	H200021222	C15orf27	4,63222052	1,93687728	0,41813149	4,04231812	0
10007	H200016141	ABHD5	4,62930983	2,22105049	0,47978005	4,70652617	0
3098	H200003997	GFPT2	4,62823697	2,22387615	0,48050158	3,61757785	0
5881	H200008015	PTPRJ	4,62779456	1,99514053	0,43112124	4,10118408	0
3102	H200004002	TRIM5	4,62754067	1,88096595	0,40647205	3,51539383	0
6696	H200009492	C7orf28A	4,62638774	2,10133740	0,45420694	4,51079287	0
10198	H200016562	SLC22A8	4,62592460	2,12497771	0,45936281	5,20802424	0
6818	H200009733	ALMS1	4,62366585	2,38136024	0,51503727	5,19777611	0
2411	H200003059	TCF12	4,62267752	1,90007925	0,41103435	3,37146635	0
5242	H200006980	SELL	4,61909686	1,99913906	0,43279869	4,06937072	0
10891	H200018349	RAD50	4,61699537	2,21952714	0,48072977	4,95989142	0
9780	H200015523	HTR7P	4,61698449	1,38257332	0,29945375	2,63232836	0
10008	H200016142	LOC221272	4,61434681	1,96290505	0,42539175	4,31091094	0
7659	H200011290	CAP1	4,61328764	1,68547104	0,36535139	3,14775818	0
5458	H200007272	CHRNA10	4,60874874	2,13246165	0,46269861	4,57081440	0
7917	H200011694	EXOC6	4,60826575	1,96601737	0,42662847	4,13463093	0
5583	H200007548	RFX3	4,60595345	2,74366318	0,59567757	5,94483862	0
6135	H200008375	ANK3	4,60578350	2,90374260	0,63045573	12,86062598	0
5270	H200007010	COL15A1	4,60265569	2,12583191	0,46187072	4,48795966	0
8877	H200013757	BSND	4,60146483	1,52318309	0,33102135	2,68186496	0
8659	H200013257	PCDH21	4,59815735	1,33723720	0,29082023	2,52323383	0
10183	H200016534	NDE1	4,59671944	1,45676287	0,31691359	2,67639065	0
2520	H200003201	NPTN	4,59550189	1,84931698	0,40241894	3,78976569	0
1725	H200002110	ANKRD17	4,59352101	2,09203376	0,45543141	3,89056288	0
7621	H200011213	ALG10	4,59307986	2,36619201	0,51516457	5,43928874	0
6026	H200008195	DVL3	4,59300560	1,63847028	0,35673161	3,07692033	0
10054	H200016247	LRRC62	4,59205998	2,43863465	0,53105462	6,14496306	0

11845	H200021206	OPA1	4,59179634	2,21991110	0,48345156	4,73041275	0
10825	H200018162	IDS	4,58730613	1,09475020	0,23864773	2,10334701	0
3434	H200004491	OSGIN2	4,58595286	2,87611460	0,62715747	10,21584050	0
10971	H200018654	CXorf45	4,58462544	2,12333455	0,46314243	4,61693954	0
11338	H200019573	PAIP2	4,58336481	2,14613994	0,46824550	4,54931575	0
2763	H200003542	ELOVL1	4,58174584	1,41122199	0,30800966	2,77288395	0
9291	H200014437	VTI1A	4,57974509	2,14618345	0,46862509	4,44245191	0
1808	H200002235	RP11-301I17.	4,57530668	1,85649867	0,40576486	3,84711476	0
851	H200001003	IPO7	4,57430951	1,92528652	0,42089118	4,39470986	0
9525	H200014945	KCND2	4,57163098	2,08177626	0,45536839	4,53364940	0
899	H200001058	BAG5	4,57002662	1,91120733	0,41820486	3,94242534	0
9532	H200014964	PCDHB7	4,56851902	2,53658673	0,55523173	5,87840306	0
5303	H200007050	ARHGDIB	4,56704654	1,72793676	0,37834884	3,13859388	0
8858	H200013722	CYP21A2	4,56579750	2,14545127	0,46989628	4,53327014	0
8594	H200013094	ALDH16A1	4,56543662	2,50110172	0,54783407	4,91134636	0
5332	H200007085	TSPAN8	4,56283575	1,60427163	0,35159531	3,04249493	0
8603	H200013114	HP	4,55722986	1,18070439	0,25908379	2,16520158	0
9983	H200015992	OR1D2	4,55717291	2,31622550	0,50825930	5,25572149	0
2738	H200003508	C5orf21	4,55442912	1,03545752	0,22735177	2,04565310	0
7137	H200010381	INSR	4,55392813	2,02221210	0,44405885	4,32261948	0
10323	H200016895	SSBP1	4,55191827	1,31627792	0,28916994	2,50976035	0
3370	H200004399	RFK	4,54906223	1,83991685	0,40446069	3,77271364	0
9999	H200016095	OR3A1	4,54684990	2,08236872	0,45798053	4,51550254	0
2981	H200003840	MRPS10	4,54491612	1,88741435	0,41528035	3,78961190	0
3084	H200003980	C3orf52	4,54046029	2,02721557	0,44647799	4,12569508	0
7455	H200010954	CMTM5	4,53986442	2,03659809	0,44860328	4,15225283	0
3456	H200004523	GDF8	4,53768762	1,42386183	0,31378578	2,71904748	0
8552	H200012996	SLC4A8	4,53743022	2,09775709	0,46232272	4,55136821	0
5830	H200007947	UBE1DC1	4,53571384	1,68185855	0,37080350	3,15700821	0
6130	H200008369	TRIM14	4,53415019	1,73634176	0,38294756	3,89145878	0
10638	H200017614	NOC2L	4,53295883	1,66908008	0,36820985	3,39284986	0
2352	H200002974	NLGN4Y	4,52696876	1,52933165	0,33782686	2,88355365	0
5459	H200007277	ZDHHC15	4,52537351	2,07737694	0,45905094	5,05556188	0
10925	H200018511	CDC14B	4,52002402	2,27753178	0,50387603	5,40590960	0
7130	H200010372	EPHX1	4,51884046	1,58385459	0,35050022	3,00212352	0
752	H200000879	REG1B	4,51872481	1,87599682	0,41516067	3,82072678	0
5058	H200006750	AFAP	4,51630788	1,11990179	0,24796843	2,17786424	0
9674	H200015293	YES1	4,51301397	1,93262192	0,42823309	3,99929592	0
3457	H200004524	PPAP2A	4,50998458	1,58018468	0,35037474	2,90142671	0
10963	H200018606	KCNIP3	4,50845525	2,44759899	0,54289083	7,60689756	0
11242	H200019340	USP38	4,50401417	1,41034971	0,31313172	2,76551636	0
6763	H200009625	ADH6	4,49658132	2,04954730	0,45580123	4,17143260	0
9597	H200015123	PIK3R2	4,49606568	1,66799210	0,37098927	3,05344891	0
7143	H200010391	GCKR	4,49521397	1,81013403	0,40268028	3,63342116	0
8779	H200013554	PART1	4,49358504	1,92901761	0,42928254	4,04947004	0
11790	H200021052	HLA-DMB	4,49208926	1,98990780	0,44298047	4,18093577	0
5093	H200006797	CLCN7	4,49062476	1,41795264	0,31575843	2,72626658	0
9177	H200014237	UNC5B	4,48941006	2,03120982	0,45244471	3,93197632	0
3484	H200004562	LOC644538	4,48842521	2,17532689	0,48465259	4,74944775	0
5383	H200007148	CD34	4,48735654	2,25472057	0,50246076	5,14947859	0
7437	H200010915	LOC158301	4,48231652	2,05841250	0,45922962	4,25073134	0
836	H200000986	CPD	4,48172198	1,32513911	0,29567633	2,47177121	0
10745	H200017933	NEBL	4,47934403	2,98814551	0,66709444	24,86839132	0
8562	H200013015	MIER3	4,47558279	2,02404840	0,45224242	4,45182185	0
11286	H200019436	PDE4B	4,47518473	2,03736768	0,45525890	4,55857185	0
11048	H200018937	LOC92154	4,47478107	1,14419913	0,25569947	2,24459195	0
11795	H200021060	PABPC1	4,47440126	1,97917136	0,44233211	4,42498147	0
8712	H200013380	AOC2	4,47087771	2,68536901	0,60063575	9,90569402	0
9688	H200015338	RNF5	4,47068549	2,10689785	0,47126953	4,42713605	0
8956	H200013878	C18orf1	4,46138706	1,91395700	0,42900492	4,17183778	0
3425	H200004480	ATF6	4,46093241	1,94892509	0,43688738	3,90568901	0
430	H200000494	RLBP1	4,45367426	1,27489761	0,28625749	2,38291010	0
4663	H200006275	CAPN2	4,45347434	1,23018085	0,27622947	2,28802849	0
4628	H200006235	PSMF1	4,45289012	1,99104485	0,44713541	4,17764640	0
6642	H200009395	C17orf51	4,45220927	1,72029095	0,38639041	3,29278999	0
5027	H200006711	F2	4,45141665	1,69308973	0,38034852	3,12934005	0
5744	H200007820	CD99L2	4,45135773	1,80162902	0,40473697	3,56473344	0
6116	H200008348	SNAPC1	4,44945244	1,79289987	0,40294843	3,66499127	0
3733	H200004925	BMP2K	4,44935777	2,53045716	0,56872414	6,01310016	0
9779	H200015518	NKX2-8	4,44852375	2,47939061	0,55735133	8,38172860	0
3292	H200004279	ID4	4,44296512	2,04830927	0,46102304	4,17257391	0
8110	H200012045	CTAGE5	4,44040761	1,71132127	0,38539734	3,33586671	0
10138	H200016443	LOC391722	4,43720212	1,96783930	0,44348652	4,26492715	0
11801	H200021080	ZNF333	4,43526069	1,51963561	0,34262600	2,72305652	0
5287	H200007030	GRM7	4,43339906	2,11475844	0,47700611	4,58913215	0
9697	H200015352	C12orf59	4,43164184	1,94750999	0,43945564	4,14979858	0
7956	H200011748	PIAS2	4,42889234	1,12820805	0,25473820	2,16643093	0
7396	H200010814	C14orf148	4,42459962	2,02894577	0,45856031	4,29683970	0
7172	H200010434	FLJ36840	4,42327177	2,45466542	0,55494339	4,45282903	0
10703	H200017802	SEPT5.	4,42315670	1,24599558	0,28169827	2,24890637	0
10851	H200018215	MERTK	4,42084958	1,36081491	0,30781751	2,63097839	0
4796	H200006439	GGCX	4,42052739	1,89135227	0,42785670	3,75968594	0
5688	H200007734	CYP2C9	4,41583214	1,88150388	0,42608139	3,91412740	0
4275	H200005752	GNB3	4,41326085	1,54074814	0,34911785	2,90560900	0
10325	H200016898	TAS2R7	4,41218810	2,07930551	0,47126402	4,20113944	0
11105	H200019051	ETFDH	4,41011272	1,91996181	0,43535436	4,15313452	0
6833	H200009780	SEMA4A	4,40936326	2,28747009	0,51877560	4,91671941	0
6703	H200009504	MMP25	4,40911687	1,88950171	0,42854426	4,01859357	0
11749	H200020933	ANAPC5	4,40903800	1,62491029	0,36854078	2,96488713	0
10880	H200018317	C9orf106	4,40399866	1,88546542	0,42812579	3,97160204	0
10820	H200018157	PPP1R3C	4,40317296	1,89799424	0,43105148	4,12260383	0

11868	H200021275	OR5P3	4,40196276	1,86746741	0,42423517	3,92385884	0
608	H200000714	S100P	4,40183355	1,37822680	0,31310289	2,74357028	0
10675	H200017766	PCDHB4	4,39963039	1,87126713	0,42532371	3,92972333	0
9887	H200015691	SC4MOL	4,39936618	1,86023959	0,42284264	3,85063386	0
6187	H200008445	CYP4F12	4,39728519	1,69005391	0,38434030	3,13102676	0
312	H200000364	EGR2	4,39516667	1,88574204	0,42904904	4,01494517	0
10518	H200017404	IKZF2	4,39417788	1,86916067	0,42537210	3,84799412	0
11261	H200019385	ZNF493	4,39203097	2,01424033	0,45861251	4,22437900	0
6837	H200009799	MGC34824	4,38358257	1,81784039	0,41469286	3,68203792	0
8091	H200011999	OIP5	4,38348116	1,40116570	0,31964679	2,70608066	0
5143	H200006857	MGST2	4,38153220	1,68850523	0,38536867	3,75417846	0
9590	H200015113	IFNA2	4,38066886	2,57091873	0,58687813	9,47879223	0
3335	H200004344	CCNDBP1	4,37955977	1,58903476	0,36282979	2,90014303	0
11757	H200020957	WBSCR17	4,37903479	1,94173285	0,44341572	4,32994868	0
5777	H200007866	SOCS5	4,37630518	1,06691383	0,24379329	2,02939961	0
2848	H200003657	KIF21A	4,37596839	1,69686582	0,38776921	2,97498092	0
10104	H200016342	ZNF442	4,36355593	1,96988378	0,45144002	4,51437664	0
11839	H200021198	DONSON	4,36277959	1,60258656	0,36733154	3,20068019	0
6660	H200009426	MYLK	4,35822889	1,77790080	0,40794113	3,50916834	0
10978	H200018708	MCM3APAS	4,35578389	1,79742542	0,41265257	3,76810020	0
6360	H200008710	ZDHHC21	4,35541853	1,50194319	0,34484474	2,83602468	0
5373	H200007137	IGF1	4,35513167	1,85377962	0,42565409	3,76535241	0
6823	H200009749	SETBP1	4,34465659	1,29743715	0,29862824	2,41670028	0
7843	H200011584	ZNF22	4,34114679	1,94642227	0,44836592	4,51566200	0
10906	H200018426	ASTN2	4,34064336	1,95670562	0,45078700	4,17947263	0
9486	H200014847	UBE1	4,33994333	2,00717235	0,46248815	4,85382572	0
10620	H200017582	KIAA1456	4,33961530	3,11767089	0,71842103	16,56056017	0
9914	H200015767	MIA3	4,33841072	1,60313286	0,36952077	3,03341290	0
788	H200000926	GATAD2B	4,33777337	1,16617823	0,26884259	2,17090834	0
2546	H200003236	ACAD11	4,33659299	1,52902651	0,35258704	2,67734700	0
6838	H200009800	LOC197135	4,33623418	1,93488966	0,44621429	4,02856128	0
1822	H200002257	PRPSAP2	4,33327135	1,33196471	0,30738087	2,34375243	0
7572	H200011134	ARSF	4,33184037	2,04033139	0,47100798	4,07627187	0
7053	H200010248	TMEM28	4,33012914	1,73526632	0,40074239	3,48022650	0
10509	H200017353	PMS2L5	4,32803834	1,75905635	0,40643271	3,46039055	0
7540	H200011072	MLLT4	4,32787193	1,60224913	0,37021639	2,98564714	0
10457	H200017263	TNPO2	4,32541742	1,84951465	0,42759218	3,81083262	0
10877	H200018303	DOCK1	4,32533459	1,83105167	0,42333180	3,78813169	0
11220	H200019293	C12orf48	4,31916304	1,95256425	0,45207005	5,33013244	0
11741	H200020869	C1orf165	4,31505810	1,92788887	0,44678167	3,90114629	0
8436	H200012728	GEMIN4	4,31227488	2,18171143	0,50593051	4,70601447	0
435	H200000503	CCNF	4,30909985	1,73844967	0,40343685	3,36391348	0
2241	H200002822	ASB9	4,30819941	1,26765861	0,29424325	2,31868489	0
7033	H200010210	C1orf113	4,30611685	1,08917793	0,25293738	2,12512820	0
7611	H200011200	TKTL1	4,30395934	2,10617950	0,48935860	4,66151409	0
4356	H200005892	HLA-DQB1	4,30377902	1,99424307	0,46337023	4,72771474	0
2407	H200003052	TMEM55A	4,30374985	1,29471406	0,30083395	2,34821984	0
7923	H200011701	PFAAP5	4,30013807	1,79864512	0,41827613	3,71344730	0
10698	H200017796	GLT8D1	4,29864748	1,20280164	0,27980932	2,24009965	0
2487	H200003161	MYO6	4,29694589	1,69549620	0,39458170	3,32987746	0
6809	H200009698	KIAA1026	4,29603593	2,09047476	0,48660551	4,53036108	0
9317	H200014505	UGT1A6	4,29599462	1,83601396	0,42737809	3,96927448	0
9312	H200014486	ZNF34	4,29596857	1,67374365	0,38960798	3,37500149	0
11305	H200019484	DYNC1H1	4,29456891	2,02041002	0,47045700	4,58530950	0
10024	H200016194	CBX8	4,29450964	1,59223138	0,37075976	3,25093041	0
9977	H200015946	OR2W1	4,29278114	2,02890737	0,47263238	4,49316575	0
3283	H200004265	AS3MT	4,29193382	2,00189239	0,46643133	3,70919167	0
7392	H200010797	C6orf105	4,29114382	1,30995005	0,30526827	2,52412664	0
655	H200000773	FAM63A	4,28597737	1,10540007	0,25791085	2,14197452	0
9763	H200015491	DNER	4,28353945	1,69986412	0,39683634	3,35645083	0
10026	H200016201	WDR76	4,28313866	1,26628284	0,29564367	2,38786001	0
4328	H200005847	EYA4	4,28001333	1,53723089	0,35916498	2,88287809	0
4342	H200005866	GNAI3	4,27911694	2,05617996	0,48051502	4,11634629	0
1267	H200001517	ERBB4	4,27655019	2,03348700	0,47549705	4,28883994	0
6827	H200009762	HS2ST1	4,27450502	1,83222988	0,42864142	3,73236839	0
11276	H200019412	AKR7A2	4,27434111	2,14941676	0,50286505	4,82922561	0
10065	H200016258	RSF1	4,26712354	1,85385558	0,43445088	3,88180416	0
11123	H200019079	SMURF2	4,26587546	2,51559810	0,58970266	10,45250858	0
9819	H200015588	IGSF4	4,26469311	2,31139344	0,54198353	6,74466428	0
8359	H200012544	GHR	4,26015591	2,01033200	0,47189165	4,25861447	0
10861	H200018234	SLC30A5	4,25770161	1,84364410	0,43301393	3,67960443	0
9941	H200015837	ELOVL2	4,25441165	1,99246575	0,46832933	4,23347185	0
4946	H200006615	DHPS	4,25431490	1,32944043	0,31249225	2,36625101	0
7040	H200010225	FOXI1	4,25293216	1,55653331	0,36599063	2,89875391	0
4065	H200005420	OSBPL11	4,25037124	1,45319554	0,34189850	2,75995648	0
9205	H200014281	CAMK1	4,24576373	2,44099724	0,57492536	5,86684296	0
11097	H200019038	ATG9A	4,24564343	1,83305974	0,43175075	3,15569794	0
8747	H200013458	PGBD1	4,23809223	1,70774529	0,40295142	3,48457018	0
1650	H200002014	ANKRD44	4,23190572	2,07071543	0,48931039	4,26730131	0
3174	H200004093	ELMO1	4,22701455	1,39765464	0,33064817	2,71116458	0
11550	H200020157	SYNCRIP	4,22632259	1,71624662	0,40608510	3,63735424	0
10832	H200018183	CTNNB1	4,22310819	1,81448167	0,42965550	3,63436473	0
2515	H200003195	TMOD3	4,20945350	1,77618559	0,42195159	3,48351635	0
11784	H200021032	DENND1B	4,20855753	1,84443763	0,43825886	3,87871399	0
3855	H200005103	DGKE	4,20637184	1,42904844	0,33973422	2,69677521	0
11177	H200019159	SULT1A4	4,20359098	1,14791709	0,27308011	2,22150526	0
4425	H200005979	AP2B1	4,20243913	1,69028817	0,40221598	3,21238403	0
10894	H200018354	HMGCLL1	4,20210447	1,81918866	0,43292324	3,78615240	0
6492	H200009016	FNDC3B	4,20169375	1,83339443	0,43634652	3,76001752	0
9855	H200015644	GDA	4,20008490	1,23270780	0,29349593	2,41611302	0
6602	H200009320	OTX2	4,19991567	1,74856404	0,41633313	3,28892785	0

11738	H200020864	C6orf142	4,19736410	2,00820714	0,47844483	4,67889030	0
10743	H200017926	SLC38A2	4,19182730	1,21428049	0,28967808	2,36789982	0
8116	H200012059	CCBP2	4,19101575	2,04546171	0,48805870	4,08854078	0
5815	H200007920	C14orf100	4,19009018	1,83701887	0,43841989	3,35309896	0
2467	H200003135	LOC56757	4,18798533	1,74888182	0,41759502	3,48787961	0
9557	H200015021	GREM2	4,18755476	1,91104780	0,45636366	3,90657310	0
2924	H200003764	KIAA0984	4,18514620	1,93081128	0,46134859	4,08216069	0
8356	H200012540	HPS6	4,18327812	1,31264490	0,31378380	2,37389920	0
9249	H200014349	DEGS1	4,18123304	1,57871986	0,37757280	2,90395523	0
10391	H200017103	SLC43A3	4,17962667	1,35715535	0,32470731	2,70031795	0
9922	H200015781	LY6G6C	4,17681629	1,75009443	0,41900201	3,72109249	0
8576	H200013046	ZIC2	4,16723221	1,77530508	0,42601540	3,13880345	0
8039	H200011910	FZD6	4,16374793	1,28198515	0,30789211	2,32242957	0
11654	H200020472	PRKCB1	4,15975707	1,10205101	0,26493158	2,14624443	0
5353	H200007111	TMEM56	4,15975462	1,78755361	0,42972574	3,62996442	0
8454	H200012779	CHIA	4,15877985	2,26577655	0,54481762	7,44189322	0
3251	H200004213	NOVA2	4,15799971	2,11824875	0,50943937	5,18279437	0
11293	H200019450	MNT	4,15690880	1,57624772	0,37918747	3,18990866	0
5844	H200007970	EFHC2	4,15614402	1,71701061	0,41312587	3,42611223	0
11249	H200019358	RAB6A	4,15225489	1,57476564	0,37925553	3,33969052	0
10991	H200018770	RASGEF1B	4,14845798	1,96216627	0,47298690	3,94535309	0
1249	H200001497	PNMA2	4,14449780	1,95257713	0,47112515	3,99458181	0
2174	H200002736	GLYAT	4,14391432	1,76300990	0,42544555	3,54660698	0
2306	H200002914	IGFBP6	4,13870429	1,37580771	0,33242474	2,47982015	0
9460	H200014802	PTPRR	4,13723336	2,17682614	0,52615503	5,07287731	0
8989	H200013921	TRAM2	4,13433820	1,11231427	0,26904288	2,18619462	0
10784	H200018030	CCRL2	4,13337957	1,94895716	0,47151662	4,05011838	0
5718	H200007774	GALNT5	4,13199482	1,55856808	0,37719507	2,97735072	0
8981	H200013912	SMAD6	4,13167278	1,96108908	0,47464772	3,81983751	0
3142	H200004054	NHLH1	4,13162258	1,84141572	0,44568827	4,22796182	0
8177	H200012145	KIAA0408	4,13108326	1,96948304	0,47674736	4,72865125	0
5836	H200007959	VGF	4,13035816	1,37119147	0,33197883	2,66725471	0
9673	H200015292	BRWD1	4,13018787	1,72496108	0,41764712	3,47063357	0
17	H200000016	PIGA	4,12943355	1,31210039	0,31774343	2,65832772	0
8996	H200013931	MLANA	4,12775553	1,94408577	0,47097890	4,04791182	0
11099	H200019042	FAM54B	4,12719269	1,67307420	0,40537826	3,03151360	0
9937	H200015823	TIMP3	4,12683081	1,23658094	0,29964421	2,39528758	0
5468	H200007289	PTPRD	4,12641150	1,85871003	0,45044224	3,62554827	0
2933	H200003778	C3orf29	4,12589553	1,60572974	0,38918332	2,99009454	0
2287	H200002890	TFIP11	4,11723199	1,89408973	0,46003959	3,82378171	0
9704	H200015370	CDIPT	4,11720497	1,07314338	0,26064852	2,04231051	0
9134	H200014154	ZNF132	4,11622211	2,08418725	0,50633498	4,32463061	0
9857	H200015646	KCTD3	4,11262458	0,99902551	0,24291678	2,02465043	0
765	H200000896	VAV2	4,11183892	1,31529842	0,31988082	2,62364504	0
9151	H200014189	STATH	4,10976066	2,36013820	0,57427631	5,22796647	0
9479	H200014833	JUNB	4,10512095	1,38085747	0,33637437	2,60802344	0
7153	H200010404	PDE4A	4,10455054	2,52358675	0,61482658	6,11099077	0
9544	H200014991	MTMR1	4,10437159	1,79940011	0,43841062	3,69728170	0
64	H200000071	POU4F2	4,10379934	1,55146411	0,37805555	3,23748093	0
3814	H200005040	CD5L	4,09849583	1,86515864	0,45508370	3,81179384	0
5252	H200006990	C1orf119	4,09569626	1,73014959	0,42243113	3,32165547	0
10746	H200017936	MSRA	4,08963045	1,77271516	0,43346586	3,70046317	0
4058	H200005409	CASQ1	4,08828071	1,80618625	0,44179604	3,55571496	0
10899	H200018377	ZNF287	4,08597640	1,77244937	0,43378845	3,65417022	0
6485	H200008978	MAGI1	4,08347395	2,06890321	0,50665273	4,24863956	0
4732	H200006362	SPTAN1	4,08024417	1,13568602	0,27833776	2,11906056	0
6789	H200009664	FSTL1	4,07964020	1,04666646	0,25655852	2,04003469	0
11258	H200019382	PLA2G12B	4,07952701	1,88621317	0,46236075	4,11702424	0
4105	H200005485	PAK1	4,07376510	1,82901215	0,44897339	3,80455584	0
6917	H200009983	RERE	4,06986262	2,00293998	0,49213946	4,46594476	0
4405	H200005953	ATXN1	4,06373564	1,64977582	0,40597518	3,39415472	0
9831	H200015602	ZNF187	4,06319938	1,10499548	0,27195207	2,18125706	0
4138	H200005540	RIMBP2	4,06153544	1,94056503	0,47779099	3,75627871	0
9514	H200014914	AKR1D1	4,06064934	2,01742133	0,49682234	4,27812043	0
5230	H200006967	CD47	4,05682846	1,25351032	0,30898776	2,35247945	0
3313	H200004314	MBD4	4,05494287	1,64643075	0,40603057	3,58413584	0
4151	H200005554	FBXO28	4,05481936	1,62604268	0,40101483	3,14852605	0
1208	H200001447	LOC728558	4,05440756	1,31258945	0,32374384	2,40553795	0
3712	H200004897	AKAP5	4,05379547	1,19691697	0,29525835	2,17243296	0
2863	H200003673	CNOT8	4,05077507	1,83528100	0,45306910	3,74821813	0
11046	H200018932	PTCHD1	4,05048512	2,08435682	0,51459436	6,29285174	0
11869	H200021278	SMC2	4,04776276	1,15159430	0,28450143	2,15009591	0
10875	H200018296	C16orf68	4,04657319	1,91675910	0,47367464	4,09756004	0
11425	H200019834	RAD50	4,04602583	1,84868747	0,45691440	5,00927551	0
11728	H200020828	ACAD11	4,04539684	1,95825210	0,48406922	4,02704248	0
4926	H200006595	HDHD1A	4,04323417	1,03247744	0,25535930	2,06634726	0
9253	H200014359	ZNF687	4,04276745	1,94114804	0,48015328	3,70258775	0
4203	H200005634	HOXA1	4,04056274	1,73299232	0,42889875	3,48268717	0
5561	H200007492	SMARCB1	4,03419636	1,53303314	0,38000955	2,72219327	0
8503	H200012899	ATP8A2	4,03372680	2,36450296	0,58618322	8,41284716	0
118	H200000136	AFM	4,03323650	1,14077877	0,28284450	2,21755448	0
3515	H200004606	APH1B	4,03227658	1,62053726	0,40189139	3,22978461	0
9678	H200015303	CCR9	4,03180501	1,94753303	0,48304247	4,19421818	0
8500	H200012892	CCNB3	4,03076316	1,46261637	0,36286339	2,87152895	0
1423	H200001728	TMEM50B	4,02867590	1,32956755	0,33002594	2,43700893	0
5826	H200007939	PCSK6	4,02502389	2,39407139	0,59479681	4,58837915	0
1824	H200002261	LANCL1	4,02281224	1,04077085	0,25871723	2,08131200	0
4046	H200005386	CCDC77	4,01804384	1,30831307	0,32560946	2,33232601	0
7832	H200011569	UBE2D2	4,01527682	1,88498540	0,46945341	4,20361573	0
4610	H200006215	EID1	4,01524229	1,16993129	0,29137253	2,31029407	0
3693	H200004868	NEK1	4,00774428	1,14299665	0,28519700	2,16635074	0

5035	H200006722	SEPT10.	4,00693632	1,88894110	0,47141780	3,51318432	0
767	H200000898	LOC284244	4,00526369	1,05810894	0,26417960	2,07308418	0
3990	H200005299	AKAP9	4,00519336	1,38134751	0,34488909	2,53177805	0
9663	H200015259	JAG1	4,00265957	1,75494944	0,43844584	3,53581975	0
11766	H200020985	SPATA22	4,00110502	1,71397299	0,42837491	3,50049260	0
11698	H200020670	C1orf125	4,00079768	2,34374497	0,58581942	8,87854773	0
3565	H200004677	GPR126	3,99963768	1,21344524	0,30338879	2,40387247	0
8848	H200013706	POM121	3,99897398	2,23800747	0,55964542	6,92718793	0
7974	H200011771	MYO5C	3,99435998	1,64937462	0,41292588	3,07948691	0
5829	H200007945	DIRC1	3,99135110	1,82939645	0,45834015	3,51404474	0
10147	H200016458	WNT2B	3,99116586	1,46783527	0,36777105	2,96098794	0
8164	H200012130	HK1	3,98965440	1,37660436	0,34504351	2,35797385	0
8066	H200011954	C20orf179	3,98912090	1,78292177	0,44694603	3,76888204	0
11849	H200021214	KRTAP4-7	3,98910128	1,38683639	0,34765635	2,61151322	0
10025	H200016200	MYADML	3,98902851	1,86457168	0,46742501	4,06204370	0
5144	H200006858	GRB10	3,98742669	1,36185527	0,34153738	2,41739180	0
2623	H200003350	C1orf124	3,98706035	1,66848727	0,41847555	3,31278590	0
10347	H200016962	DUOX1	3,98692587	1,35482728	0,33981753	2,28578909	0
6863	H200009867	AP3M1	3,98504240	1,74111896	0,43691353	3,06692502	0
10230	H200016610	PPP1R2	3,98495218	1,04316579	0,26177624	2,07024502	0
7424	H200010877	PRSS35	3,98169566	1,57904906	0,39657703	3,00423930	0
5593	H200007568	NTNG2	3,98122960	1,74478918	0,43825384	3,48536344	0
7395	H200010812	PLCZ1	3,98107711	1,87971603	0,47216268	3,74628342	0
7037	H200010217	LOC283658	3,98079385	1,75630149	0,44119378	3,61288649	0
3768	H200004973	INTS2	3,97809845	1,17272488	0,29479534	2,24734249	0
10898	H200018371	SEPT11.	3,97229264	1,56513112	0,39401204	2,91966775	0
7000	H200010153	LOC389541	3,97122887	1,29802097	0,32685625	2,49521681	0
10956	H200018589	THRAP2	3,97018875	1,66780804	0,42008281	3,48560259	0
11179	H200019161	AMMECR1	3,97010460	1,04230471	0,26253835	2,10226788	0
11886	H200021313	CAPN1	3,96822435	1,59375088	0,40162822	3,32980709	0
10107	H200016364	DSCR6	3,96367584	1,86354673	0,47015619	3,52666934	0
8713	H200013381	PNLIPRP2	3,96165353	1,83090509	0,46215679	4,18894071	0
11004	H200018831	FLJ30672	3,96114675	1,58618069	0,40043472	3,17447989	0
8242	H200012256	FLJ90709	3,96074159	1,44180652	0,36402438	2,68522542	0
10299	H200016772	MAPK6	3,95940749	1,55811641	0,39352262	2,89305895	0
2786	H200003570	SEMA4F	3,95439277	1,37452311	0,34759398	2,62445819	0
3523	H200004617	ZNF623	3,95350808	1,72703707	0,43683661	3,50402581	0
9607	H200015137	CUGBP2	3,95066022	1,44360793	0,36540929	3,17711795	0
6517	H200009104	EEF1G	3,95061162	1,56338961	0,39573356	3,04316273	0
11197	H200019205	PNLIPRP1	3,94839320	1,83430384	0,46456970	3,57335353	0
4590	H200006193	NR3C1	3,94375715	1,65431429	0,41947671	3,26990129	0
6323	H200008641	STRN3	3,94275149	1,77901854	0,45121244	3,09741391	0
9778	H200015515	LPO	3,94079562	1,77335180	0,44999842	3,38006935	0
7759	H200011466	LOC285550	3,93551869	1,49339522	0,37946592	2,82107139	0
10841	H200018202	CDK5RAP1	3,93348940	1,05610892	0,26849161	2,10196095	0
7695	H200011358	RNF166	3,93143673	1,27817745	0,32511713	2,45971062	0
2378	H200003011	SMARCAD1	3,93095534	1,69643936	0,43155905	2,69773126	0
8292	H200012381	C1orf114	3,92850566	1,81228923	0,46131771	3,78964490	0
5560	H200007491	OR5I1	3,92539427	1,39251527	0,35474533	2,68510601	0
8628	H200013172	KIAA0773	3,92535595	1,69344336	0,43141141	3,38148882	0
10216	H200016593	MUM1	3,92441188	1,39751805	0,35610891	2,63279496	0
5553	H200007475	LY6H	3,92192428	1,88260259	0,48002013	3,88723571	0
11593	H200020326	LOC642426	3,92121320	1,58592408	0,40444730	3,24716810	0
8074	H200011972	HTRA2	3,91863443	1,54601356	0,39452865	3,14754453	0
2801	H200003592	TNRC4	3,91764664	1,79115238	0,45720111	3,62424619	0
529	H200000620	LARS2	3,91696430	1,82056044	0,46478862	5,06084363	0
5653	H200007675	CUBN	3,91260443	1,79148690	0,45787581	4,10603042	0
9994	H200016068	GPR22	3,90461210	1,73144009	0,44343460	3,59682731	0
7698	H200011364	RAD54L2	3,89941835	1,10578730	0,28357750	2,09426078	0
3851	H200005096	AFF2	3,89739761	1,82224740	0,46755491	3,84141768	0
11227	H200019314	TMEM163	3,89721318	1,58270194	0,40611120	3,43922948	0
9863	H200015655	PIK3C3	3,89716315	1,65829510	0,42551339	3,58141426	0
3052	H200003935	RBBP6	3,89349492	1,18565468	0,30452195	2,33776269	0
2572	H200003272	APOLD1	3,89233182	1,77978438	0,45725402	3,48831855	0
6505	H200009057	KIAA1240	3,88935967	1,89564609	0,48739285	4,39207192	0
9328	H200014534	TTC9B	3,88834550	1,83602286	0,47218614	4,05962252	0
10130	H200016424	DBT	3,88594227	1,51389985	0,38958372	2,92550038	0
11291	H200019448	DDEF2	3,88281372	1,83649636	0,47298080	3,66323275	0
6875	H200009892	GTF2A1	3,88262008	1,74512583	0,44947118	3,06516303	0
10522	H200017426	TFEC	3,87980931	1,91537725	0,49367819	3,82610583	0
5127	H200006838	THOC2	3,87850053	1,04822751	0,27026618	2,06235213	0
473	H200000552	CSF3R	3,87687134	1,79842486	0,46388562	3,61328408	0
8679	H200013302	PDE5A	3,87333366	1,84356005	0,47596211	3,67648205	0
6821	H200009744	DDEF1	3,87266100	1,85687126	0,47948200	3,61122096	0
10021	H200016180	UIMC1	3,87160421	1,75229222	0,45260107	3,47221740	0
7384	H200010774	HSF5	3,87125529	1,93736223	0,50044807	3,99977431	0
8433	H200012723	RP11-393H1(	3,86996368	1,82094078	0,47053175	3,62296464	0
9683	H200015319	NCOA3	3,86952416	1,83910519	0,47527942	3,18683426	0
4347	H200005880	CPN2	3,86734217	1,82860469	0,47283240	3,69335889	0
5434	H200007238	ZNF295	3,86721916	1,17651202	0,30422688	2,22892678	0
4137	H200005539	KIAA0427	3,86420630	1,64207666	0,42494539	3,25690269	0
9189	H200014257	C2orf3	3,86277334	1,34674569	0,34864735	2,82351745	0
910	H200001070	WDR26	3,85421188	1,29882158	0,33698759	2,67146275	0
5507	H200007368	CLSTN2	3,85121144	1,20107516	0,31186944	2,28762190	0
1988	H200002491	DUSP21	3,85119838	1,89463919	0,49196094	3,74344270	0
9642	H200015212	GRWD1	3,85106131	1,42659191	0,37044124	2,77229051	0
10010	H200016153	FA2H	3,85068215	1,13096723	0,29370568	2,09159598	0
6938	H200010025	ZMYND11	3,85046617	1,28604129	0,33399626	2,48955330	0
8320	H200012469	SLC6A11	3,84979035	1,75252844	0,45522698	3,26918213	0
7564	H200011125	MTA1	3,84905010	1,35503925	0,35204511	2,51817141	0
8912	H200013812	C4orf16	3,84803543	1,42597191	0,37057141	2,58784194	0

11792	H200021056	PSMD6	3,84450344	1,70359642	0,44312522	3,29181718	0
10367	H200016989	USP52	3,84266638	1,84356532	0,47976200	3,70767404	0
7713	H200011384	LINS1	3,84209643	1,10591377	0,28784123	2,15972340	0
10932	H200018542	RAB33A	3,83770997	1,88534583	0,49126845	3,88714184	0
2537	H200003223	POFUT2	3,83727473	0,99310745	0,25880541	2,01502842	0
11788	H200021046	C14orf32	3,83428311	1,38793153	0,36197941	2,88664448	0
7284	H200010600	ZNF608	3,83184007	1,53343101	0,40018137	3,08492481	0
11236	H200019330	KCTD20	3,82924415	1,12962231	0,29499877	2,30552688	0
7606	H200011192	WDR40A	3,82039284	1,25077362	0,32739398	2,29357263	0
11012	H200018856	TEX10	3,81942035	1,92838215	0,50488869	3,86669726	0
9805	H200015565	CHST6	3,81933359	1,30228053	0,34097062	2,45943825	0
7486	H200010994	C10orf22	3,81883450	1,03222937	0,27029958	2,13583372	0
967	H200001139	FEM1B	3,81754544	1,26168670	0,33049684	2,39971944	0
6608	H200009336	EDD1	3,81641974	1,96385595	0,51458070	4,21746250	0
10173	H200016517	SAMD4A	3,80994679	1,74479363	0,45795748	3,02599273	0
6641	H200009394	ELMOD2	3,80640111	1,75774270	0,46178599	3,48871421	0
3498	H200004579	SLC25A36	3,80437669	1,05876678	0,27830230	2,03793847	0
6220	H200008487	VPS41	3,80408520	1,70937681	0,44935292	3,47971224	0
10179	H200016529	HAL	3,80321851	1,74408232	0,45858062	3,56740980	0
7051	H200010246	ALDH3B2	3,79554994	1,86388063	0,49106998	4,77413582	0
1378	H200001675	STK38	3,79486651	1,06129817	0,27966680	2,08283402	0
2868	H200003683	FABP7	3,78795731	2,36384929	0,62404328	14,02185566	0
11760	H200020973	UCHL1	3,78465599	2,12669033	0,56192434	9,70264238	0
9096	H200014083	PSMD7	3,78179082	1,23255717	0,32591892	2,41309854	0
4330	H200005850	LY6G5B	3,78155214	1,51079539	0,39951727	2,78973399	0
10106	H200016363	LOC728434	3,77724871	1,66550175	0,44092986	3,43732301 a	
11388	H200019700	POLDIP3	3,77627009	1,00511324	0,26616561	2,08221171	0
11403	H200019751	CEP57	3,77529582	1,74627364	0,46255280	3,53886170	0
5350	H200007108	SNAP25	3,77218605	1,69083967	0,44823867	3,32054282	0
10650	H200017635	SLC24A2	3,77197712	1,79568539	0,47605946	3,73470771	0
2964	H200003815	UBR1	3,77164130	1,19506297	0,31685489	2,35239135	0
4528	H200006098	UBAP1	3,77108657	1,07900570	0,28612594	2,09239577	0
5219	H200006951	ST8SIA1	3,77027252	1,84125027	0,48835999	3,82039156	0
11798	H200021068	PSIP1	3,76916471	1,46647886	0,38907264	2,84481545	0
3739	H200004932	FAM119B	3,76427323	1,07295366	0,28503607	2,02835512	0
5640	H200007650	RUNX1	3,69049637	1,81595911	0,49206365	3,76025760	0
8251	H200012272	STK17B	3,62451868	1,36699912	0,37715328	2,71845569	0
5407	H200007183	GML	3,40985322	1,51999454	0,44576539	3,12859276	0
4974	H200006648	RGL1	3,18546393	1,21145838	0,38030830	2,29380907	0
10867	H200018256	ESR1	3,04017078	1,81610763	0,59737027	4,44686693	0

Negative genes (614)

Row	Gene ID	Gene Name	Score(d)	Numerator(r)	Denominator(s+s0)	Fold Change	q-value(%)
2597	H200003310	LRP6	-8,61020309	-1,89295597	0,219850327	0,270538996	0
4303	H200005804	CDKN2B	-7,77238232	-1,88879996	0,243014289	0,275993679	0
2935	H200003780	MYST4	-7,61701068	-1,48932801	0,195526575	0,353860938	0
9065	H200014045	ZNF174	-7,59178518	-1,90849001	0,251388832	0,263111011	0
5378	H200007142	MLF1	-7,49187092	-1,65360993	0,220720558	0,314397264	0
3033	H200003912	CPT1B	-7,43720698	-1,67731926	0,225530803	0,317361251	0
6259	H200008544	TARSL2	-7,39519091	-1,44539507	0,195450677	0,363829890	0
5084	H200006783	UCP2	-7,29549556	-2,03111189	0,278406295	0,254683570	0
3257	H200004222	KIAA1160	-7,28080445	-1,66659139	0,228902094	0,315446277	0
7386	H200010779	RAB27B	-7,20301383	-2,39840249	0,332972078	0,185398851	0
5193	H200006915	MYO1E	-7,20072623	-1,65783091	0,230231070	0,318103210	0
11211	H200019263	GPR61	-7,15093242	-1,97247557	0,275834738	0,260791400	0
8274	H200012344	ASCC1	-7,11455669	-1,80405047	0,253571733	0,287957748	0
6745	H200009587	ZNF436	-7,04969471	-1,23432922	0,175089741	0,425254266	0
6074	H200008285	ZSCAN5	-7,00250300	-1,61777434	0,231028010	0,330376621	0
9039	H200014006	CPT1B	-7,00033213	-1,89303456	0,270420678	0,276495762	0
8804	H200013605	DNAI2	-6,99766167	-1,53042650	0,218705416	0,350538935	0
7171	H200010431	NEO1	-6,99644512	-1,55141440	0,221743238	0,343978201	0
9610	H200015141	CHES1	-6,97302094	-1,51648884	0,217479462	0,351950439	0
10158	H200016490	TRO	-6,95997920	-1,73431624	0,249184113	0,301483822	0
9318	H200014508	ZNF334	-6,91453563	-1,92786474	0,278813335	0,271760983	0
10423	H200017204	KATNB1	-6,90298610	-1,87130465	0,271086255	0,282681667	0
10450	H200017254	HEXDC	-6,84552121	-1,86320369	0,272178500	0,283370811	0
3330	H200004339	GKAP1	-6,83243849	-1,52114204	0,222635306	0,354236091	0
9917	H200015773	DNMT3A	-6,80838201	-1,46998268	0,215907785	0,361785135	0
7006	H200010166	HIST2H4A	-6,78007951	-1,96057475	0,289166926	0,251469188	0
7700	H200011368	GMDS	-6,74577531	-2,13213017	0,316068958	0,245466717	0
7269	H200010581	U1SNRNPBP	-6,73648871	-1,58995944	0,236021985	0,335416188	0
5175	H200006892	LAMB1	-6,72731398	-1,54612154	0,229827468	0,342736925	0
11552	H200020190	FAM62B	-6,70278697	-1,71101204	0,255268748	0,304207325	0
8403	H200012647	KCNK5	-6,65676080	-1,43059478	0,214908545	0,369154801	0
8493	H200012876	HYAL1	-6,65517966	-1,93261330	0,290392355	0,271332928	0
7545	H200011081	RAD51L1	-6,63868196	-1,51813059	0,228679518	0,352390672	0
8050	H200011928	LTB4DH	-6,59358913	-2,11017813	0,320034824	0,223979493	0
6407	H200008797	DUS3L	-6,57452781	-1,86995337	0,284423981	0,283796434	0
5642	H200007653	CDK5	-6,57245778	-1,77465904	0,270014521	0,290777708	0
4560	H200006145	MARCKS	-6,57228534	-1,89482288	0,288305023	0,269107627	0
8706	H200013366	C5orf13	-6,57004940	-1,92495529	0,292989470	0,265514638	0
9608	H200015139	CLCN6	-6,56702196	-1,82369341	0,277704783	0,281660954	0
8092	H200012007	ARHGEF10L	-6,54194289	-1,83178355	0,280006045	0,290364857	0
6377	H200008740	ANKFY1	-6,52950469	-1,51019587	0,231287967	0,354428229	0
7654	H200011282	GABRB2	-6,52765816	-1,45441337	0,222807834	0,366993148	0
5165	H200006881	IL6ST	-6,52042633	-1,70530509	0,261532759	0,303908603	0
8727	H200013412	WDR4	-6,51007196	-1,37635949	0,211420011	0,388484591	0
2607	H200003328	HEYL	-6,48231377	-1,72210561	0,265662180	0,298680832	0
8987	H200013919	TNK2	-6,47682945	-1,20569307	0,186154827	0,432003855	0

9226	H200014309	PCGF2	-6,46611377	-1,84938810	0,286012304	0,289307948	0
1168	H200001399	CORO7	-6,43744277	-1,56202188	0,242646333	0,338807571	0
5879	H200008013	TRIO	-6,39925493	-1,61784580	0,252817838	0,334717780	0
8534	H200012965	SUSD2	-6,39363003	-1,90323410	0,297676608	0,257609472	0
2862	H200003671	CAPS	-6,37694789	-1,70589473	0,267509593	0,314701225	0
4367	H200005906	NCOA1	-6,37100064	-1,60694653	0,252228279	0,330464903	0
3780	H200004990	LIMD2	-6,37019736	-1,44169698	0,226319045	0,370517316	0
6842	H200009824	ET	-6,36942573	-1,99126035	0,312627925	0,266727710	0
9064	H200014044	TCEB3	-6,36621036	-1,88563285	0,296193928	0,270347093	0
7085	H200010301	PNOC	-6,35735928	-2,10911535	0,331759660	0,243132222	0
8800	H200013588	CUTL1	-6,34268819	-1,53873952	0,242600531	0,335766557	0
9841	H200015617	C6orf96	-6,32999333	-1,86874673	0,295220964	0,281371294	0
9393	H200014672	BAG4	-6,32910118	-1,54877433	0,244706837	0,343286232	0
3709	H200004892	LILRA4	-6,30805201	-1,70511954	0,270308414	0,318493413	0
7488	H200010996	PINX1	-6,30481483	-1,82034508	0,288723004	0,292713693	0
8972	H200013900	GPR98	-6,30434785	-2,18579849	0,346712863	0,208472171	0
2304	H200002912	MRE11A	-6,29902637	-1,64400812	0,260994005	0,327057534	0
9119	H200014120	INPP5D	-6,29201213	-1,76043416	0,279788742	0,301056348	0
4883	H200006548	ZNF32	-6,25403434	-1,58231383	0,253006899	0,340050420	0
10188	H200016546	FKBP14	-6,25372202	-1,99737587	0,319389935	0,254971866	0
6254	H200008538	C9orf46	-6,23714919	-1,68999425	0,270956201	0,317155722	0
7436	H200010914	DDX25	-6,22437280	-1,90722082	0,306411728	0,272478753	0
797	H200000939	SCN3B	-6,21449451	-1,52281514	0,245042478	0,352878063	0
11149	H200019112	ZNF747	-6,21421026	-1,79170249	0,288323442	0,283542946	0
9923	H200015782	LY6G6D	-6,20331814	-1,54838588	0,249606072	0,349445540	0
7860	H200011607	OSGEP	-6,18067400	-1,78662223	0,289065923	0,300501882	0
3609	H200004740	TAS2R49	-6,17930451	-1,94302525	0,314440767	0,259234026	0
5119	H200006826	RAC1	-6,16145531	-1,82249584	0,295789833	0,282453697	0
6272	H200008568	POU2F1	-6,14921332	-1,62161595	0,263711123	0,334859131	0
7657	H200011286	MEIS2	-6,14742577	-1,52644921	0,248307059	0,354833430	0
5805	H200007905	CHML	-6,14616772	-1,45826443	0,237264014	0,368581728	0
5314	H200007064	IRF2	-6,13302094	-1,71445721	0,279545306	0,316465712	0
10596	H200017548	ZNF232	-6,12782652	-1,73710668	0,283478436	0,289471875	0
10574	H200017518	BCCIP	-6,12678352	-2,00032871	0,326489210	0,267199476	0
11669	H200020502	C22orf25	-6,12408138	-1,43952255	0,235059344	0,370149665	0
6553	H200009214	ZNF703	-6,12253065	-1,50399996	0,245650049	0,347229118	0
4082	H200005455	SMARCE1	-6,11330715	-1,19257471	0,195078488	0,433089132	0
8183	H200012154	ENO3	-6,10617365	-1,79348249	0,293716261	0,299464238	0
1450	H200001762	ELN	-6,10483331	-1,43547972	0,235138233	0,377023388	0
6794	H200009671	SGK	-6,10061207	-1,53251048	0,251206021	0,350955695	0
3838	H200005073	BARD1	-6,09976472	-1,65946775	0,272054387	0,327088742	0
2233	H200002812	SIRT3	-6,09394577	-1,85177758	0,303871687	0,291093623	0
11446	H200019895	RSNL2	-6,08563106	-1,42265856	0,233773383	0,372010200	0
7043	H200010228	HRK	-6,07411837	-1,53646746	0,252953164	0,346518887	0
3974	H200005270	GIT2	-6,07344126	-1,73256672	0,285269363	0,312064732	0
9938	H200015826	CCDC131	-6,07037488	-1,29467704	0,213277939	0,412317218	0
3766	H200004970	NDN	-6,06983463	-1,49170523	0,245757144	0,353847188	0
9701	H200015362	C20orf121	-6,04951477	-1,41058955	0,233173999	0,382171018	0
5800	H200007898	VCY	-6,04891463	-1,89190095	0,312767012	0,284083110	0
6910	H200009963	ZNF629	-6,04803198	-1,80433986	0,298335040	0,291254449	0
11337	H200019569	SNX21	-6,02052843	-1,59981157	0,265726104	0,341381930	0
3946	H200005228	LSP1	-6,02006680	-1,34562896	0,223523925	0,396332704	0
10283	H200016731	EXOC7	-6,01031777	-2,02151160	0,336340220	0,242286481	0
10264	H200016684	KRR1	-6,00943839	-1,96098798	0,326318010	0,240113111	0
8514	H200012916	ABI3	-5,99754128	-2,09523464	0,349348931	0,254864022	0
10728	H200017894	PHF20L1	-5,99617851	-1,61202232	0,268841616	0,334371277	0
3715	H200004903	ZADH2	-5,98839930	-1,47695845	0,246636600	0,360702449	0
1309	H200001580	PBXIP1	-5,98736322	-1,69374274	0,282886253	0,318831182	0
3423	H200004477	LRMP	-5,98602777	-1,27719100	0,213362024	0,414859549	0
5904	H200008042	SUV420H2	-5,98397972	-1,72479145	0,288234843	0,315442388	0
6418	H200008839	MLXIPL	-5,98014351	-1,45697536	0,243635518	0,355139745	0
4314	H200005825	PRB3	-5,97548765	-1,84902262	0,309434599	0,282014996	0
9114	H200014110	USP32	-5,96904557	-1,59556138	0,267305946	0,340860429	0
8053	H200011932	AFF1	-5,96599585	-1,76778228	0,296309674	0,293542730	0
8682	H200013310	TTC23	-5,96444218	-1,70182569	0,285328559	0,321498579	0
5579	H200007538	UTS2	-5,96322596	-1,61004878	0,269996273	0,318932987	0
8010	H200011839	KIAA0892	-5,95769588	-1,41429146	0,237388999	0,381915515	0
8180	H200012150	ATF7IP	-5,94327119	-1,48345066	0,249601712	0,364447112	0
11572	H200020256	DR1	-5,94222505	-1,85898269	0,312842862	0,279922209	0
9754	H200015463	ZNF560	-5,93036072	-1,44802076	0,244170773	0,363462032	0
401	H200000463	PLP1	-5,92440961	-1,64354731	0,277419594	0,325317173	0
7487	H200010995	TLE4	-5,92425745	-1,89097392	0,319191719	0,286602856	0
1512	H200001844	FAM117A	-5,91793157	-1,36777039	0,231123049	0,393007006	0
8856	H200013716	C3orf21	-5,91153899	-1,72790767	0,292294050	0,316026595	0
9216	H200014297	LMO2	-5,86707681	-1,65889334	0,282746143	0,329069484	0
11159	H200019127	DYNC1LI2	-5,86494497	-1,47523206	0,251533829	0,342790666	0
8348	H200012525	DKFZp761E1	-5,85301321	-1,78028153	0,304164960	0,299138673	0
7253	H200010557	KIAA1414	-5,83812325	-1,73169452	0,296618356	0,316674272	0
10555	H200017484	RAB4B	-5,83799819	-1,54104340	0,263967777	0,336122466	0
3518	H200004611	HDGF2	-5,83662365	-1,54943635	0,265467921	0,347551357	0
2454	H200003117	C9orf37	-5,83261139	-1,34477266	0,230560991	0,398098834	0
11013	H200018859	MTHFS	-5,82357826	-1,77617241	0,304996745	0,295224412	0
1489	H200001811	PSMB10	-5,81116014	-1,32459248	0,227939422	0,405114557	0
2973	H200003828	WBP4	-5,80781051	-1,28421239	0,221118162	0,410453829	0
11695	H200020658	AP4B1	-5,79571262	-1,60945779	0,277697998	0,325115041	0
11883	H200021308	RAB28	-5,79394944	-1,62223696	0,279988112	0,319114430	0
8489	H200012872	FIZ1	-5,79114892	-1,34991154	0,233099090	0,396561470	0
2045	H200002557	RAB24	-5,78762173	-1,58712710	0,274227857	0,341368048	0
8267	H200012323	ARHGDIG	-5,77736792	-1,56635557	0,271119234	0,348702199	0
6766	H200009629	PBX3	-5,77694690	-1,19853409	0,207468428	0,438282485	0
6702	H200009502	MAML2	-5,77673494	-1,95470352	0,338375144	0,248341481	0

4054	H200005402	NAPG	-5,77467866	-1,36580989	0,236517038	0,394934529	0
5703	H200007755	IL15	-5,77035281	-1,52475250	0,264239043	0,346999347	0
6961	H200010066	KIAA1539	-5,75656265	-1,80693654	0,313891578	0,303376897	0
7517	H200011032	ACVR1B	-5,74491261	-1,31980407	0,229734403	0,403371493	0
3047	H200003930	C1orf96	-5,74386590	-1,39361501	0,242626662	0,375046450	0
4501	H200006065	MTM1	-5,74074591	-1,23560826	0,215234794	0,427649079	0
11525	H200020108	CCPG1	-5,73257345	-1,77173670	0,309064806	0,308909979	0
9822	H200015591	PGM3	-5,72445211	-1,33876288	0,233867426	0,393516523	0
11803	H200021082	DKFZp547E0	-5,72440428	-1,18135901	0,206372393	0,443605587	0
3969	H200005259	C6orf10	-5,71394927	-1,86602941	0,326574375	0,263319867	0
7732	H200011421	CDKN1C	-5,71221723	-1,66051302	0,290695005	0,329783111	0
7915	H200011691	CCDC101	-5,70738213	-1,75499001	0,307494744	0,296775263	0
2083	H200002606	MBIP	-5,70312336	-1,57590026	0,276322316	0,341470162	0
3016	H200003891	PURA	-5,69335387	-1,58999123	0,279271457	0,344786283	0
9428	H200014754	ZNF14	-5,69057829	-1,64030387	0,288249065	0,316659457	0
6976	H200010099	ZGPAT	-5,68693879	-1,24833025	0,219508296	0,416133115	0
1651	H200002015	CTSF	-5,68043746	-1,44710653	0,254752655	0,361959579	0
7192	H200010465	TRAF6	-5,67889573	-1,51521884	0,266815753	0,346483906	0
2323	H200002937	ZNF691	-5,67478930	-1,43836503	0,253465803	0,373059893	0
8766	H200013509	ASPM	-5,66018678	-1,35909714	0,240115246	0,383427170	0
170	H200000201	ITGA2B	-5,65588976	-1,34032778	0,236979120	0,397542786	0
3506	H200004593	MAX	-5,65459620	-1,39724449	0,247098898	0,386554747	0
7451	H200010949	SPATA17	-5,64721181	-1,21433537	0,215032729	0,435382383	0
8035	H200011896	GABRD	-5,64574872	-1,76251174	0,312183880	0,313310897	0
5118	H200006825	RAPSN	-5,63008533	-1,56904078	0,278688632	0,335344931	0
4349	H200005882	FAH	-5,62196878	-1,39921174	0,248882872	0,389125587	0
2087	H200002613	SMARCAL1	-5,61332000	-1,35584531	0,241540711	0,398771947	0
10494	H200017335	GCC2	-5,60793634	-1,40463833	0,250473302	0,375155015	0
3136	H200004046	SLC39A3	-5,60789331	-1,31287666	0,234112274	0,404428740	0
5133	H200006844	DECR1	-5,60312245	-1,57865781	0,281746084	0,339985132	0
6334	H200008656	ZNF268	-5,59943003	-1,58790003	0,283582440	0,336231074	0
7590	H200011171	MXD4	-5,59484902	-1,52341448	0,272288756	0,357973960	0
10117	H200016398	BIRC7	-5,58584435	-1,49781517	0,268144810	0,356618259	0
3538	H200004643	PTP4A3	-5,58513175	-1,41710845	0,253728741	0,381624723	0
4571	H200006166	MPV17	-5,58157002	-1,69820561	0,304252317	0,309693255	0
7321	H200010666	KCTD17	-5,57224628	-1,41823572	0,254517774	0,386662081	0
6427	H200008853	SLC35B3	-5,57109086	-1,74326185	0,312912120	0,302925550	0
11517	H200020094	C4orf36	-5,55929123	-1,54128138	0,277244224	0,343282268	0
11455	H200019910	CD2AP	-5,55788366	-1,46633381	0,263829526	0,361744969	0
11633	H200020438	MOCS1	-5,55722577	-1,31221834	0,236128311	0,404061096	0
8222	H200012212	STX8	-5,55689177	-1,56507717	0,281646150	0,337042100	0
8261	H200012288	LOC440330	-5,55170314	-1,32146578	0,238028898	0,409441529	0
2108	H200002645	ZNF79	-5,54158405	-1,53096228	0,276267989	0,352312432	0
3953	H200005240	LBH	-5,54134460	-1,50531852	0,271652212	0,363249181	0
4857	H200006518	NDST2	-5,53642885	-1,26291037	0,228109202	0,425076957	0
9998	H200016093	NKX6-1	-5,53523345	-1,67750835	0,303060091	0,326849211	0
11062	H200018979	TOMM40L	-5,52416054	-1,27826623	0,231395562	0,415890697	0
11777	H200021020	APTX	-5,52403507	-1,39521333	0,252571411	0,389211767	0
6241	H200008515	GABPB2	-5,51696124	-1,74796299	0,316834379	0,285196495	0
11193	H200019192	OR2A4	-5,51573990	-1,76477712	0,319952926	0,276473491	0
4701	H200006323	PPP1R1A	-5,51500265	-1,23755260	0,224397463	0,429688407	0
4675	H200006292	SLC38A3	-5,51402490	-1,33396774	0,241922691	0,403202329	0
3829	H200005058	CFP	-5,51159084	-1,49350449	0,270975211	0,367691004	0
9913	H200015765	RASA2	-5,51062489	-1,53604069	0,278741652	0,331658854	0
10551	H200017480	CHMP4A	-5,50701011	-1,51620686	0,275323057	0,353350742	0
9433	H200014759	SMO	-5,49685478	-1,76912896	0,321843860	0,300979090	0
11894	H200021322	PHF20	-5,49536181	-1,60488312	0,292043213	0,320186267	0
2894	H200003722	PIGL	-5,49041064	-1,30512983	0,237710786	0,408294573	0
8476	H200012831	TNFSF14	-5,47186183	-2,05191098	0,374993200	0,259522551	0
1954	H200002444	THTPA	-5,46878283	-1,23531613	0,225885021	0,423844011	0
11256	H200019375	GOLGA7	-5,46252196	-1,62575970	0,297620717	0,329197082	0
8732	H200013423	LRRC45	-5,45744538	-1,54613675	0,283307783	0,345691293	0
4119	H200005514	HACL1	-5,45714671	-1,30002945	0,238225123	0,410364475	0
2223	H200002801	RAB22A	-5,45341177	-1,24480109	0,228260976	0,427444514	0
4650	H200006260	CEBPA	-5,45198340	-1,32764542	0,243516043	0,406478018	0
11506	H200020060	LRRC23	-5,45078994	-1,71068352	0,313841396	0,303423932	0
1929	H200002413	SLCO3A1	-5,44656070	-1,42523159	0,261675518	0,363557312	0
5867	H200008000	DHX8	-5,44529346	-1,71185685	0,314373663	0,314930749	0
3743	H200004939	FBXL4	-5,44326327	-1,25176587	0,229966071	0,415723889	0
6166	H200008418	MRPS16	-5,42464375	-1,54416099	0,284656664	0,342440380	0
8255	H200012277	TAF1B	-5,42021648	-1,57200647	0,290026510	0,345780396	0
7599	H200011182	CAPNS2	-5,42010548	-1,38046858	0,254694043	0,384606552	0
1810	H200002239	B4GALT4	-5,41693764	-1,58361210	0,292344533	0,348508441	0
3403	H200004449	SKIL	-5,41628721	-1,57658316	0,291081897	0,330052410	0
6465	H200008923	CHST11	-5,41419485	-1,72081369	0,317833720	0,302892533	0
6896	H200009934	S100A16	-5,41271527	-1,54259707	0,284995051	0,356455157	0
11487	H200019981	NME6	-5,41167157	-1,25948469	0,232734872	0,422014713	0
10070	H200016268	MAP2K5	-5,40460906	-1,35968675	0,251579113	0,397340323	0
5513	H200007383	C10orf118	-5,40415911	-1,50607382	0,278687912	0,343358882	0
10057	H200016250	HOOK1	-5,40132443	-1,32148058	0,244658619	0,394875233	0
7721	H200011402	ELMO3	-5,38732161	-1,12603583	0,209015891	0,455653785	0
10449	H200017253	PLXNB1	-5,38265053	-1,63557248	0,303860054	0,338301666	0
7612	H200011201	SLC13A3	-5,38010877	-1,73001916	0,321558398	0,279669742	0
4411	H200005960	APLP1	-5,37945268	-1,61410989	0,300050941	0,343121329	0
1908	H200002386	LOC643837	-5,37285704	-1,27127243	0,236610135	0,419970822	0
5282	H200007024	LOXL2	-5,36755491	-1,44840168	0,269843850	0,377905197	0
2990	H200003854	ZCCHC3	-5,36713806	-1,91127707	0,356107304	0,289616045	0
6643	H200009397	FLJ13137	-5,35823613	-1,55118795	0,289496005	0,354847299	0
3551	H200004659	SIRT2	-5,35689850	-1,08200799	0,201984037	0,475239237	0
1292	H200001555	MIZF	-5,34987943	-1,33490301	0,249520205	0,395799053	0
4173	H200005580	SAMD9	-5,34415141	-1,14519245	0,214288922	0,454647261	0

8878	H200013760	KIAA1524	-5,34094094	-1,89439851	0,354693777	0,299357026	0
3359	H200004387	COL9A2	-5,33496137	-1,48844271	0,278997843	0,368194854	0
1923	H200002404	C16orf3	-5,32074831	-1,49439823	0,280862417	0,371455505	0
2879	H200003701	EAF2	-5,31768425	-1,51893561	0,285638549	0,346820256	0
6043	H200008225	CNNM4	-5,31397122	-1,38398889	0,260443430	0,373837313	0
8964	H200013889	RPGR	-5,30747425	-1,67119729	0,314876194	0,320131776	0
11167	H200019144	DICER1	-5,28922011	-1,20332635	0,227505440	0,434138954	0
7016	H200010185	ANKRD15	-5,28809396	-1,70802178	0,322993842	0,283549630	0
5595	H200007577	PLEKHG6	-5,28796178	-1,44305955	0,272895231	0,381175061	0
3672	H200004835	RBJ	-5,28478929	-1,24747279	0,236049673	0,427182335	0
1363	H200001650	MAGEE1	-5,27782332	-1,31200780	0,248588806	0,398608046	0
3648	H200004801	KCNMB4	-5,27663420	-1,23183852	0,233451566	0,432567756	0
7078	H200010289	CENPQ	-5,27631610	-1,15028629	0,218009358	0,443892470	0
2899	H200003728	ERC1	-5,26998235	-1,28190358	0,243246275	0,403647509	0
8375	H200012575	LRCH4	-5,26565546	-1,41563443	0,268842965	0,384544269	0
3535	H200004636	MAK10	-5,26460902	-1,43467852	0,272513782	0,368660425	0
9118	H200014118	CREM	-5,26301196	-1,40270562	0,266521458	0,371082805	0
6616	H200009354	C10orf57	-5,26234641	-1,43394371	0,272491318	0,367191722	0
2260	H200002851	ARHGAP9	-5,25982297	-1,37433075	0,261288405	0,399035840	0
6240	H200008514	DNAJB6	-5,25462343	-1,43311578	0,272734250	0,368803801	0
8769	H200013521	DDX58	-5,25439060	-1,33355370	0,253797976	0,389847402	0
1451	H200001764	PDCL	-5,24825386	-1,57594888	0,300280611	0,353690456	0
5194	H200006920	MTR	-5,24198870	-1,38348924	0,263924500	0,398434461	0
6228	H200008498	BRMS1L	-5,24191602	-1,41622608	0,270173364	0,389736339	0
1294	H200001560	RPS10	-5,24181438	-1,40930398	0,268858048	0,388253980	0
2676	H200003424	TAF4	-5,23630075	-1,21228679	0,231515883	0,435265733	0
3566	H200004679	APITD1	-5,23048611	-1,64522864	0,314546030	0,338725873	0
7627	H200011226	SPATA20	-5,22875218	-1,30932784	0,250409237	0,414220216	0
8723	H200013399	C12orf43	-5,22448051	-1,48759951	0,284736350	0,357607187	0
7744	H200011436	KLHL20	-5,21950922	-1,46651697	0,280968365	0,372901710	0
10123	H200016408	PCSK1N	-5,21784209	-1,55596016	0,298199933	0,357750917	0
11071	H200018994	C7orf23	-5,21603446	-1,55324569	0,297782867	0,337839005	0
5532	H200007429	GOLGA2L1	-5,21318155	-1,77028917	0,339579421	0,320328639	0
8610	H200013127	FAM20C	-5,20226207	-1,55443321	0,298799482	0,357599693	0
5779	H200007869	TCF2	-5,19512023	-1,71395567	0,329916459	0,277718689	0
6223	H200008493	ZNF346	-5,19409494	-1,62047136	0,311983393	0,313469123	0
7620	H200011212	ZNF610	-5,19062264	-1,25506476	0,241794645	0,413144210	0
4075	H200005443	GEFT	-5,18807560	-1,57983612	0,304512934	0,354867456	0
2824	H200003626	CCDC22	-5,18713586	-1,52959540	0,294882464	0,352816492	0
5735	H200007805	CRYBB2	-5,18638642	-1,25944594	0,242836889	0,425801711	0
4382	H200005923	FABP6	-5,18032852	-1,58898928	0,306735233	0,319951517	0
1644	H200002007	LEPR	-5,16837661	-1,32150131	0,255689825	0,402783360	0
5847	H200007975	SCML2	-5,16406185	-1,49483498	0,289468838	0,339052292	0
7520	H200011039	ERCC2	-5,16335727	-1,83994847	0,356347310	0,307030000	0
4211	H200005648	ZNF673	-5,15374627	-1,64828923	0,319823512	0,324566898	0
5188	H200006909	SEMA3B	-5,14832242	-1,37346236	0,266778622	0,398727433	0
6928	H200010006	KLHL25	-5,14444707	-1,59830137	0,310684773	0,334645987	0
7005	H200010163	ZNF169	-5,14301680	-1,65235497	0,321281271	0,339146577	0
8253	H200012275	ZNHIT2	-5,13580734	-1,42278088	0,277031592	0,382844219	0
4737	H200006369	FHIT	-5,13138588	-1,73118573	0,337371964	0,278768600	0
6904	H200009956	KIF18A	-5,12725075	-1,29274901	0,252132980	0,414664851	0
2203	H200002775	TLK1	-5,11690002	-1,39799360	0,273211044	0,389343353	0
3657	H200004814	TCFL5	-5,10882754	-1,56447108	0,306228986	0,333179459	0
8467	H200012809	ANKRD26	-5,10525510	-1,44595089	0,283227940	0,366125156	0
10467	H200017284	PPM1F	-5,09779327	-1,45928671	0,286258512	0,356751403	0
1873	H200002339	KCTD6	-5,09648822	-1,31106827	0,257249348	0,412161508	0
8724	H200013406	PURB	-5,09544838	-1,72062788	0,337679385	0,290448310	0
4077	H200005447	STEAP1	-5,07975504	-1,43348041	0,282194790	0,376837460	0
3106	H200004008	RNF24	-5,07739281	-1,25681341	0,247531255	0,420431699	0
8345	H200012521	TCL1B	-5,07236734	-1,46698196	0,289210514	0,375665631	0
10191	H200016553	L3MBTL2	-5,07018190	-1,27965196	0,252387781	0,416681641	0
1963	H200002458	PEMT	-5,06941868	-1,37665338	0,271560403	0,391547663	0
7781	H200011497	ING2	-5,06728422	-1,40201694	0,276680147	0,379202213	0
9236	H200014323	SHF	-5,06609988	-1,36558931	0,269554360	0,385952083	0
10897	H200018367	PQLC3	-5,06260628	-1,27064652	0,250986637	0,422181387	0
8184	H200012156	C8orf70	-5,06005660	-1,30783766	0,258463049	0,413650853	0
10032	H200016211	ZNF219	-5,05727617	-1,42861713	0,282487466	0,375049850	0
11699	H200020672	ZNF429	-5,05573472	-1,49422191	0,295549904	0,354368998	0
2369	H200002998	COL4A3BP	-5,04510564	-1,13812869	0,225590657	0,454153821	0
11005	H200018833	LOC727918	-5,03963041	-1,29332600	0,256631120	0,391541085	0
2667	H200003412	RAB11FIP5	-5,03962474	-1,40801121	0,279388106	0,390335694	0
4625	H200006230	ANK3	-5,03805791	-1,96000743	0,389040273	0,285580749	0
4123	H200005521	PTPN6	-5,03587997	-1,36395359	0,270847120	0,384769602	0
9867	H200015660	RSC1A1	-5,03572410	-1,42842989	0,283659284	0,370776874	0
1654	H200002018	CCDC113	-5,03415247	-1,23206233	0,244740766	0,421587571	0
9173	H200014231	CHMP4C	-5,03112440	-1,65955745	0,329858163	0,341505975	0
5340	H200007094	NPTX1	-5,02648965	-1,42956325	0,284405888	0,386051453	0
6805	H200009687	C18orf43	-5,02486709	-1,32398436	0,263486444	0,399398289	0
9009	H200013953	BACH1	-5,02435166	-1,31574722	0,261874031	0,413472747	0
4607	H200006212	PMM1	-5,01952104	-1,31165087	0,261309965	0,411998508	0
7515	H200011029	RTN1	-5,01511936	-1,33110651	0,265418711	0,409233227	0
5240	H200006978	PTPN2	-5,01508760	-1,34564620	0,268319580	0,387881433	0
4036	H200005370	CDC2L5	-5,00619134	-1,39405017	0,278465219	0,393157616	0
1712	H200002093	MID2	-5,00450855	-1,45080618	0,289899830	0,383606394	0
11314	H200019502	DNAJC4	-5,00310638	-1,34354165	0,268541492	0,381450293	0
5645	H200007660	IRF7	-5,00290531	-1,28393882	0,256638641	0,418948412	0
6046	H200008241	PAWR	-4,98931884	-1,18673573	0,237855261	0,443227066	0
5656	H200007678	HEXA	-4,98771872	-1,36138062	0,272946551	0,387442144	0
5817	H200007923	SCN1B	-4,98559918	-1,51513289	0,303901866	0,370422407	0
9265	H200014379	MORN1	-4,98499723	-1,18861196	0,238437838	0,434888429	0
1809	H200002237	FLJ21963	-4,97805216	-1,62670110	0,326774621	0,341102628	0

6765	H200009627	MND1	-4,97722025	-1,80083045	0,361814498	0,306816678	0
7308	H200010644	ZNF580	-4,97268344	-1,67475540	0,336791075	0,285058067	0
7640	H200011251	LOC157503	-4,97152440	-1,71030377	0,344019990	0,332195142	0
10374	H200017004	DSCR1L2	-4,97042504	-1,42223726	0,286139968	0,382018692	0
576	H200000679	SCNN1A	-4,97006473	-1,20971204	0,243399654	0,419969996	0
5522	H200007408	ZXDA	-4,96685467	-1,34901537	0,271603553	0,402337921	0
11018	H200018866	HNRPAB	-4,96425205	-1,40434565	0,282891690	0,373815843	0
10645	H200017628	GBA	-4,96145922	-1,29303859	0,260616592	0,394739493	0
8698	H200013350	GNL3L	-4,95824509	-1,47713824	0,297915536	0,360375282	0
3019	H200003894	HSPBAP1	-4,95064763	-1,30044209	0,262681207	0,399877565	0
5075	H200006771	MAPK8IP2	-4,94253785	-1,41441876	0,286172570	0,368162211	0
2744	H200003520	TOLLIP	-4,94214561	-1,43332260	0,290020310	0,386638441	0
2187	H200002754	N4BP2	-4,93911249	-1,02090678	0,206698426	0,494525961	0
6396	H200008786	MRFAP1L1	-4,93423991	-1,50351626	0,304710814	0,365466092	0
5798	H200007894	PDGFRL	-4,93200007	-1,56835234	0,317995198	0,338785460	0
9033	H200013994	PDPK1	-4,92503916	-1,16266085	0,236071392	0,452916952	0
5196	H200006922	PHF16	-4,92154475	-1,37981495	0,280362166	0,382281372	0
11665	H200020498	THAP8	-4,91961223	-1,30442278	0,265147479	0,421387318	0
7276	H200010592	LZTS1	-4,90950961	-1,35537592	0,276071548	0,404628697	0
10550	H200017479	MSRB2	-4,90868288	-1,40189269	0,285594471	0,377048845	0
4207	H200005643	SPHK1	-4,90738951	-1,42849940	0,291091505	0,365263864	0
9900	H200015717	ATPIF1	-4,90579973	-1,67187690	0,340795995	0,326098913	0
5533	H200007432	C3orf9	-4,89482429	-1,10658742	0,226072961	0,467603679	0
5819	H200007926	TP53BP1	-4,89396117	-1,18075193	0,241267122	0,441514296	0
8592	H200013091	ZNF358	-4,89280177	-1,10044863	0,224911754	0,464168634	0
5894	H200008032	MGAT2	-4,88787612	-1,41045964	0,288562887	0,388910461	0
6045	H200008236	PRIC285	-4,87749595	-1,01663159	0,208433099	0,492781765	0
9171	H200014229	TRIM24	-4,87367804	-1,68856114	0,346465467	0,280290121	0
8531	H200012958	RNF121	-4,87092073	-1,47617662	0,303059053	0,362602901	0
11878	H200021300	SORT1	-4,86630009	-1,13246520	0,232715858	0,463666811	0
8570	H200013034	C1orf182	-4,86125707	-1,32899686	0,273385431	0,391810073	0
2246	H200002830	MECR	-4,85743958	-1,11786494	0,230134605	0,465828563	0
3813	H200005038	SFRS2IP	-4,84953250	-1,13467746	0,233976670	0,459077882	0
7964	H200011759	ROBO4	-4,84704713	-1,08445240	0,223734652	0,476494790	0
5748	H200007827	ZNF398	-4,84646817	-1,39265845	0,287355327	0,398673210	0
11352	H200019620	HVCN1	-4,84559432	-1,21886952	0,251541801	0,424664221	0
2783	H200003567	RASD1	-4,83880783	-1,30634617	0,269972731	0,419908071	0
3707	H200004887	ZNF228	-4,83840548	-1,19586510	0,247160991	0,442334023	0
2477	H200003146	STK11IP	-4,83790361	-1,32038603	0,272925246	0,403074454	0
10345	H200016958	FAM120C	-4,83735189	-1,79935095	0,371970240	0,319981340	0
795	H200000937	CCNL1	-4,83477904	-1,27428130	0,263565572	0,420894106	0
527	H200000618	CD302	-4,82664906	-1,15563030	0,239427041	0,458758499	0
6627	H200009372	GNL3L	-4,82652048	-1,25276458	0,259558534	0,414190386	0
7111	H200010342	BPI	-4,82613338	-1,65938404	0,343833024	0,291495312	0
11499	H200020042	C11orf72	-4,82073699	-1,43925178	0,298554305	0,384350960	0
7041	H200010226	ARHGAP22	-4,80758161	-1,19783111	0,249154607	0,424522151	0
10359	H200016975	C1orf112	-4,80334240	-1,19000180	0,247744529	0,426274666	0
215	H200000255	SLC10A1	-4,79809814	-1,52233620	0,317279088	0,371663841	0
126	H200000147	FUCA1	-4,79445814	-1,31954147	0,275222232	0,409405993	0
6253	H200008536	UFD1L	-4,79099341	-1,02982811	0,214950850	0,488482002	0
3012	H200003885	GLRX	-4,79039395	-1,38637450	0,289407200	0,393594172	0
11846	H200021207	PDCD6	-4,78878875	-1,17337219	0,245024838	0,455506238	0
4972	H200006646	CD83	-4,78840835	-1,42036922	0,296626587	0,375626528	0
1765	H200002180	ACOX3	-4,78463018	-1,51130707	0,315867060	0,366228753	0
2559	H200003253	MPP2	-4,78392116	-1,07081014	0,223835240	0,477820743	0
4152	H200005557	RNF113A	-4,78353350	-1,37430268	0,287298642	0,376283690	0
3770	H200004975	RABL4	-4,78157350	-1,30313274	0,272532198	0,395137414	0
10749	H200017941	LSG1	-4,77234878	-1,28284727	0,268808366	0,415524933	0
4265	H200005730	LYAR	-4,77048013	-1,75407966	0,367694574	0,315173924	0
7432	H200010904	DOK6	-4,76196381	-1,07357908	0,225448812	0,477168956	0
9986	H200016008	VPREB1	-4,76129478	-1,53149184	0,321654490	0,352342067	0
53	H200000059	LTK	-4,76040865	-1,42384282	0,299100965	0,364112004	0
10465	H200017280	TRFP	-4,75614471	-1,23378850	0,259409369	0,436236505	0
8915	H200013820	FLJ20674	-4,75079828	-1,39313157	0,293241574	0,395908666	0
2213	H200002790	FCHSD2	-4,75047676	-1,30322621	0,274335878	0,404478306	0
5260	H200007000	GRB14	-4,74718198	-1,02797040	0,216543288	0,496380583	0
830	H200000977	C19orf4	-4,74542979	-1,22834049	0,258847047	0,416075365	0
6204	H200008466	ZNF403	-4,74290057	-1,38094566	0,291160577	0,379021899	0
8678	H200013299	ZNF337	-4,73476472	-1,40668631	0,297097404	0,391399508	0
343	H200000397	IGHMBP2	-4,71432842	-1,24004465	0,263037392	0,412742409	0
2989	H200003853	LYRM1	-4,70759257	-1,10682513	0,235114894	0,449609335	0
6337	H200008660	TTC12	-4,70629476	-1,45902597	0,310015850	0,384565776	0
6845	H200009835	FLJ10159	-4,70599591	-1,19139489	0,253165305	0,445937170	0
8490	H200012873	IQSEC2	-4,69991282	-1,76299094	0,375111412	0,316478324	0
8325	H200012476	PRDM15	-4,69243727	-1,31653557	0,280565406	0,400951413	0
9955	H200015858	PIGB	-4,68659330	-1,15537443	0,246527564	0,451365067	0
11530	H200020115	C6orf192	-4,68140873	-1,26185277	0,269545524	0,422846836	0
6101	H200008329	UTP20	-4,67500150	-1,26447949	0,270476810	0,415654432	0
3135	H200004045	ASTN2	-4,65661155	-1,48616291	0,319151146	0,363721096	0
2008	H200002515	ACP6	-4,65351878	-1,27778878	0,274585501	0,420755441	0
7125	H200010365	PTHLH	-4,65267605	-1,31763463	0,283199306	0,419395459	0
4195	H200005623	HOXB13	-4,64344616	-1,13573352	0,244588497	0,451056888	0
10109	H200016366	ZCCHC6	-4,64176610	-1,05888883	0,228121970	0,472732811	0
7422	H200010875	GLIPR1L1	-4,63985316	-1,32018166	0,284530915	0,398845391	0
4183	H200005606	TOP3B	-4,63892716	-1,42912567	0,308072452	0,376075021	0
10344	H200016956	RNF125	-4,63621330	-1,28726975	0,277655421	0,421069302	0
4166	H200005572	CLEC3B	-4,63251126	-1,45092974	0,313205874	0,350983515	0
9244	H200014336	GBX2	-4,62819453	-1,24734991	0,269511124	0,418474620	0
3915	H200005180	FLJ22531	-4,62601353	-1,32965575	0,287430148	0,397299632	0
6140	H200008384	SLC25A12	-4,62510975	-1,23448375	0,266909073	0,432076919	0
5776,00	H200007865	SSBP2	-4,61965211	-0,83865613	0,181540972	0,562004416	0

6062	H200008270	PMS2	-4,61914985	-1,26537800	0,273941751	0,413644711	0
10770	H200017987	NUDT5	-4,61460369	-1,33429665	0,289146530	0,370583491	0
3064	H200003954	PRR8	-4,61240620	-1,32553266	0,287384199	0,401751912	0
9225	H200014308	SNX24	-4,61204770	-1,19778359	0,259707546	0,424131843	0
7474	H200010979	SNIP1	-4,60579708	-1,48014883	0,321366489	0,369892126	0
7464	H200010965	ZBTB32	-4,60353764	-1,34756499	0,292723792	0,409850356	0
1891	H200002362	SRBD1	-4,59954352	-1,20897978	0,262847774	0,428013302	0
7579	H200011146	MR1	-4,59938614	-1,10160008	0,239510240	0,469531393	0
2031	H200002539	CLDN2	-4,59892755	-1,21982962	0,265242192	0,424846678	0
6893	H200009927	C1orf26	-4,59806766	-1,05028444	0,228418657	0,489108352	0
7587	H200011162	TULP4	-4,59786510	-1,32170620	0,287460847	0,385661522	0
3761	H200004963	CCL19	-4,59235186	-1,21522597	0,264619525	0,442431458	0
4771	H200006410	IVD	-4,58985334	-1,38691508	0,302169803	0,402604278	0
9724	H200015402	CCDC9	-4,57981551	-1,27538622	0,278479825	0,427057943	0
4340	H200005864	NCLN	-4,57781839	-1,12900171	0,246624398	0,457889854	0
3970	H200005260	LRRC6	-4,57348019	-1,25475164	0,274353793	0,393700385	0
7810	H200011539	CADPS2	-4,57022849	-1,22015035	0,266977975	0,411256419	0
8986	H200013918	CBFA2T2	-4,56467838	-1,38069064	0,302472710	0,382370482	0
10452	H200017256	ACSL1	-4,55931385	-1,44387234	0,316686324	0,374005433	0
4921	H200006590	BCKDHA	-4,55234659	-1,08903458	0,239224882	0,469500603	0
5903	H200008041	ZNF451	-4,53783163	-1,52571791	0,336221798	0,340423506	0
2214	H200002792	LATS2	-4,53672368	-1,15703648	0,255037900	0,443927633	0
3320	H200004326	GRIN2C	-4,53520657	-1,02299074	0,225566514	0,497219696	0
11496	H200020006	NSFL1C	-4,53144408	-1,26118650	0,278318893	0,405546011	0
2302	H200002910	C6orf60	-4,53109910	-1,04688883	0,231045229	0,487943411	0
3013	H200003887	LRRC35	-4,53027488	-1,04596226	0,230882736	0,491098936	0
8073	H200011970	DCLRE1B	-4,52889191	-1,21638126	0,268582532	0,423484290	0
6783	H200009656	C12orf4	-4,52754115	-1,16445274	0,257193187	0,458390509	0
4774	H200006413	HLA-DMB	-4,52692085	-1,35461766	0,299235994	0,394325994	0
1661	H200002029	FRMD5	-4,52670369	-1,30004454	0,287194530	0,420143059	0
1462	H200001778	ZNF84	-4,52218762	-1,32215068	0,292369709	0,376033072	0
6571	H200009239	ZC3H12A	-4,51796860	-1,29252120	0,286084591	0,410232973	0
5895	H200008033	ADCY7	-4,51450386	-1,44902324	0,320970650	0,337531385	0
3947	H200005229	TNRC5	-4,51334634	-1,09045355	0,241606443	0,462102640	0
7452	H200010950	NPFFR2	-4,49962288	-1,27648226	0,283686499	0,392254874	0
3195	H200004119	MAP3K7IP1	-4,49894185	-1,08863509	0,241975808	0,480961251	0
10279	H200016721	TUBD1	-4,49827228	-1,81441472	0,403358134	0,330556623	0
7025	H200010198	ZNF254	-4,48503026	-1,60557277	0,357984826	0,332301475	0
11321	H200019514	C10orf58	-4,48222065	-1,46866181	0,327663880	0,392339013	0
10744	H200017929	PHF20	-4,47926081	-1,53650021	0,343025395	0,373166607	0
1805	H200002232	SENP5	-4,47230620	-1,00746969	0,225268495	0,496205467	0
6263	H200008549	GRIK1	-4,46692243	-1,06960097	0,239449192	0,485850325	0
1002	H200001184	ARL3	-4,46110049	-1,18305905	0,265194440	0,422342514	0
2134	H200002683	TNFAIP8	-4,45772467	-1,23813558	0,277750573	0,436357739	0
11656	H200020475	C1orf163	-4,45624435	-1,37596460	0,308772250	0,372204503	0
8837	H200013681	JMJD2C	-4,45355021	-1,44921606	0,325406921	0,394616774	0
763	H200000894	FAM77C	-4,45217230	-1,13694430	0,255368442	0,459339590	0
8933	H200013844	CDS1	-4,44881211	-1,14545364	0,257474042	0,464710582	0
516	H200000604	TPD52	-4,44373025	-1,08621191	0,244436960	0,478624945	0
11217	H200019289	FBXO38	-4,44019868	-1,43239799	0,322597723	0,360420168	0
624	H200000732	SMARCA3	-4,44013290	-1,29548631	0,291767462	0,391362811	0
629	H200000738	KARS	-4,43047693	-1,28118536	0,289175494	0,408690044	0
5920	H200008062	ARHGAP11A	-4,42815861	-1,08529701	0,245089913	0,456327354	0
173	H200000204	TRIM23	-4,42635371	-1,07264028	0,242330448	0,482669478	0
9308	H200014475	RSRC1	-4,42495873	-1,42263536	0,321502514	0,392548324	0
9968	H200015874	RHO	-4,42474527	-1,52076665	0,343695865	0,356040237	0
3417	H200004469	RPL9	-4,42380645	-1,13209188	0,255908998	0,462521490	0
6051	H200008254	SPATA7	-4,41082434	-1,15491698	0,261836993	0,459171719	0
2622	H200003349	SLC22A6	-4,41039456	-1,43283598	0,324877052	0,384499665	0
11188	H200019176	ELOF1	-4,41015219	-1,24987467	0,283408512	0,435632533	0
800	H200000942	SLC12A6	-4,40746913	-1,32712373	0,301107891	0,410671180	0
6925	H200010003	DBP	-4,40745682	-1,12433713	0,255098842	0,456898257	0
9402	H200014696	MRM1	-4,40474758	-1,37356602	0,311837625	0,389871368	0
5827	H200007941	TMOD1	-4,40020994	-1,27106579	0,288864806	0,414569093	0
9519	H200014921	ZNF253	-4,39921302	-1,80282283	0,409805758	0,324249959	0
10695	H200017792	CHODL	-4,39772130	-1,03725292	0,235861449	0,499153575	0
4555	H200006136	CENPE	-4,39315599	-1,34461343	0,306070040	0,416813347	0
2447	H200003110	SMC1A	-4,38068097	-1,40023996	0,319639793	0,360019491	0
9799	H200015558	HIPK2	-4,37392853	-1,28246508	0,293206684	0,411590494	0
2633	H200003367	C13orf25	-4,37263564	-1,29353326	0,295824616	0,406198545	0
11813	H200021115	RFWD3	-4,36594904	-1,33146563	0,304965911	0,381596754	0
8917	H200013822	UGCG	-4,36472561	-1,37678743	0,315435048	0,375192701	0
5248	H200006986	CUL2	-4,35958481	-1,02562772	0,235258118	0,490888523	0
8508	H200012906	AFF3	-4,34916510	-1,11723940	0,256885949	0,463358161	0
9934	H200015812	SLC2A6	-4,34836667	-1,28540592	0,295606607	0,412422330	0
2618	H200003345	CEECAM1	-4,34797877	-1,59120030	0,365963218	0,314692978	0
9159	H200014205	PPFIA1	-4,34580892	-1,12803108	0,259567574	0,466261045	0
4163	H200005569	LIPG	-4,34512790	-1,09019228	0,250899929	0,476861654	0
7729	H200011417	TUT1	-4,33815088	-1,31153752	0,302326396	0,416742542	0
9670	H200015286	DHDDS	-4,32816669	-1,32362245	0,305815961	0,416172252	0
6366	H200008725	G6PC2	-4,32539606	-1,13862095	0,263240853	0,464303675	0
7913	H200011689	REV1L	-4,32361015	-1,06384858	0,246055620	0,461625401	0
11765	H200020982	C10orf86	-4,31681543	-1,28821904	0,298418836	0,384514914	0
6870	H200009884	COQ3	-4,31664869	-1,08946435	0,252386615	0,473624957	0
9815	H200015584	C12orf35	-4,31383372	-1,16527378	0,270124872	0,463454228	0
10510	H200017355	OPTN	-4,31001685	-1,22361454	0,283900175	0,444944283	0
2644	H200003384	LRIG2	-4,30311956	-1,09303169	0,254009138	0,457270549	0
10019	H200016173	ATP6V1G2	-4,29913905	-1,08837838	0,253161940	0,475792404	0
658	H200000776	ASB6	-4,29267719	-1,03204817	0,240420635	0,489314185	0
5511	H200007378	VPS13B	-4,29186600	-1,23312082	0,287315777	0,400561018	0
8445	H200012760	SNX16	-4,29148190	-1,21801773	0,283822176	0,405250002	0

1275	H200001530	BTBD3	-4,28859910	-1,28287240	0,299135538	0,390830165	0
5535	H200007438	GPR180	-4,28292216	-1,04309685	0,243547934	0,484470536	0
10005	H200016139	CXorf56	-4,28206890	-1,39178717	0,325026805	0,407909530	0
6458	H200008893	WDR55	-4,28034601	-1,07948128	0,252194865	0,466600168	0
1035	H200001226	SYN2	-4,27600038	-1,23129312	0,287954399	0,404302805	0
6288	H200008590	HIST1H2BM	-4,27413286	-1,28079999	0,299663119	0,428793854	0
9364	H200014631	TERF1	-4,26945152	-1,25029590	0,292846960	0,441833412	0
2109	H200002646	NFU1	-4,26868668	-1,23497110	0,289309380	0,447040252	0
11084	H200019018	PXK	-4,26859968	-1,13249468	0,265308244	0,449016746	0
7299	H200010628	OMD	-4,26824392	-1,22194658	0,286287897	0,429772458	0
8619	H200013147	ASAM	-4,26039252	-1,11595920	0,261938117	0,475970393	0
10504	H200017346	BCOR	-4,25803153	-1,05298841	0,247294647	0,493761208	0
6718	H200009533	WDR85	-4,25659088	-1,39199653	0,327021451	0,368144560	0
8781	H200013557	CHEK2	-4,25614577	-1,22472061	0,287753446	0,442577228	0
6556	H200009222	FRAS1	-4,25420516	-1,13869447	0,267663272	0,466086423	0
9568	H200015057	RXFP1	-4,24185712	-1,12987019	0,266362153	0,465147940	0
6088	H200008312	TSR2	-4,24056437	-1,28559031	0,303164910	0,420661687	0
11331	H200019543	RAXL1	-4,23173067	-1,11354659	0,263142122	0,474554249	0
9849	H200015636	SFRS1	-4,23110805	-1,37284880	0,324465550	0,410706635	0
3398	H200004444	CLDN15	-4,23059985	-1,48848128	0,351836933	0,344379234	0
11602	H200020367	FAM48A	-4,22711248	-1,31816203	0,311835098	0,419100920	0
4850	H200006509	COL18A1	-4,22584617	-1,14275378	0,270420109	0,470562406	0
9976	H200015945	TFAP2D	-4,22538524	-1,02134178	0,241715659	0,489689525	0
6264	H200008552	CC2D1A	-4,21048608	-1,11844103	0,265632284	0,469412830	0
7115	H200010346	SELE	-4,20375536	-1,35210895	0,321643111	0,402049753	0
9989	H200016056	CHRNG	-4,19977132	-1,05901366	0,252159839	0,487795119	0
5078	H200006775	TCEA2	-4,19591550	-1,15292591	0,274773387	0,447771621	0
9818	H200015587	C1orf69	-4,19496386	-1,42044556	0,338607342	0,370895925	0
5012	H200006692	MPG	-4,19443958	-1,19270180	0,284353076	0,447856649	0
6552	H200009213	FUSIP1	-4,19433631	-1,17252187	0,279548845	0,465284250	0
3129	H200004039	HRBL	-4,19419096	-1,28259851	0,305803557	0,427413279	0
5197	H200006923	RGS3	-4,18772790	-1,35845151	0,324388676	0,399409516	0
502	H200000588	CHRNB2	-4,18298145	-1,05756812	0,252826394	0,467045489	0
5258	H200006998	PEX11B	-4,17665820	-1,29922092	0,311067091	0,431337524	0
5330	H200007083	ANKHD1	-4,17584450	-1,29568195	0,310280219	0,410613697	0
11151	H200019114	SMA5	-4,17536582	-1,27120816	0,304454321	0,423206164	0
116	H200000134	NR2C2	-4,17301986	-1,39528691	0,334359040	0,351307648	0
5963	H200008111	SLC24A1	-4,17202170	-1,15826682	0,277627227	0,448381433	0
5474	H200007302	PKNOX1	-4,16281249	-1,04897321	0,251986658	0,495684618	0
2288	H200002891	KBTBD2	-4,14457961	-1,12342549	0,271058972	0,464935115	0
10223	H200016601	RNUXA	-4,14288111	-1,04610523	0,252506699	0,480011213	0
3030	H200003908	ZNF584	-4,12626138	-1,15062285	0,278853602	0,468216065	0
8893	H200013778	CRY1	-4,12603451	-1,12709746	0,273167241	0,474897639	0
3113	H200004020	MVP	-4,12170933	-1,24251783	0,301456926	0,444827147	0
10812	H200018145	FKBP9L	-4,11864903	-1,21633186	0,295323018	0,435517909	0
8780	H200013556	VPS36	-4,11704090	-1,42222281	0,345447823	0,385968014	0
22	H200000021	AZGP1	-4,11632010	-1,33455690	0,324211157	0,422727593	0
8949	H200013866	DOM3Z	-4,11238063	-1,35540885	0,329592265	0,419672097	0
2585	H200003294	KIAA1468	-4,11224127	-1,19863680	0,291480173	0,429617641	0
6561	H200009228	TROVE2	-4,10990932	-1,09263083	0,265852782	0,471848885	0
5919	H200008061	DLX4	-4,09823945	-1,40522607	0,342885301	0,360720217	0
7513	H200011026	KRT10	-4,09752564	-1,61661186	0,394533677	0,350566697	0
4877	H200006542	NSMAF	-4,09495048	-1,03649385	0,253115112	0,495332546	0

11175	H200019157	LOC284701	-4,09148970	-1,10713582	0,270594797	0,463920226	0
1221	H200001466	HIST1H1C	-4,09110120	-1,17621993	0,287506925	0,420389684	0
6058	H200008266	DUSP10	-4,09049905	-1,41214725	0,345226154	0,390919233	0
7961	H200011755	CAMK2D	-4,08597246	-1,21032307	0,296214202	0,419584081	0
6214	H200008481	NCAPH2	-4,08582150	-1,05909158	0,259211416	0,488418598	0
3380	H200004416	ZNF77	-4,08482951	-1,15486511	0,282720518	0,442486171	0
8573	H200013039	C1orf97	-4,07804978	-1,38055717	0,338533674	0,410045428	0
5723	H200007783	C12orf32	-4,07674371	-1,17188754	0,287456760	0,461940559	0
5331	H200007084	BCL2L11	-4,07658622	-1,10093271	0,270062412	0,463361192	0
9334	H200014548	BIN1	-4,07277193	-1,09014289	0,267666079	0,481523290	0
650	H200000766	PSEN1	-4,07060240	-1,13878288	0,279757825	0,474743544	0
6663	H200009430	MLL3	-4,07048543	-1,34212894	0,329722084	0,374739411	0
1081	H200001284	JMJD1C	-4,06699338	-1,21986575	0,299942891	0,441315553	0
3062	H200003948	PLEKHF2	-4,06692100	-1,35480899	0,333128919	0,359685726	0
8750	H200013467	SH3RF2	-4,06328118	-1,17033175	0,288026278	0,442190950	0
7327	H200010674	C10orf35	-4,06244858	-1,31987761	0,324897063	0,420603735	0
8680	H200013305	ABCC4	-4,06193984	-1,27147356	0,313021269	0,380998010	0
598	H200000703	PRM1	-4,06015485	-1,31258545	0,323284579	0,407429629	0
7243	H200010539	LOC144438	-4,05848521	-1,18118606	0,291041114	0,417615150	0
420	H200000484	POMC	-4,05776992	-1,46454098	0,360922627	0,390905399	0
9848	H200015635	NADSYN1	-4,05654725	-1,41080090	0,347783673	0,406115107	0
7751	H200011453	NDUFAF1	-4,04531802	-1,17442744	0,290317704	0,456013342	0
7372	H200010746	RHOF	-4,04011654	-1,07386630	0,265800823	0,475680268	0
2047	H200002559	TYW1	-4,03497072	-1,14308379	0,283294196	0,461620401	0
209	H200000247	PRTN3	-4,02886939	-1,31819378	0,327187023	0,388733238	0
7998	H200011810	ARRB1	-4,02639621	-1,28988587	0,320357411	0,408939179	0
3573	H200004690	RAB17	-4,02608204	-1,14729046	0,284964502	0,469407698	0
2717	H200003482	WDR51B	-4,02525169	-1,10507432	0,274535459	0,480264152	0
3455	H200004521	C16orf14	-4,02365835	-1,08767701	0,270320420	0,461677187	0
8028	H200011879	GRIN2D	-4,02194693	-1,03933261	0,258415298	0,497523400	0
7624	H200011220	LSM2	-4,02070323	-1,09977258	0,273527421	0,449572575	0
9303	H200014464	TIMP4	-4,02037874	-1,18051752	0,293633410	0,438917147	0
2898	H200003727	C14orf101	-4,01250537	-0,99826225	0,248787766	0,484809406	0
8048	H200011922	COPS7B	-4,01168557	-1,10019744	0,274248172	0,477375686	0
4533	H200006104	HSD17B4	-4,00887038	-1,28934728	0,321623590	0,435075015	0
8082	H200011985	MANBA	-4,00748979	-1,13556577	0,283360864	0,473151427	0
4076	H200005446	CBARA1	-4,00234306	-1,40189546	0,350268691	0,400139495	0
11336	H200019567	C7orf27	-3,99936694	-1,35702304	0,339309462	0,348771382	0
7528	H200011049	PPOX	-3,99385428	-1,07604382	0,269424907	0,461513058	0
2294	H200002898	IFIT1	-3,98840712	-1,18156401	0,296249598	0,443708487	0
2531	H200003215	NKIRAS2	-3,98833141	-1,21176202	0,303826814	0,414172369	0
5889	H200008025	PYGO2	-3,98457336	-1,04051611	0,261136141	0,499639261	0
11789	H200021047	DPH4	-3,97800593	-1,08520712	0,272801786	0,460481346	0
7628	H200011231	RAB3IP	-3,97614802	-1,16363451	0,292653721	0,466108723	0
7653	H200011280	IGHG1	-3,97601092	-1,01182712	0,254482984	0,496642849	0
6918	H200009985	MLLT10	-3,97321427	-1,25096471	0,314849547	0,425088630	0
11516	H200020092	CCDC128	-3,97241023	-1,13145501	0,284828341	0,452433341	0
6124	H200008359	TAPBP	-3,97171742	-1,21856020	0,306809391	0,434543183	0
1276	H200001531	BAIAP2	-3,97091159	-1,37418659	0,346063255	0,366602820	0
3986	H200005291	FARS2	-3,96793287	-1,15811313	0,291868126	0,466839561	0
6562	H200009229	CTSH	-3,96692711	-1,14914181	0,289680595	0,470034167	0
9122	H200014125	ZNF609	-3,95568908	-1,00449143	0,253935892	0,483755332	0
11391	H200019706	SLC7A6	-3,94939309	-1,34162823	0,339704911	0,421947297	0
5408	H200007184	PRRX2	-3,94585725	-1,28820176	0,326469427	0,441006208	0
4492	H200006054	ST3GAL4	-3,94105130	-1,21338609	0,307883861	0,445787693	0
2908	H200003742	GFER	-3,94014956	-1,13670013	0,288491621	0,469870359	0
10646	H200017630	PLTP	-3,93602683	-1,29324648	0,328566479	0,424437307	0
6872	H200009888	DPYSL5	-3,92983345	-1,44109299	0,366705869	0,332387461	0
8438	H200012731	F2R	-3,51949658	-0,95474520	0,271273228	0,530827206	0
7340	H200010693	NFRKB	-3,14367371	-0,91555635	0,291237715	0,508280505	0

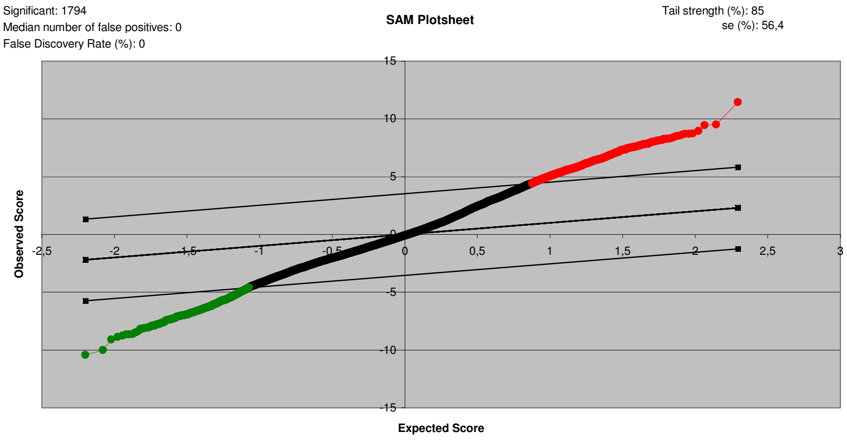

Supplement: Additional file 1 — Complete list of SAM-identified genes with gene name and SAM score. SAM analysis comparing TGCT versus HCT116 cell lines following cisplatin exposure. The positive significant genes (n = 1180, red) are over-expressed in TGCT cells and under-expressed in HCT116 cells. The negative significant genes (n = 614, green) are under-expressed in TGCT cells and over-expressed in HCT116 cells. The complete list of SAM-identified genes is available as supporting information. [file 1476-4598-6-53-S1.pdf]
